# Multicenter randomized double-blind placebo-controlled crossover study of the effect of prolonged noisy galvanic vestibular stimulation on posture or gait in vestibulopathy

**DOI:** 10.1371/journal.pone.0317822

**Published:** 2025-01-24

**Authors:** Chisato Fujimoto, Takuya Kawahara, Yayoi S. Kikkawa, Makoto Kinoshita, Teru Kamogashira, Mineko Oka, Tsukasa Uranaka, Naoya Egami, Kentaro Ichijo, Kayoko Kabaya, Sachiyo Katsumi, Ikumi Takashima, Yoshiharu Yamamoto, Masato Yagi, Tatsuya Yamasoba, Shinichi Iwasaki

**Affiliations:** 1 Department of Otolaryngology and Head and Neck Surgery, Graduate School of Medicine, The University of Tokyo, Bunkyo-ku, Tokyo, Japan; 2 Department of Otolaryngology, Tokyo Teishin Hospital, Chiyoda-ku, Tokyo; 3 Clinical Research Promotion Center, The University of Tokyo Hospital, Bunkyo-ku, Tokyo, Japan; 4 Department of Otolaryngology, Head and Neck Surgery, Nagoya City University Graduate School of Medical Sciences and Medical School, Nagoya, Japan; 5 Educational Physiology Laboratory, Graduate School of Education, The University of Tokyo, Bunkyo-ku, Tokyo, Japan; University of Rochester, UNITED STATES OF AMERICA

## Abstract

**Objective:**

This multicenter, randomized, double-blind, placebo-controlled, crossover trial aimed to evaluate whether prolonged noisy galvanic vestibular stimulation improves body balance in patients with vestibulopathy.

**Materials and methods:**

This trial was registered in the Japan Pharmaceutical Information Center Clinical Trials Information registry (jRCT1080224083). Subjects were 20- to 85-year-old patients who had been unsteady for more than one year and whose symptoms had persisted despite more than six months of rehabilitation. Enrolled subjects were randomly assigned to one of two groups; one group received the optimal intensity of noisy galvanic vestibular stimulation first and then the placebo 14 days later, the other was evaluated in the reverse order. The primary outcome was the difference of the mean percent change from the baseline in the velocity of center of pressure during 3 h of stimulation between the noisy galvanic vestibular stimulation and placebo periods. This was analyzed with the mixed effects model.

**Results:**

Forty-two subjects were enrolled. The mean percent change in the velocity during stimulation for 3 h was -9.3% (SD 19.9%) for noisy galvanic vestibular stimulation and -12.6% (SD 15.0%) for placebo. No significant effects of noisy galvanic vestibular stimulation over placebo were found for velocity in the least-squares means of the difference [3.1% (95% CI -0.2 to 6.4, p = 0.066)].

**Conclusion:**

Prolonged noisy galvanic vestibular stimulation did not significantly improve body balance in patients with poorly-compensated vestibulopathy.

## Introduction

Uncompensated peripheral vestibular dysfunction causes instability in gait and posture, with subjective dizziness and imbalance. The first choice of treatment to promote functional recovery in patients with unilateral and bilateral vestibulopathy (UVP and BVP) is vestibular rehabilitation [1]. However, a certain proportion of patients with vestibulopathy are refractory to rehabilitation and difficult to treat. Although vestibular implants have recently been developed to improve posture, gait, and quality of life in BVP [2], the treatment has a substantial risk of causing sensorineural hearing loss as a complication of surgery [2]. Therefore, the development of minimally invasive treatments for refractory vestibulopathy is desirable.

Noisy galvanic vestibular stimulation (nGVS) delivers electrical current as zero-mean current noise to the vestibular system via electrodes placed bilaterally on the mastoid region. An imperceptible level of nGVS facilitates the processing of subthreshold stimuli in the neural systems including the autonomic, motor and postural control systems [3–6]. nGVS also enhances perceptual thresholds for roll-tilt and inter-aural translation direction [7–9]. Stochastic resonance, in which subthreshold signals are enhanced in the presence of optimal levels of noise in a nonlinear system, has been proposed as a mechanism underlying these ameliorating effects [10,11]. Application of an optimal level of nGVS has been reported to improve standing postural stability and gait performance in BVP patients as well as healthy subjects [4,12–18]. Several studies have shown that nGVS can improve standing postural control for several hours even after the cessation of the stimulus in BVP patients and healthy subjects [3,19–21]. On the other hand, another recent study has reported that nGVS does not improve postural control in BVP patients [22]. These studies in BVP patients were open-label or single-blind studies comparing nGVS to no stimulation [4,14,16–18], and there has been no adequate assessment of a placebo effect or observer bias.

In the present study, a multicenter, randomized, double-blind, placebo-controlled, crossover study was conducted to evaluate the efficacy and safety of prolonged nGVS in improving body balance in UVP and BVP patients with severe postural instability refractory to vestibular rehabilitation.

## Materials and methods

### Standard protocol approvals, registrations, and patient consents

This multicentre, randomised, double-blind, placebo-controlled, crossover study was designed and conducted according to the study protocol, the Declaration of Helsinki, and Japanese Good Clinical Practice. The protocol was approved by the Pharmaceuticals and Medical Devices Agency (PMDA) in Japan and the institutional review board in each participating hospital. The clinical trial notification was submitted to the PMDA by the representative physician. This trial was registered in the Japan Pharmaceutical Information Center (JAPIC) Clinical Trials Information registry (jRCT1080224083; registered on 08/10/2018). The JAPIC clinical trials information registry along with the other two registries and their comprehensive search portals cooperate as an overall system and have been recognized as a primary registry of the World Health Organization—International Clinical Trials Registry Platform (Japan Primary Registries Network). This study was undertaken at three centers in Japan: the University of Tokyo Hospital, Tokyo Teishin Hospital, and Nagoya City University Hospital. The reference numbers of each hospital are: 2018012-11DY for the University of Tokyo Hospital, 241 for Tokyo Teishin Hospital, and 32-21-0002 for Nagoya City University Hospital.

The inclusion criteria were as follows: 1) patients with UVP or BVP on caloric testing using 2 ml of ice water to irrigate the external auditory canal for 20 s, 2) patients with total trajectory length (TTL) of center of pressure (COP) of at least 180 cm in 60 s during posturography (Gravicorder G-620, Anima Inc., Tokyo, Japan) while standing with the eyes closed, 3) patients who had experienced imbalance for more than one year and whose symptoms had persisted despite vestibular rehabilitation therapy for more than six months, 4) patients between 20 and 85 years of age, 5) patients who understood the details of this trial and had given their written informed consent freely and voluntarily prior to participation in this trial.

Caloric nystagmus was recorded using an electronystagmograph. Canal paresis was calculated as the difference between the maximal slow phase eye velocity for each ear divided by the sum of the slow phase eye velocities. UVP referred to canal paresis of 20% or greater [23]; BVP referred to a maximum slow phase velocity of less than 10 deg/s bilaterally [24]. There is no consensus on the definition of severe postural instability in posturography. Therefore, in the present study, we decided to define it from the viewpoint of the upper limit of posturographic findings in healthy elderly subjects. The TTL values in the closed-eye condition of 16 healthy subjects (7 male and 9 female, mean ± SD = 113.2 ± 34.8 cm) aged 65–69 years were extracted from the posturographic data used in our previous study [25]. We set the upper limit of normal at the mean + 2 SD, 182.8 cm. Therefore, in the present study, we defined a severe postural instability as a value greater than 180 cm, which is close to the upper limit of normal for 65–69 year olds.

The exclusion criteria were as follows: 1) patients with metal in their body, such as cerebral artery clips, cochlear implants, or pacemakers (silver teeth were acceptable); 2) patients with orthopedic issues, such as fractures, sprains, separated flesh, acute painful diseases, etc.; 3) patients with limb movement disorders due to cerebellar disorders or spinal cord disease; 4) patients with significant cardiac disease, including severe arrhythmias that may require a pacemaker (atrial fibrillation, severe QT prolongation syndrome, severe atrioventricular block (degree II or higher)), or severe heart failure that interferes with walking; 5) patients with malignant tumors; 6) patients with infectious diseases accompanied by fever, malaise, or dizziness; 7) pregnant and postpartum patients; 8) patients who cannot walk independently; 9) patients with skin abnormalities, such as infections or wounds at the site of application, or patients with a history of anaphylactic shock or other severe allergies; 10) patients with a lack of or limited legal capacity; 11) patients who have participated in another clinical trial (clinical study) in the three months prior to the date of obtaining consent or who will participate in another clinical trial (clinical study) at the same time as this study; 12) other patients who have been judged to be inappropriate as subjects by the investigator (or sub-investigator).

Subjects who met the above inclusion and exclusion criteria were examined for an optimal intensity of nGVS, and those subjects who were found to have an optimal intensity were enrolled in the main study.

### nGVS application

We pretreated the skin with Nuprep Skin Prep Gel (Weaver and Company, Aurora, CO, USA) and applied Blue Sensor NF-00-S (Ambu, Denmark) electrodes to the mastoid region. The conductive medium of this electrode was a solid gel, whose raw materials consisted of polymethyl methacrylate, polyhydric alcohol, electrolyte salt, additives, and deionized water. The electrode material was silver-silver chloride. The surface area of the electrode was 44.8 × 22 mm. The nGVS was applied with electrodes placed bilaterally over the mastoid region by a portable stimulator (112 × 67 × 31 mm; 160 g including dry cells) [4,6]. Waveforms were digitally stored and converted from digital-to-analog at 20 Hz. Zero-mean white noise GVS that ranged from 0.02 to 10 Hz was used. The amplitude crosses 0 mA. Peak to peak amplitude was 2 mA.

### Posturography

In the posturography test, two-legged stance tasks were performed by the subjects in the eyes-closed condition without nGVS and in the eyes-closed condition with nGVS at a sampling frequency of 20 Hz. The following three parameters were measured: the velocity, the area, and the root mean square (RMS) of the COP movement in the XY plane. The mean velocity was TTL divided by measurement time. A fall was defined as a displacement of the foot during posturography testing, and this definition was shared by the three centers.

### Gait analysis

Gait analysis was performed using dynamic gait index (DGI) short form [26] and inertial measurement units (IMUs; WALK-MATE Viewer, WALKMATE LAB., Tokyo, Japan) [27].

DGI short form includes only four items: gait level surface, change in gait speed, gait with horizontal head turns and gait with vertical head turns [26].

IMUs are composed of a triaxial accelerometer (±8 G) and triaxial gyroscope (±1000 deg/s). IMUs were validated against a motion capture system by healthy control participants, as described previously [27,28]. Correlation coefficients between IMU and motion capture measurements were 0.978 for gait speed, 0.983 for stride length, and 0.990 for stride duration [27]. The error between IMU and motion capture measurements for lateral movement distance (trunk) is as low as about 6% [28] IMUs were attached by bands at the ankles bilaterally and at the waist [27]. The following four gait parameters were measured as participants walked a distance of 10 m at their normal pace: gait speed, stride length, stride duration and lateral movement distance (trunk). In our previous study, we found that nGVS for 30 sec increased gait speed, lengthened step length, and shortened stride time in normal subjects and BVP patients [14]. Therefore, we decided to evaluate these parameters in this pivotal trial. In addition, the parameter of lateral movement distance (trunk) was evaluated to see the degree of improvement in lateral trunk sway caused by vestibulopathy. Gait speed, stride length and stride duration were the means of the left and right stride measurements.

### Measurement of optimal intensity

The optimal intensity of nGVS was measured as follows: in a standing position with the eyes closed without nGVS on a posturography platform, the COP was measured three times for 30 s each time at 2-min intervals. The average of these three measurements was used as the baseline measurement. Then, nGVS of 100, 200, 300, 500, 700, 1000, 1200, 1500, 1700 and 2000 μA was applied at 2-min intervals. COP was measured for 30 s in the eyes-closed condition at 100, 200, 300, 500, 700, and 1000 μA. Patients with an optimal intensity were defined as cases in which the two following conditions were met: 1) there were multiple consecutive current intensities at six stimulus intensities from 100 to 1,000 μA, in which the stimulus was imperceptible and the velocity improved compared to the baseline, 2) at the current intensity at which the velocity improved the most, the velocity improved by more than 10% compared to the baseline. Patients who had an optimal intensity were enrolled in the main study. The optimal intensity used for this enrolment was the current that improved velocity the most among the multiple current intensities described above. Since the safety of prolonged stimulation with high current for 4 hours is questionable, the optimal intensity in the main clinical trial was set at a value within 1000 μA.

### Randomization and masking

All persons involved in the clinical trial (except those who performed the randomization assignment work for the investigational device) were blinded to the randomization information. The subjects enrolled in the main study were assigned to two groups (Group A or Group B) in a 1:1 ratio by block randomization using the web-based randomization system (Datatrak One) with a block size of 4. The randomization was stratified by vestibulopathy (unilateral vestibulopathy, bilateral vestibulopathy) and TTL in the screening period (≥ 200 cm, < 200 cm). In Group A, the effect of the optimal intensity of nGVS on body balance was evaluated in "Session I" and the placebo effect (0 μA) was evaluated 14 days later (at least seven days later) in "Session II". In Group B, the placebo effect on body balance was evaluated in "Session I" and the effect of the optimal intensity of nGVS was evaluated 14 days later (at least seven days later) in "Session II".

For Session I and Session II, the results of randomization of each subject in the registration system were confirmed by the allocation manager other than the investigator (or sub-investigator) at the medical institution. The investigator informed the allocation manager of the optimal intensity measured in the screening period, and the allocation manager set the device to either the placebo stimulus or the optimal intensity according to the allocation result. Neither the subject nor the investigator was able to distinguish the placebo stimulus from the optimal intensity. Before each session, the investigator or sub-investigator gave the subject the set stimulus for 30 s and asked whether the subject perceived the stimulus. If the stimulus was below the sensory threshold, the stimulus of that intensity was used for the prolonged stimulation. If the stimulus was above the sensory threshold but the subject did not feel any discomfort, the stimulus of that intensity was also used for the prolonged stimulation. However, in this case, the subject was excluded from the main analysis and only used for the supplementary analysis. At each measurement time point during the stimulation in each session, the subject was asked if he/she could perceive the stimulus. If the subject became able to perceive the stimulus, the data up to the time point before the stimulus became perceptible were included in the main analysis, but the data obtained after the stimulus was found to be above the sensory threshold were used only for the supplementary analysis. The allocation director and the allocation manager kept the randomization allocation information confidential until the time of key opening.

In addition, even during placebo stimulation, the nGVS portable stimulator was set to blink light-emitting diodes in the same way as seen during actual nGVS, so that the investigators and subjects had no way of distinguishing between the optimal and the placebo stimulation.

### Procedures

**Fig 1:**
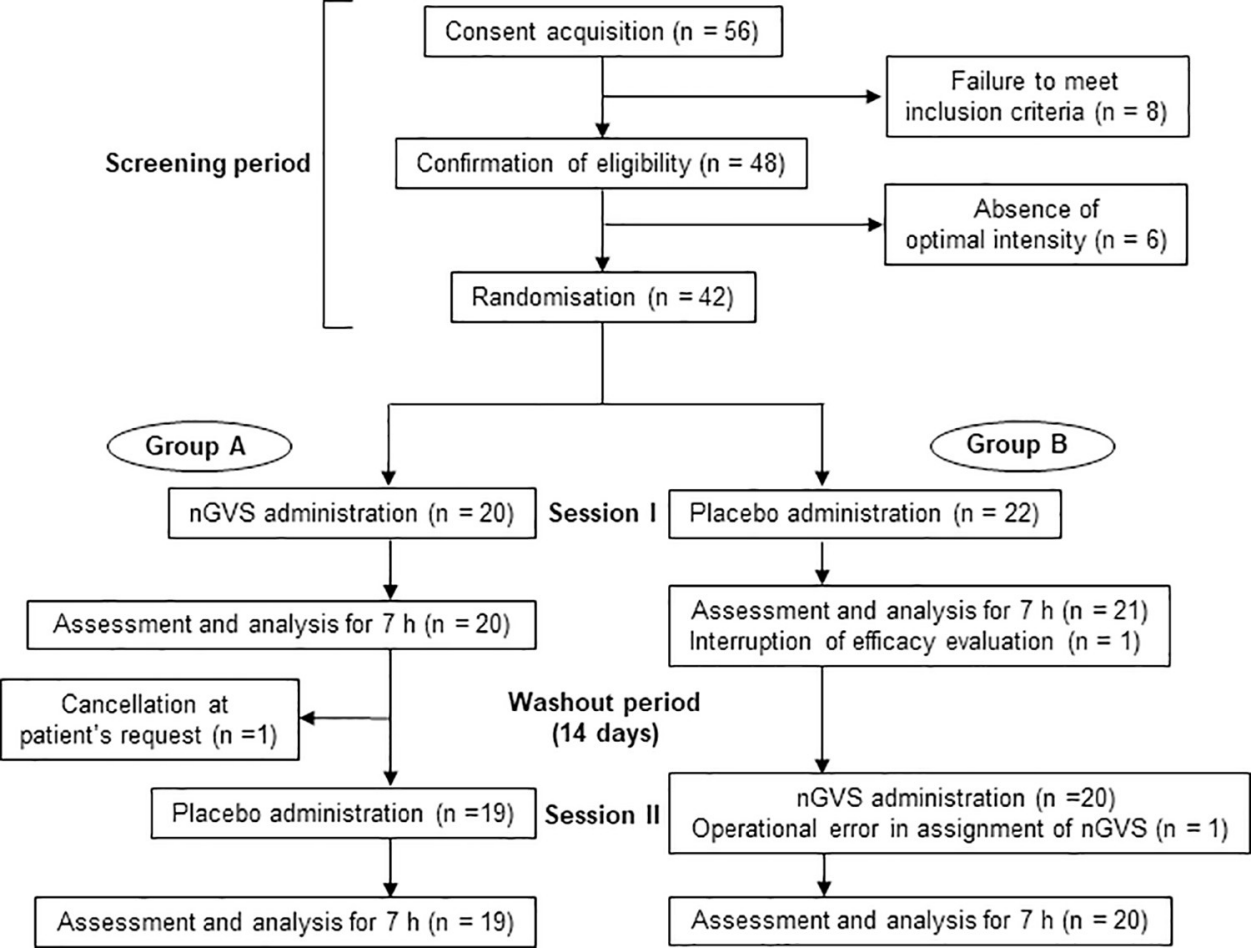
Trial profile. In Group A, the effect of the optimal intensity of nGVS on body balance was evaluated in "Session I" and the placebo effect (0 μA) was evaluated 14 days later in "Session II". In Group B, the placebo effect on body balance was evaluated in "Session I" and the effect of the optimal intensity of nGVS was evaluated 14 days later in "Session II". nGVS = noisy galvanic vestibular stimulation.

The subjects whose written informed consent was obtained and whose eligibility was confirmed by meeting the inclusion and exclusion criteria were provisionally enrolled (**Fig 1**). Subsequently, only subjects for whom an optimal intensity existed were enrolled in the main study. The subjects without an optimal intensity were excluded from the trial.

The subjects enrolled in the main study visited the hospital 14 days (at least seven days) after enrolment and were randomly assigned to either Group A or Group B in a 1:1 ratio, according to the randomization scheme described above. For Session I and Session II, subjects were stimulated with the optimal intensity of nGVS or placebo stimulation for 4 h and were subsequently observed for 3 h after the end of stimulation. During each session, the subjects were instructed to stay within the hospital where the clinical trial was conducted, although they were not instructed to do anything specific other than the examinations prescribed in the present study. The washout period between each session was 14 days (at least seven days). In Group A, Session I was nGVS and Session II was placebo. The order of stimulation methods in Group B was the reverse of Group A. Fourteen days after the end of Session II, the subjects were interviewed for adverse events.

Posturography with the eyes closed was performed immediately before stimulation, immediately after the start of stimulation, 0.5 h later, and every hour from 1 h to 7 h later. The average of the three measurements at 2-min intervals immediately before stimulation was used as the baseline measurement. Gait analyses using IMUs and the DGI short form were performed immediately before stimulation, immediately after the start of stimulation, and every hour from 1 h to 7 h later [26,27] The subjects themselves were asked to evaluate their balance disorder on a 5-point scale of: 1 (improved), 2 (slightly improved), 3 (unchanged), 4 (slightly worse), 5 (worse) (subjective improvement score), immediately after the start of stimulation, and every hour from 1 h to 7 h later, compared to their state before stimulation. The present study required not only the posturography test, but also the gait analysis and the questionnaire survey. It was determined that the posturography test could not be measured more than once at each measurement time point after the start of stimulation due to time constraints. Therefore, only one measurement of the posturography test was performed at each measurement time point.

For safety, all the adverse events that occurred during the use of the stimulator were recorded.

We considered that the accuracy of endpoints could be improved by using a cross-over design, which collects data under both stimulation conditions for each patient. The number of patients with vestibulopathy and a severe balance disorder that would qualify for this study was likely to be very limited, and the procedures for assessing postural and gait function were complicated. Therefore, this study was designed as a crossover study to ensure the maximum accuracy of results given the limited number of centers and patients. Regarding the washout period, it was shown in our previous feasibility study that if nGVS was performed after a 14-day interval, there would be no residual effect on any subsequent measurement of posturography [21]. Although a 14-day washout period was used in this trial, a minimum washout period of seven days is considered as acceptable in order to minimize deviations from the protocol due to subject convenience, and because the results of an examination of the post-stimulation effect after the end of nGVS showed no carryover effect even 4 h post-stimulation. Therefore, a minimum washout period of seven days was set as acceptable.

### Outcomes

The primary outcome was the percent change from the baseline in the velocity of COP at all measurement time points from the start of stimulation to 3 h (stimulation ended at 4 h). The difference between this mean percent change in the nGVS period and that in the placebo period was compared.

Secondary endpoints were as follows: 1) the percent change from the baseline in the area and RMS at all measurement time points from the start of stimulation to 3 h, 2) the percent change from the baseline in the velocity, area, and RMS at all the measurement time points from 4 h to 7 h after the start of stimulation, 3) the percent change from the baseline in DGI short form scores at all the measurement time points from the start of stimulation to 7 h, 4) the percent change from the baseline in the gait speed, stride length, stride duration and lateral movement distance (trunk) at all the measurement time points from the start of stimulation to 7 h, 5) the subjective improvement score at all the measurement time points from the start of stimulation to 7 h, 6) the number of steps measured by pedometer from the start of stimulation to 3 h and from the start of stimulation to 7 h.

### Statistical analysis

We aimed to recruit 60 subjects, and among them, we expected 40 eligible subjects would complete the study. Briefly, we considered that a 10% difference in the percent change from the baseline in the velocity between the nGVS period and the placebo period was clinically meaningful, referring to the difference in the velocity of patients with vestibulopathy and that of healthy controls [29]. A computer simulation using the means and (co)variances observed in the previous study [21] revealed that a sample size of 40 subjects would have more than 90% power to detect a clinically relevant difference with a type I error rate of 5% in a two-sided test. We set a target of 50 subjects to be enrolled in the study, given that 20% of the subjects enrolled in the study would drop out. We also predicted that approximately 15% of the recruited subjects would not enroll in the study due to lack of optimal intensity. Thus, we expected to recruit 60 subjects, of which 50 would enroll and 40 eligible subjects would complete the study.

We presented efficacy endpoints by their means and standard errors in the nGVS and placebo periods. The primary endpoint was analyzed using the mixed effects model. The analysis included the fixed categorical effects of periods, treatments (nGVS or placebo), time points, time-by-treatments interaction, and two randomization factors. Between-subjects variance was modelled using a random intercept. The Kenward-Roger approximation was used to estimate denominator degrees of freedom. The significance test was based on the least-squares means of the difference between the nGVS period and placebo period from the start of stimulation to 3 h using a two-sided α = .05 (two-sided 95% confidence intervals). Other standing postural stability endpoints, gait performance endpoints, and subjective improvement were analyzed using similar models. Multiple comparison correction was not used for secondary outcomes. This is because we did not set the key secondary outcome a priori. For the subject who felt the nGVS, data after the measurement time point when the subject felt the nGVS were considered missing, but data from the other time points were used in the analysis. In the mixed effects model, the least-squares means for each time point were estimated by using the missing at random assumption to estimate the missing data from the non-missing data. Prespecified subgroup analyses were conducted for BVP/UVP and TTL (≥ 200cm, < 200cm) in the screening period. We analyzed efficacy data for the full analysis set (FAS), which includes all patients who experienced at least one nGVS or placebo stimulation at Session I or II and who have at least one efficacy endpoint. These patients were analyzed according to the randomized groups, even some of them discontinued stimulation (intention-to-treat principle). We analyzed safety data for the safety analysis set (SAS), which includes all patients who experienced at least one nGVS or placebo stimulation at Session I or II. All statistical analyses and sample size calculations were done with SAS (version 9.4; SAS Institute Inc., Cary, NC, USA).

## Results

Between 24 January 2019 and 6 May 2022, 48 patients were screened, out of which six patients did not have an optimal intensity (**Fig 1**). Forty-two patients were enrolled of which 26 were UVP and 16 were BVP. The velocity of the movement of the COP at the applied nGVS current intensity for each enrolled subject when measuring optimal intensity is shown in **S1 Fig in S3 File.** The percent change from the baseline in the velocity at applied nGVS current intensity for each enrolled subject when measuring optimal intensity is shown in **S2 Fig in S3 File**. The clinical diagnoses for the 42 enrolled patients are shown in **S1 Table in S5 File**. Twenty patients were assigned to Group A (six female, seven BVP) and 22 to Group B (eight female, nine BVP) in the FAS and the SAS. Mean age was 68.1 years (SD 10.4) (**Table 1**). Of the 42 subjects, one patient withdrew from the study at her own request; one patient complained of feeling nGVS at two measurement time points (2 and 3 h in Session I), but after the key opening, it was determined that this patient had felt stimulation during the placebo period. One patient received placebo stimulation in both periods due to an operation error. No subjects experienced a fall during posturography testing. Velocity, area, and RMS for each enrolled subject during the nGVS and placebo periods are shown in **S3-S8 Fig in S3 File**. The percent change from the baseline in velocity, area, and RMS for each enrolled subject during the nGVS and placebo periods are shown in **S9-S14 Fig in S3 File**. Efficacy analyses were performed on the FAS.

**Table 1:** Demographics and baseline characteristics.

	Total (N = 42)	Group A (N = 20)	Group B (N = 22)
Age, years			
Mean (SD)	68.1 (10.4)	72.0 (10.4)	64.6 (9.2)
Median (IQR)	70.0 (61.0–77.0)	74.5 (68.0–79.5)	66.0 (56.0–71.0)
Height, cm			
Mean (SD)	163.52 (8.39)	164.43 (6.89)	162.69 (9.64)
Median (IQR)	160.00 (164.00–168.70)	160.05 (164.30–168.80)	160.00 (163.70–166.20)
Weight, kg			
Mean (SD)	66.23 (10.87)	68.79 (9.91)	63.90 (11.40)
Median (IQR)	68.40 (58.70–74.80)	69.95 (61.40–76.75)	62.9 (58.5–72.7)
Body mass index, kg/m^2^			
Mean (SD)	24.67 (3.11)	25.4 (3.1)	24.0 (3.04)
Median (IQR)	24.28 (22.57–26.42)	24.78 (23.00–27.83)	23.85 (22.29–25.97)
TTL, cm			
Mean (SD)	329.03 (114.16)	325.31 (114.21)	332.42 (116.69)
Median (IQR)	307.76 (227.93–410.74)	309.34(225.55–399.57)	307.76(231.69–451.86)
Optimal intensity, μA			
Mean (SD)	481.0 (266.2)	495.0 (303.4)	468.2 (233.8)
Median (IQR)	500.0 (300.0–700.0)	500 (200.0–700.0)	500 (300.0–500.0)
Sex, N			
Female (%)	14 (33.3)	6 (30.0)	8 (36.4)
Male (%)	28 (66.7)	14 (70.0)	14 (63.6)
Presence of sensory thresholds, N			
Yes (%)	30 (71.4)	15 (75.0)	15 (68.2)
No (%)	12 (28.6)	5 (25.0)	7 (31.8)
TTL at screening period, N *			
≥ 200 cm (%)	39 (92.9)	19 (95.0)	20 (90.9)
< 200 cm (%)	3 (7.1)	1 (5.0)	2 (9.1)
BVP or UVP, N *			
BVP (%)	16 (38.1)	7 (35.0)	9 (40.9)
UVP (%)	26 (61.9)	13 (65.0)	13 (59.1)

N = number, SD = standard deviation, IQR = interquartile range, TTL = total trajectory length, BVP = bilateral vestibulopathy, UVP = unilateral vestibulopathy, * = the stratification factors.

### Primary outcome

**Fig 2:**
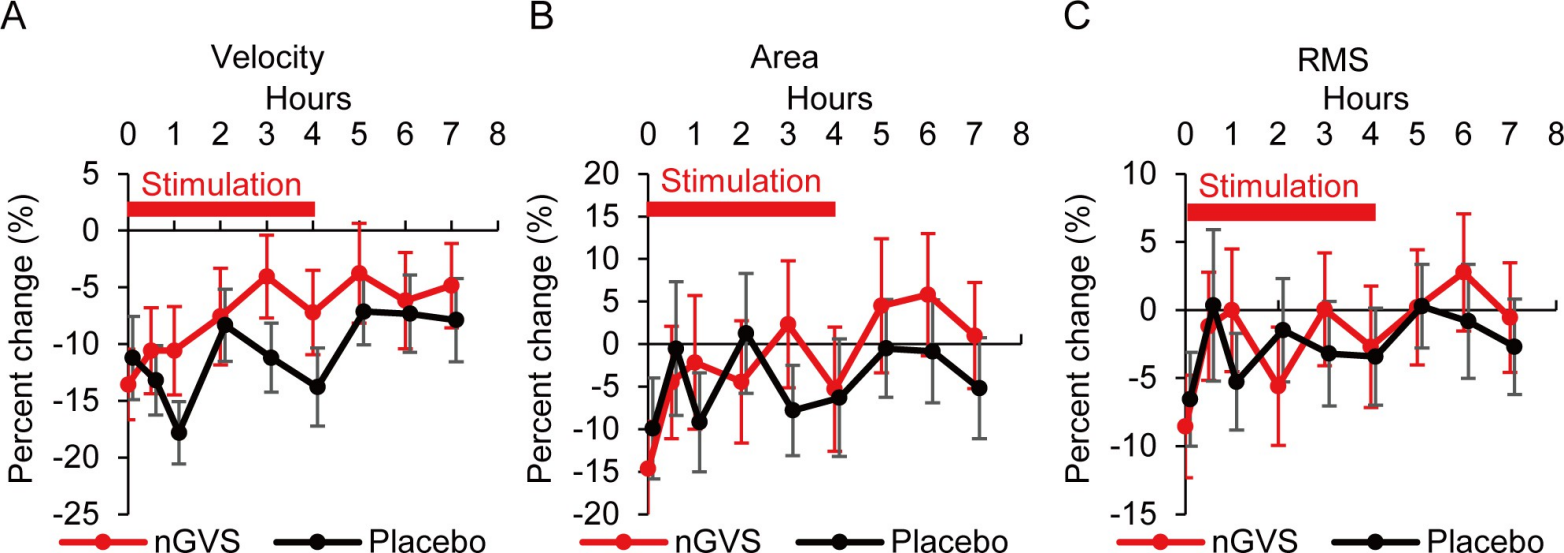
The percent change from the baseline in velocity (A), area (B), and RMS (C) during the nGVS and placebo periods. The solid red line shows the percent change from baseline during the nGVS period. The solid black line shows the percent change from baseline during the placebo period. The label "Hours" on the horizontal axis indicates hours from the start of stimulation. 0 h on the horizontal axis refers to the time immediately after the start of stimulation. The number of cases in the nGVS period was 40. The number of cases in the placebo period was 41 at 0 h, 0.5 h, and 1 h after the start of stimulation, and 40 at 2 h, 3 h, 4 h, 5 h, 6 h, and 7 h after the start of stimulation. RMS = root mean square. nGVS = noisy galvanic vestibular stimulation.

The effects of nGVS on standing postural stability were analyzed using posturography. **Fig 2** shows the percent change from the baseline in the mean velocity (velocity), the envelopment area (area), and the RMS of the movement of the COP during the nGVS and placebo periods, in which a lower percent change from baseline indicates a greater degree of improvement. The mixed-effect model analysis showed that the mean percent change in the velocity from the start of stimulation to 3 h, the primary endpoint of this trial, was -9.4% for nGVS and -12.5% for placebo in patients who achieved both Session I and II (N = 39), while crude analysis showed that -9.3% (SD 19.9%) for nGVS and -12.6% (SD 15.0%) for placebo. No significant effect of nGVS over placebo was found on the velocity of COP in the least-squares means of the difference in the mixed effects model for FAS (N = 42) [3.1% (95% CI -0.2 to 6.4, p = 0.066), **Table 2**].

**Table 2:** Mixed-effects models to compare mean percent change from baseline in velocity, area and RMS from the start of stimulation to 3 h between nGVS and placebo periods.

	Period	Least-squares mean of mean percent change from baseline	
		Estimate (95% CI) (%)	p value
Velocity (Main analysis)	nGVS	-2.740 (-12.182 to 6.702)	
	placebo	-5.834 (-15.260 to 3.592)	
	nGVS—placebo	3.094 (-0.212 to 6.400)	0.066
Area	nGVS	4.340 (-12.440 to 21.119)	
	placebo	3.677 (-13.065 to 20.419)	
	nGVS—placebo	0.662 (-6.167 to 7.491)	0.849
RMS	nGVS	4.604 (-5.426 to 14.633)	
	placebo	4.307 (-5.699 to 14.313)	
	nGVS—placebo	0.297 (-3.929 to 4.523)	0.890

RMS = root mean square, nGVS = noisy galvanic vestibular stimulation, CI = confidence interval.

### Secondary outcomes

Multiple comparison correction was not used for secondary outcomes.

For area and RMS, analysis of the mixed effects model showed no significant differences in the mean percent change between nGVS and placebo from the start of stimulation to 3 h [0.7%, 95% CI -6.2 to 7.5, p = 0.849 (for area); 0.3%, 95% CI -3.9 to 4.5, p = 0.890 (for RMS)] (**Table 2**). Analysis of intraindividual differences between nGVS and placebo at each measurement time point showed that the mean percent change in velocity for placebo was significantly higher than that for nGVS at 1 h and 3 h after the start of stimulation, while the mean percent change in area and RMS were not significantly different between these two periods (**S2 Table in S5 File**). The mean percent changes of nGVS and placebo in each session are shown in **S3 Table in S5 File**.

Subgroup analyses of the effect of nGVS on standing postural stability were conducted for BVP/UVP and TTL in the screening period (≥ 200 cm, < 200 cm). For BVP patients (N = 16), no significant effect of nGVS over placebo was found on the mean percent change in velocity from the start of stimulation to 3 h [0.3%, 95% CI -5.8 to 6.4, p = 0.920] (**S4 Table in S5 File**). For UVP patients (N = 26), the mean percent change in velocity for placebo was significantly higher than that for nGVS from the start of stimulation to 3 h [4.6%, 95% CI -0.8 to 8.4, p = 0.019]. For both BVP and UVP with TTL ≥ 200 cm (N = 39) and those with TTL < 200 cm (N = 3) in the screening period, no significant effect of nGVS over placebo was found on the mean percent change in velocity from the start of stimulation to 3 h [2.5%, 95% CI -0.9 to 6.0, p = 0.152 (for TTL ≥ 200 cm); 11.6%, 95% CI -0.2 to 23.4, p = 0.053 (for TTL < 200 cm)] (**S5 Table in S5 File**).

The effects of nGVS on gait performance were analyzed. No significant effect of nGVS over placebo was found in the mean percent change in gait speed, stride length, stride duration, lateral movement distance (trunk), or DGI score from the start of stimulation to 3 h [0.7%, 95% CI -0.8 to 2.1, p = 0.375 (for gait speed); 0.6%, 95% CI -0.5 to 1.7, p = 0.278 (for stride length); 0.2%, 95% CI -0.6 to 0.9, p = 0.686 (for stride duration); 1.1%, 95% CI -2.6 to 4.8, p = 0.562 (for lateral movement distance (trunk)); -2.6%, 95% CI -21.1 to 15.9, p = 0.785 (for DGI score)] (**Table 3**). Analysis of intraindividual differences between the two periods at each measurement time point showed no significant difference in the mean percent change in gait speed, stride length, stride duration, lateral movement distance (trunk), or DGI score (**S6 Table in S5 File**).

**Table 3:** Mixed-effects models to compare mean percent change from baseline in gait speed, stride length, stride duration, lateral movement distance (trunk) and DGI score from the start of stimulation to 3 h between nGVS and placebo periods.

	Period	Least-squares mean of mean percent change from baseline	
		Estimate (95% CI) (%)	p value
Gait speed	nGVS	4.959 (1.039 to 8.879)	
	placebo	4.291 (0.379 to 8.203)	
	nGVS—placebo	0.668 (-0.811 to 2.148)	0.375
Stride length	nGVS	2.148 (0.696 to 5.582)	
	placebo	2.544 (0.107 to 4.981)	
	nGVS—placebo	0.595 (-0.482 to 1.673)	0.278
Stride duration	nGVS	-1.432 (-3.311 to 0.446)	
	placebo	-1.589 (-3.463 to 0.286)	
	nGVS—placebo	0.156 (-0.604 to 0.916)	0.686
Lateral movement distance (trunk)	nGVS	5.726 (-1.966 to 13.418)	
	placebo	4.622 (-3.048 to 12.293)	
	nGVS—placebo	1.103 (-2.635 to 4.842)	0.562
DGI score	nGVS	22.046 (-16.346 to 60.438)	
	placebo	24.611 (-13.657 to 62.880)	
	nGVS—placebo	-2.565 (-21.057 to 15.927)	0.785

nGVS = noisy galvanic vestibular stimulation, CI = confidence interval, DGI = dynamic gait index.

The subjective improvement score from the start of stimulation to 3 h during the placebo period was significantly higher than that in the nGVS period [p = 0.028] (**Table 4**), although no significant intraindividual difference was found between the two periods at each measurement time point (**S7 Table in S5 File**). In both the nGVS and placebo periods, the correlation coefficient between the number of steps taken from the start of stimulation and the percent change from baseline in velocity was low (**S8 Table in S5 File**).

**Table 4:** Mixed-effects models to compare subjective improvement score from the start of stimulation to 3 h between nGVS and placebo periods.

		Least-squares mean of subjective improvement score	
Estimate (95% CI)	p value
Period	nGVS	2.999 (2.732 to 3.267)	
	placebo	2.880 (2.613 to 3.147)	
	nGVS—placebo	0.119 (0.013 to 0.225)	0.028*

nGVS = noisy galvanic vestibular stimulation, *p < 0.05.

In the safety assessment of nGVS, six adverse events occurred during the nGVS period (**S9 Table in S5 File**). However, none had a clear causal association to nGVS.

## Discussion

This trial revealed that prolonged nGVS for 4 h did not have ameliorating effects on posture, gait, or subjective symptoms compared to placebo stimulation in patients with vestibulopathy.

This trial included UVP patients as well as BVP patients. Subgroup analysis of UVP and BVP patients separately showed no improvement in posture or gait ability in either patient group. Therefore, whether the vestibular dysfunction was bilateral or unilateral had no effect on the results in this trial.

While improvement in subjective scores was rather greater in the placebo period than the nGVS period, the mean improvement score in the placebo period (2.880, **Table 4**) was not substantial. Therefore, we considered that both the nGVS and the placebo had little effect on participants’ subjective scores.

In the safety assessment of nGVS, six adverse events occurred in the nGVS period, and five of them were judged to be unrelated to nGVS. The remaining one was a mild headache, which could not be ruled out as related to nGVS. However, this headache symptom subsequently resolved. Therefore, none of the adverse events had a clear causal association to nGVS.

This is the multicenter, randomized, double-blind, placebo-controlled trial of the effect of prolonged nGVS on body balance in patients with vestibulopathy. To date, previous small intervention trials exploring the effect of nGVS on body balance in patients with vestibulopathy have shown conflicting results [4,14,16–18,22]. These previous studies have not adequately addressed the placebo effect of nGVS. Therefore, this trial is important in determining whether nGVS is effective in treating balance disorders in patients with vestibulopathy.

In the present study, the post-stimulation measurement time points were set at 5 h, 6 h, and 7 h after the start of stimulation. nGVS and placebo periods did not differ significantly in their effect on balance improvement at the post-stimulation measurement time points (**S2 and S6 Tables in S5 File**). Our group has previously reported that nGVS improved standing postural control for several hours after cessation of the stimulus in both healthy elderly and BVP patients [3,19,21]. However, in these feasibility studies, no comparison was made between nGVS and no stimulation. In the present study, there was approximately a 5% improvement in velocity from baseline in the nGVS period at the post-stimulation measurement time points, but the placebo period showed more improvement than the nGVS period (**Fig 2**). Therefore, the placebo effect is likely to be high at 5 h, 6 h, and 7 h after the start of stimulation.

With regard to the intensity of nGVS, the present study, as well as our previous feasibility studies, used an optimal intensity, which improved the subject’s body balance the most, determined on a subject-by-subject basis. In our previous feasibility studies, the examination of the effects of prolonged nGVS was conducted on the same day that the optimal intensity was determined [3,21]. However, in this trial, to avoid the carryover effect of nGVS used to determine the optimal intensity, the day on which the optimal intensity was determined was two to four weeks before the day on which prolonged nGVS was applied. Since the optimal intensity varies from day to day [30], the current intensity used on the day of prolonged stimulation might not have been optimal that day.

In this trial, nGVS was administered for 4 h to examine the effects of nGVS on body balance during prolonged stimulation. However, in our previous feasibility study on BVP patients, the ameliorating effects of nGVS after the cessation of the stimulus were observed even when nGVS was administered for only 30 min [21]. In another feasibility study by our group, the ameliorating effects during a 3-hour stimulation tended to be poorer than those after the cessation of the stimulus [3]. Further clinical studies are needed to determine the optimal duration of nGVS on balance improvement.

The quality of movement may deteriorate with the onset of fatigue in elderly subjects. The subjects were instructed to stay within the hospital where the clinical trial was conducted, although they were not instructed to do anything specific other than the examinations prescribed in the present study. In the present study, the number of steps was counted with the intention of comparing activity between the nGVS period and the placebo period, but there were no significant differences between the two groups.

There are several limitations to this study. First, the current intensity used on the day of prolonged stimulation may not have been optimal if the optimal intensity varies from day to day. Second, there is a lack of detailed exploration of the stimulation methods by which nGVS can effectively affect balance, particularly using shorter durations and repetitive stimulation. Third, we did not control for potential learning effects of repeated measure of posturography test in the procedure to identify the optimal nGVS intensity. Fourth, we did not evaluate parameters that quantify the variability of gait coordination such as the coefficient of variation of stride duration or stride length. We also did not evaluate parameters related to the asymmetry of the postural COP displacement. The selected parameters may potentially be unspecific for expected treatment effects.

## Conclusions

In conclusion, this study found no ameliorating effects of prolonged nGVS on body balance compared to placebo in patients with vestibulopathy.

## Supporting information

S1 File CONSORT checklist. (DOCX)

S2 File Original study protocol. (DOCX)

S3 File Supplementary Figures S1-S14. (DOCX)

S4 File Study protocol English translation. (DOCX)

S5 File Supplementary Tables S1-S9. (DOCX)

S6 File Anonymized data. (CSV)
