# Supplementary material for: Multicenter randomized double-blind placebo-controlled crossover study of the effect of prolonged noisy galvanic vestibular stimulation on posture or gait in vestibulopathy
Source: PLoS One. 2025 Jan 24;20(1):e0317822. doi: 10.1371/journal.pone.0317822 (PMC11760040; doi:10.1371/journal.pone.0317822)
Supplement: S2 File — (DOCX) [file pone.0317822.s002.docx]

重度のふらつきを有する難治性前庭障害患者における経皮的ノイズ前庭電気刺激によるバランス障害改善効果と安全性を確認するための二重盲検ランダム化プラセボ対照クロスオーバー試験
治験実施計画書

**治験実施計画書番号：GVS001**

**版数：Ver 16.0（2022年6月1日作成）**

**自ら治験を実施する者　治験調整医師**

**岩﨑 真一（名古屋市立大学医学部　耳鼻咽喉・頭頸部外科　教授）**

# 治験実施計画書の要約

区分：医師主導治験

治験機器名・記号：　携帯型前庭電流刺激装置　・GVS-100

開発段階（相）：検証的試験（第Ⅲ相）

作成日：2022年6月1日

版数　16.0

標題：

重度のふらつきを有する難治性前庭障害患者における経皮的ノイズ前庭電気刺激によるバランス障害改善効果と安全性を確認するための二重盲検ランダム化プラセボ対照クロスオーバー試験

自ら治験を実施する者（治験調整医師）：

岩﨑 真一

名古屋市立大学医学部　耳鼻咽喉・頭頸部外科　教授

愛知県名古屋市瑞穂区瑞穂町字川澄 1 番地

Tel: 052-851-5511（代表）

Mail: iwashin-tky@umin.ac.jp

治験実施施設：

本治験は東京大学医学部附属病院（耳鼻咽喉科）、東京逓信病院（耳鼻咽喉科）、名古屋市立大学病院（耳鼻いんこう科）において実施する。

治験実施期間：

2018年12月から2022年6月まで

（最初の被験者の同意取得から最後の被験者の治験終了まで）

目的：

本治験の主要目的は、前庭機能障害に基づく重度のふらつき症状を有する患者を対象に、携帯型前庭電流刺激装置による経皮的ノイズ前庭電気刺激（ノイズGVS［Galvanic Vestibular Stimulation］）を4時間加え、刺激中の体平衡機能改善効果について、プラセボ刺激との比較検討を行うことである。また副次目的は、ノイズGVSの4時間刺激後の有効性及び刺激中・刺激後の安全性を検討することである。

治験デザイン：

本治験は、多施設共同、二重盲検、ランダム化、プラセボ対照、クロスオーバー試験である。

図1に示す通り、文書同意が得られ、スクリーニングで適格性が確認された被験者を仮登録する。その後、最適刺激の検討において、最適刺激が存在した被験者のみ本登録を行う。最適刺激が存在しない被験者はこの時点で中止し、2週後の安全性を確認し終了となる。

本登録された被験者は、本登録後およそ2週間後に来院し、『第Ⅰ期』に最適刺激のノイズGVSによる体平衡機能改善の効果を評価した後に、『第Ⅱ期』にプラセボ刺激による評価を行う群（A群）と、その逆の順序で『第Ⅰ期』にプラセボ刺激による体平衡機能改善の効果を評価した後に、『第Ⅱ期』に最適刺激のノイズGVSによる評価を行う群（B群）の2群に1：1の比率でランダム割り付けされる。なお、本登録から第Ⅰ期まで、最低7日間の期間を空けることとする。

各被験者に、携帯型前庭電流刺激装置による刺激（「被験者毎の最適刺激」又は「プラセボ刺激」）を4時間加え、刺激前及び刺激中におけるふらつきと安全性を測定する。さらに、刺激後3時間についても安全性と有効性を評価することとし、全ての有害事象及び不具合の確認を行う。なお、第Ⅰ期の評価の後、第Ⅱ期の評価までに14日程度、最低7日間の期間を空けることとする。治療刺激期間の『第Ⅱ期』の評価終了2週間後に有害事象の有無についての聞き取り調査を行う。

なお、安全性については、刺激装置の使用時に生じた全ての有害事象及び不具合の確認を行う。

**スクリーニング**

**同**

**意**

**取**

**得**

**適格性確認**

**仮登録**

**最適刺激の検討**

2週後

**本登録**

**有**

**第Ⅰ期**

**第Ⅱ期**

**２週間の**

**ウォッシュアウト**

**GVS**

**４時間刺激**

**Placebo**

**4時間**

**刺激**

**事後**

**観察**

**3時間**

**事後**

**観察**

**3時間**

**刺激**

**Placebo**

**4時間**

**刺激**

**事後観察**

**3時間**

**GVS**

**4時間**

**刺激**

**事後**

**観察**

**3時間**

**刺激**

A群

**追跡調査**

**安全性の確認**

**終了**

**無**

B群

2週後

**二重盲検**

**二重盲検**

6週間

2週後

図1　治験のフローチャート

被験者数：

60例（本登録50例、完了例40例）（A群：25例、B群：25例）

治験の対象集団：

以下の選択基準を満たし、除外基準に抵触しない前庭機能障害に伴う重度のふらつきを有する患者を対象とする。

- 選択基準

1. 氷水2 mLの温度刺激検査にて、一側あるいは両側の前庭障害を有する患者

一側障害はCP（Canal Paresis，半規管麻痺）(%)=(健側の最大緩徐相速度 - 患側の最大緩徐相速度)/(健側の最大緩徐相速度＋患側の最大緩徐相速度) x100 ≧ 20%、両側障害は眼振が誘発されないか、両側の最大緩徐相速度が10度/秒以下の末梢前庭障害患者とする。なお、CPの算出において、最大緩徐相速度の代わりに、眼振の持続時間を用いることも可とする。

1. 閉眼起立時の重心動揺計において、60秒間で総軌跡長が180cm以上の患者。
2. ふらつきを発症後1年以上経過し、リハビリテーション治療を行ってもふらつきの症状が少なくとも6か月以上持続する患者。
3. 年齢20歳以上85歳未満の前庭障害に伴う重度の患者とする。性別、難聴の有無は問わない。
4. 本治験の内容を理解し、本治験の参加前に自らの自由意思で文書同意した患者

- 除外基準

1. 脳動脈クリップ、人工内耳やペースメーカーなど体内に金属を有する患者（銀歯は可）
2. 整形外科的疾患を有する患者（例として骨折、捻挫、肉離れ等、急性疼痛性疾患のある患者等）
3. 小脳障害や脊髄疾患による四肢の運動障害を有する患者
4. ペースメーカーが必要となりうる重度の不整脈（心房細動、重度のQT延長症候群、重度の房室ブロック（II度以上））、歩行に支障をきたすような重症の心不全など、重大な心疾患を有する患者
5. 悪性腫瘍を有する患者
6. 発熱、倦怠感又はふらつき等を伴う感染症疾患を有する患者
7. 妊娠中、出産直後の患者
8. 自力歩行が出来ない患者
9. 装着部位の皮膚に異常（感染症、創傷など）のある患者、又はアナフィラキシーショックの既往がある等、重度のアレルギー体質のある患者
10. 法的能力の欠如又は制限のある患者
11. 同意取得日より前3ヶ月以内に他の治験（臨床試験）に参加した者、又は本治験と同時期に他の治験（臨床試験）に参加する患者
12. その他、治験責任（分担）医師が被験者として不適当と判断した患者

- 本登録基準

ノイズGVSにて最適刺激が存在する患者

治験の評価項目と評価：

主要評価項目：

重心動揺計（アニマ社製、Gravicorder G-620型）を用いて測定した、治験機器によるノイズGVS開始直後、30分後、1時間後、2時間後、3時間後の全時点における総軌跡長のベースラインからの変化率

副次評価項目：

<有効性評価>

- ノイズGVS開始直後、30分後、1時間後、2時間後、3時間後の全時点における外周面積、RMS（ Root Mean Square［全重心動揺の平均位置と計測された重心位置の距離の二乗平均の平方根］）値のベースラインからの変化率
- ノイズGVS開始4時間後、5時間後、6時間後、7時間後の全時点における総軌跡長、外周面積、RMS値のベースラインからの変化率
- 歩行機能

ノイズGVS開始直後、1時間後、2時間後、3時間後、4時間後、5時間後、6時間後、7時間後における以下の項目のベースラインからの変化率

- Dynamic Gait Index（DGI Short version）のスコア
- 歩行分析計（WALK-MATE LAB株式会社、Walk Mate Viewer）を用いて測定する10メートル歩行時の歩行速度、歩幅、ステップ時間及び左右の揺れ幅
- 自覚的改善度スコア

ノイズGVS開始直後、1時間後、2時間後、3時間後、4時間後、5時間後、6時間後、7時間後における以下の項目

- - 被験者自身が刺激前の状態と比較して「1：改善、2：やや改善、3：不変、4：やや悪化、5：悪化」の5段階での評価
- Quality of Life (QOL)

ノイズGVS開始3時間後及び7時間後における以下の項目のベースラインからの変化量

- - 日本語版modified Fall Efficacy Scale（mFES）（別添2）
  - 日本語版Dizziness Handicap Inventory（DHI）（別添3）
- 活動量

ノイズGVS開始直後から3時間後、及び7時間後までの活動量（歩行数）

<安全性評価>

有害事象及び不具合の発生を評価する。

# 目次

ページ

[1. 治験実施計画書の要約 1](#_Toc33784534)

[目次 5](#_Toc33784535)

[2. 略号及び用語の定義 9](#_Toc33784536)

[3. 緒言 9](#_Toc33784537)

[3.1. 背景・経緯 9](#_Toc33784538)

[3.2. 非臨床試験の要約 11](#_Toc33784539)

[3.2.1. 機械的及び電気的試験 11](#_Toc33784540)

[3.2.2. 生物学的安全性評価 11](#_Toc33784541)

[3.3. 臨床試験の要約 12](#_Toc33784542)

[3.3.1. 自主臨床試験1及び2の成績 12](#_Toc33784543)

[3.3.2. 安全性 12](#_Toc33784544)

[3.4. 自主臨床試験3の成績^8)^ 13](#_Toc33784545)

[3.4.1. 経皮的ノイズGVS終了後の持ち越し効果 13](#_Toc33784546)

[3.4.2. 最適刺激強度の変動の有無 14](#_Toc33784547)

[3.4.3. 安全性 14](#_Toc33784548)

[3.5. 治験の位置付け 15](#_Toc33784549)

[4. 目的 15](#_Toc33784550)

[5. 治験の評価項目 16](#_Toc33784551)

[5.1. 主要評価項目 16](#_Toc33784552)

[5.2. 副次評価項目 16](#_Toc33784553)

[6. 治験の計画 17](#_Toc33784554)

[6.1. 治験デザイン 17](#_Toc33784555)

[6.1.1. スクリーニング期間 17](#_Toc33784556)

[6.1.2. 最適刺激の検討 17](#_Toc33784557)

[6.1.3. 治療刺激期間 17](#_Toc33784558)

[6.1.4. 安全性の追跡調査期間 18](#_Toc33784559)

[6.2. 治験デザインの考察 18](#_Toc33784560)

[7. 被験者の選択と中止基準 18](#_Toc33784561)

[7.1. 被験者の組み入れ基準 18](#_Toc33784562)

[7.1.1. 選択基準 18](#_Toc33784563)

[7.1.2. 除外基準 19](#_Toc33784564)

[7.1.3. 本登録基準 20](#_Toc33784565)

[7.2. 治験の中止 20](#_Toc33784566)

[7.2.1. 個々の被験者の中止 20](#_Toc33784567)

[7.2.2. 治験全体の中止又は中断 20](#_Toc33784568)

[8. 治験機器及び使用方法 20](#_Toc33784569)

[8.1. 治験機器 20](#_Toc33784570)

[8.1.1. 治験機器の名称等 20](#_Toc33784571)

[8.1.2. 治験における使用目的 21](#_Toc33784572)

[8.1.3. 治験機器の構成 21](#_Toc33784573)

[8.1.4. 表示 21](#_Toc33784574)

[8.1.5. 治験機器の取扱いと保管 21](#_Toc33784575)

[8.1.6. 使用上の注意 22](#_Toc33784576)

[8.1.7. リスクマネジメント： 22](#_Toc33784577)

[8.2. 治験機器の管理 23](#_Toc33784578)

[8.3. 使用遵守 23](#_Toc33784579)

[8.4. 治験機器以外の医薬品・医療機器の使用 23](#_Toc33784580)

[9. 被験者の同意 23](#_Toc33784581)

[9.1. 同意文書 23](#_Toc33784582)

[9.2. 被験者の意思に影響する重要な情報が得られた場合（説明文書・同意文書の改訂） 24](#_Toc33784583)

[10. 被験者の登録 24](#_Toc33784584)

[10.1. 被験者スクリーニング名簿の作成 24](#_Toc33784585)

[10.2. 登録手順 24](#_Toc33784586)

[10.3. 無作為化の方法及び盲検性の維持 24](#_Toc33784587)

[10.3.1. 開鍵の手続き 25](#_Toc33784588)

[10.3.2. 治験中の開鍵手続き 25](#_Toc33784589)

[11. 治験の観察・検査・調査項目、実施期間 26](#_Toc33784590)

[11.1. 治験スケジュール 26](#_Toc33784591)

[11.2. 被験者背景及びスクリーニング検査 26](#_Toc33784592)

[11.3. 最適刺激の検討 27](#_Toc33784593)

[11.3.1. 刺激に関する特記事項 27](#_Toc33784594)

[11.3.2. 刺激の種類 28](#_Toc33784595)

[11.3.3. 刺激方法 28](#_Toc33784596)

[11.3.4. 治療刺激 29](#_Toc33784597)

[11.3.5. 治療刺激方法 29](#_Toc33784598)

[11.3.6. 評価項目 30](#_Toc33784599)

[11.4. 併用制限薬・併用禁止療法 30](#_Toc33784600)

[11.5. 併用薬・併用療法 31](#_Toc33784601)

[11.6. 安全性の評価 31](#_Toc33784602)

[11.6.1. 有害事象・不具合の定義 31](#_Toc33784603)

[11.6.2. 有害事象・不具合及び重篤な有害事象・不具合の調査期間、頻度及び方法 31](#_Toc33784604)

[11.6.3. 重篤な有害事象の定義 32](#_Toc33784605)

[11.6.4. 重篤な有害事象・不具合発現時の対応 32](#_Toc33784606)

[11.6.5. 予想される副作用等 33](#_Toc33784607)

[12. データ解析と統計学的考察 33](#_Toc33784608)

[12.1. 治験デザインの検討 33](#_Toc33784609)

[12.1.1. 症例数の設定 33](#_Toc33784610)

[12.1.2. 症例数設定の根拠 33](#_Toc33784611)

[12.1.3. 被験者数の再推定 35](#_Toc33784612)

[12.2. データ解析の検討 35](#_Toc33784613)

[12.2.1. 完了例 35](#_Toc33784614)

[12.2.2. 解析対象集団 35](#_Toc33784615)

[12.2.3. 中間解析 36](#_Toc33784616)

[12.2.4. 有効性の解析手法 36](#_Toc33784617)

[12.2.5. 安全性の解析手法 38](#_Toc33784618)

[13. 治験の品質管理及び品質保証 39](#_Toc33784619)

[13.1. 同意取得手順を含む、規制及び倫理上の考慮事項 39](#_Toc33784620)

[13.2. 治験計画の規制当局への届出 39](#_Toc33784621)

[13.3. 治験実施計画の逸脱及び変更 39](#_Toc33784622)

[13.3.1. 治験実施計画書からの逸脱 39](#_Toc33784623)

[13.3.2. 治験実施計画書の変更 39](#_Toc33784624)

[13.4. 品質管理 40](#_Toc33784625)

[13.4.1. モニタリング 40](#_Toc33784626)

[13.4.2. データマネジメント 40](#_Toc33784627)

[13.5. 品質保証 41](#_Toc33784628)

[13.6. 実施医療機関における治験の中止 41](#_Toc33784629)

[13.7. 記録の保存 42](#_Toc33784630)

[13.8. 治験責任医師への治験結果及び情報の提供 42](#_Toc33784631)

[13.9. 治験実施期間 42](#_Toc33784632)

[14. 治験実施体制 43](#_Toc33784633)

[15. 引用文献 43](#_Toc33784634)

[16. 別添1　Dynamic Gait Index 44](#_Toc33784635)

[17. 別添2　日本語版modified Fall Efficacy Scale 49](#_Toc33784636)

[18. 別添3　日本語版Dizziness Handicap Inventory 50](#_Toc33784637)

# 略号及び用語の定義

| BMI | Body-Mass Index（肥満度指数） |
| --- | --- |
| GCP | Good Clinical Practice（医療機器の臨床試験の実施の基準） |
| GVS | Galvanic Vestibular Stimulation（前庭電気刺激） |
| LED | Light Emitting Diode（発光ダイオード） |
| RMS | Root Mean Square （全重心動揺の平均位置と計測された重心位置の距離の二乗平均の平方根） |
| DGI | Dynamic Gait Index |
| DHI | Dizziness Handicap Inventory |
| FAS | Full Analysis Set（最大の解析対象集団） |
| mFES | modified Fall Efficacy Scale |
| PPS | Per Protocol Set（治験実施計画書に適合した解析対象集団） |
| SAS | Safety Analysis Set（安全性の解析対象集団） |

#

# 緒言

## 背景・経緯

１）対象疾患の疫学、発症機序と予後

わが国における高齢者の転倒・転落による年間死亡者数は毎年増加しており、2009年には約7,300人と、交通事故による死亡者数を超えている。高齢者の死亡・寝たきりの主な原因は転倒・骨折であり、その予防は厚生労働行政の重要な課題の一つとなっている。特に骨折に伴う寝たきりは認知症発症の原因の一つでもあり、効果的な高齢者転倒防止策の開発は、急速な高齢化社会を迎えつつあるわが国にとって喫緊の課題である。

高齢者の転倒の原因の一つは、めまい・平衡障害であるが、その約40%は末梢前庭障害に起因すると言われている。めまい・平衡障害を有する高齢者の転倒リスクは、そうでない高齢者の約3倍と報告されており、前庭障害の治療は高齢者の転倒・骨折の予防という観点から極めて重要である。

前庭障害は平衡感覚を司る内耳にある前庭が障害を受けることによって生じる疾患であり、主な症状は著しいめまい、ふらつき、歩行困難などである。それ自体は生命予後に大きな影響は与えないものの、患者のADLに影響を与えQOLを著しく害する。さらに上述したように、特に高齢者の場合は転倒リスクを高める可能性がある。

前庭障害の正確な患者数は不明であるが、ドイツでの電話インタビューによる推計では前庭障害によるめまい患者は成人の約8%と報告されている^1）^。また、米国における前庭障害のスクリーニング検査では、成人の35%で何らかの前庭障害を有しており、めまい症状の有無にかかわらず、転倒の危険性が12倍に高まることが報告されている^2)^。

２）対象疾患の現状の治療法とその問題点

一側の末梢前庭障害によるめまい・平衡障害に対しては、リハビリテーションによる前庭代償の促進が有効とされる。一側の前庭障害においては、前庭機能の回復が無くても、時間経過とともにめまい・平衡障害の症状が徐々に軽快していく前庭代償という現象が知られており、小脳・脳幹を中心とする中枢神経系の可塑性に基づいて達成されると考えられている。前庭リハビリテーションにおいては、積極的に身体を動かし、体平衡に関与する前庭系・眼運動系・深部知覚系に有効な刺激を与えることによって、前庭代償の促進を促し、めまい症状や動的状態に平衡障害の改善を図るものである。この前庭リハビリテーションによって、ある程度の平衡障害の改善は図れるものの、完全にめまい・平衡障害が消失するとは限らない。また、一側前庭障害においても、前庭リハビリテーションが有効でなく、めまい・平衡障害が永続的に残存する症例も少なからず存在する。

また、両側の末梢前庭障害に関しては、前庭リハビリテーションの効果も乏しく、これまでに有効な治療が無いのが現状である。根本治療として、前庭再生の研究が行われているものの、未だ研究段階であり、臨床への応用には至っていない。また、難治性の両側前庭障害に対する治療として3つの半規管内に刺激電極を挿入して、半規管の神経を直接電気刺激することによって、前庭機能の代替を行う人工前庭が欧米で開発されつつあるが、治験の報告は無く、治療法としてのエビデンスは確立されていない。またこの治療は外科的侵襲を伴い、半規管内への電極の埋め込みによって難聴や更なる前庭障害を生じる危険性があり、さらに高額の治療費を要する。従って、より安全で簡便な治療法の開発が強く望まれている。

３）治療法の原理とメリット

前庭電気刺激（galvanic vestibular stimulation: GVS）は、前庭神経を電流刺激する方法で、従来、実臨床では前庭機能検査に使用されている。ノイズGVSは、前庭刺激に使用する電流を微弱なノイズ電流とする方法である。近年、微弱な入力信号に対する非線形の応答がノイズを与えることによって補強されるという確率共振現象が注目されており^3）^（図2）、その原理の解明も進んでいるが^4）^、ノイズGVSは、この確率共振現象との関連が示唆されている。さらに、痛みや不快感などの副作用を伴わない程度の微弱なノイズGVSがパーキンソン病などの神経変性疾患における自律神経反射やパフォーマンスの向上に有効であることも示されている。携帯型のノイズGVSは、患者の耳後部に表面電極を貼付し、小さな刺激装置を用いて微弱な電流を流すことにより平衡障害の治療を行うため、外科的侵襲を伴わず、低コストであり、使用中に患者は刺激を感じない、など様々なメリットを有している。ノイズGVSが難治性の末梢前庭障害にも有効であることが示せれば、簡便かつ外科的侵襲を伴わない方法によって、めまい・平衡障害に苦しむ多数の患者の治療が可能になる。

図2　確率共振の原理

非線形性のシグナル応答は、適切な大きさのノイズを与えることによって補強される。

（左）閾値下のシグナルは情報をもたらさない。（中央）適切な大きさのノイズを与えることで、閾値上のシグナルが出現し、情報が増加する。（右）さらに大きなシグナルを与えると、情報は減少する。

## 非臨床試験の要約

### 機械的及び電気的試験

#### 電気的安全性

本治験機器の電気的安全性については、一般財団法人日本品質保証機構においてJIS T 0601-1:2017「医用電気機器 第1部：基礎安全及び基本性能に関する一般要求事項」の要求事項に従って実施され、いずれの項目においても規格を満たしており、電気的安全性が十分に確保されていると判断した。

#### 機械的安全性

本治験機器の機械的安全性については、一般財団法人日本品質保証機構においてJIS T 0601-1:2017「医用電気機器 第1部：基礎安全及び基本性能に関する一般要求事項」の要求事項に従って実施され、いずれの項目においても規格を満たしており、機械的安全性が十分に確保されていると判断した。

#### 電磁両立性

本治験機器の電磁両立性については、一般財団法人日本品質保証機構においてJIS T 0601-1-2:2012「医用電気機器　第1-2 部：安全に関する一般的要求事項－電磁両立性－要求事項及び試験」の要求事項に従って実施され、いずれの項目においても規格を満たしており、電磁両立性が十分に確保されていると判断した。

#### 神経及び筋刺激装置の基礎安全及び基本性能に関する個別要求事項

本治験機器の神経及び筋刺激装置の基礎安全及び基本性能については、一般財団法人日本品質保証機構においてJIS T 0601-2-10：2015「医用電気機器　第2-10 部：神経及び筋刺激装置の基礎安全及び基本性能に関する個別要求事項」の要求事項に従って実施され、いずれの項目においても規格を満たしており、個別要求事項が十分に確保されていると判断した。

### 生物学的安全性評価

本治験機器において、人体と接触する機器は、電極である。

本電極は、既承認品目であることから、今回、新たな生物学的安全性の評価は実施していない。

## 臨床試験の要約

### 自主臨床試験1及び2の成績

#### 短時間刺激の効果

健常成人21名と両側前庭障害患者11名に対してノイズGVSの立位時の体平衡に及ぼす影響について検討した。その結果、感覚閾値の80%程度の強さの刺激で、刺激が無い時とくらべて、健常者の76%、両側前庭障害患者の91%において、体平衡機能の改善が認められ、ノイズGVSにより外周面積の大きさは、健常者で38%、両側前庭障害患者で46%減少した。ノイズGVSによる体平衡の改善効果は、健常者よりも両側前庭障害患者の方が大きかった^5）^（[図3](#図2)）。

A.　耳後部に電極を貼付し、ノイズGVSを与えた状態で閉眼状態で重心動揺計の上に30秒間起立させた。

B.　両側前庭障害症例（ミトコンドリア脳筋症）におけるノイズGVSの効果。400μAのノイズGVSによって、バランス障害は著明に改善した。

**図3　両側前庭障害症例に対するノイズGVSのバランス改善効果**

#### 刺激の持続効果

我々は、2014年度に健常高齢者を対象にして刺激の持続効果を検討する自主臨床試験を

行った。その結果、30分間のノイズGVSに刺激終了後3時間の持続効果があることを明らかにした^6）^。

### 安全性

#### 自主臨床試験1の安全性

健常者及び両側前庭障害患者に対して、GVS-100を用いてノイズGVSを30秒間行ったが、刺激中も刺激後も有害事象は認められなかった^5）^。

#### 自主臨床試験2の安全性

健常者を対象にした自主臨床試験において、GVS-100を用いてノイズGVS長期刺激(30分及び3時間刺激)を行ったが、刺激中も刺激後も有害事象は認められなかった^7)^。

## 自主臨床試験3の成績^8)^

2016年10月～2017年1月に当院にて、両側前庭障害患者13例を対象として、経皮的ノイズGVSの30分間の長期刺激の安全性と、刺激終了後の持ち越し効果、最適刺激強度の変動の有無を、2週間の間隔を設けて2度検討する探索的臨床試験を行った。第1期では最適刺激のみ検討し、第Ⅱ期と第Ⅲ期でノイズGVS長期刺激の安全性及び持ち越し効果を検討した。

### 経皮的ノイズGVS終了後の持ち越し効果

両側末梢前庭障害患者において、30分のノイズGVSによる刺激後3時間において、総軌跡長がベースライン時より有意に改善した（第Ⅱ期の総軌跡長の変化量：[図](#参考文献3) 4）。混合効果モデルにおけるノイズGVS後の平均値とベースライン値を比較する対比の検定によると、第Ⅱ期、第Ⅲ期ともに、刺激終了後6時間後まで平均的に総軌跡長の有意な改善が見られた（[表1](#表1)）。また、5段階で測定された自覚的な改善度についても改善傾向にあることが確認された（[図](#図4)5）。自覚的改善度は総軌跡長と相関関係があったことから、総軌跡長の臨床的な意義が示唆された。

**表1　ベースラインからの平均的変化についての解析**

| パラメータ |  | p値 | |
| --- | --- | --- | --- |
|  |  | 3時間後まで | 6時間後まで |
| 総軌跡長 | 第Ⅱ期 | <.001 | 0.004 |
|  | 第Ⅲ期 | <.001 | 0.002 |
| 自覚症状スコア | 第Ⅱ期 | 0.064 | 0.033 |
|  | 第Ⅲ期 | 0.023 | 0.020 |

**図4　総軌跡長の変化量の経時推移（閉眼時）**

**図 5　自覚症状スコアの変化量の経時推移（閉眼時）**

### 最適刺激強度の変動の有無

感覚閾値以下で、重心動揺計により測定される3パラメータすべてで改善があり、最も総軌跡長の改善がみられた電流強度を最適刺激と定義した。第2期、第3期のノイズGVSによる刺激前には全例で最適刺激が存在した。「最適刺激を全時点で測定できた症例」は8例で、「最適刺激が測定できなかった時点のある症例」は5例であった。

各症例における最適刺激は、100 µA～1000 µAの範囲であり、各測定時点において一定の値で推移する症例は少なく、最適刺激の日間変動が認められた。また、最適刺激が一日の間で低下傾向にあることも確認された。

### 安全性

報告されたすべての有害事象の一覧を、[表](#表2)2に示した。

重篤な有害事象及び重篤な有害事象に至る不具合の報告はなかった。

有害事象は13例中2例に3件認められた。有害事象の内訳は、脳梗塞、眩暈感及び右難聴が各1件認められた。そのうち、ノイズGVSとの因果関係が否定できない事象（副作用）はなかった。

有害事象の重症度は、中等度（脳梗塞）又は軽度（眩暈感、右難聴）であり、高度はなかった。いずれの事象も、重篤ではなく、中止及び処置なく回復した。

なお、有害事象を伴わない不具合はなかった。

表 2　有害事象の一覧

統計解析報告書：Listing 6.1_1参照

| 性別/  年齢  (歳) | 身長  (cm)/  体重  (kg) | 発現日/  コメント | 有害  事象名 | 重症度 | ノイズGVSとの因果関係 | 重篤度 | 中止の有無 | 処置の有無 | 転帰日/  転帰 |
| --- | --- | --- | --- | --- | --- | --- | --- | --- | --- |
| 男  78 | 154/  47 | 2016-11-11/  第Ⅲ期翌日（試験期間終了後）に発生 | 脳梗塞 | 中等度 | 因果関係  がない | 重篤でない | なし | なし | 2016-11-27/  回復 |
| 男  45 | 165/  58 | 2016-11-08/  第Ⅱ期当日、開始前に発生 | 眩暈感 | 軽度 | 因果関係  がない | 重篤でない | なし | なし | 2016-11-08/  回復 |
| 男  45 | 165/  58 | 2016-10-28/  第Ⅱ期当日、開始前に発生 | 右難聴 | 軽度 | 因果関係  がない | 重篤でない | なし | なし | 2016-11-08/  回復 |

## 治験の位置付け

今回の治験においては、対象を両側前庭障害患者から、重度の体平衡障害を有する一側性を含む前庭障害患者に広げ、ノイズGVSの刺激中の体平衡機能改善効果を主目的として、副次的に刺激終了後の持ち越し効果、並びに安全性について検討し、前庭機能障害に伴う重度のふらつきを有する患者の転倒リスクを減少させ、QOLを改善する可能性のある治療機器を開発することである。

# 目的

主要目的：

本治験の主要目的は、前庭機能障害に基づく重度のふらつき症状を有する患者を対象に、携帯型前庭電流刺激装置による経皮的ノイズ前庭電気刺激（ノイズGVS［Galvanic Vestibular Stimulation］）を4時間加え、刺激中の体平衡機能改善効果について、プラセボ刺激との比較検討を行うことである。

副次目的：

本治験の副次目的は、ノイズGVSを4時間加え、刺激中及び4時間刺激終了3時間後までの歩行機能及び自覚症状の改善などの有効性、並びに刺激中・刺激後の安全性を検討することである。

# 治験の評価項目

## 主要評価項目

重心動揺計（アニマ社製、Gravicorder G-620型）を用いて測定した、治験機器によるノイズGVS開始直後、30分後、1時間後、2時間後、3時間後の全時点における総軌跡長のベースラインからの変化率

【Primary endpoint】

GVS期の刺激開始直後から刺激3時間後までの総軌跡長の変化率の平均値と、プラセボ期における刺激開始直後から刺激3時間後までの総軌跡長の変化率の平均値の差を比較する。

【設定根拠】

自主臨床試験で測定された3時間後までの総軌跡長変化量の平均値と、自覚的改善度の合計点（とりうる範囲は[-10, 10]、高いほど自覚的なふらつきの改善を表す）の間の相関係数は-0.60（p = 0.032）であったことから、総軌跡長の臨床的な有用性が示唆された。本治験では4時間にわたるノイズGVS中に5時点（ノイズGVS開始直後、30分後、1時間後、2時間後、3時間後）の総軌跡長を測定するため、5時点における総軌跡長のベースラインからの変化率を主要評価項目と設定した。

## 副次評価項目

1. 有効性の副次評価項目は、以下の通りである。
2. ノイズGVS開始直後、30分後、1時間後、2時間後、3時間後の全時点における外周面積、RMS（ Root Mean Square［全重心動揺の平均位置と計測された重心位置の距離の二乗平均の平方根］）値のベースラインからの変化率
3. ノイズGVS開始4時間後、5時間後、6時間後、7時間後の全時点における総軌跡長、外周面積、RMS値のベースラインからの変化率
4. 歩行機能

ノイズGVS開始直後、1時間後、2時間後、3時間後、4時間後、5時間後、6時間後、7時間後における以下の項目のベースラインからの変化率

- - Dynamic Gait Index（DGI Short version）のスコア^9)^（別添1）
  - 歩行分析計（WALK-MATE LAB株式会社、Walk Mate Viewer）を用いて測定する10メートル歩行時の歩行速度、歩幅、ステップ時間及び左右の揺れ幅

1. 自覚的改善度スコア

ノイズGVS開始直後、1時間後、2時間後、3時間後、4時間後、5時間後、6時間後、7時間後における以下の項目

- 被験者自身が刺激前の状態と比較して「1：改善、2：やや改善、3：不変、4：やや悪化、5：悪化」の5段階での評価

1. Quality of Life (QOL)

ノイズGVS開始3時間後及び7時間後における以下の項目のベースラインからの変化量

- 日本語版modified Fall Efficacy Scale（mFES）（別添2）
- 日本語版Dizziness Handicap Inventory（DHI）（別添3）

1. 活動量

ノイズGVS開始直後から3時間後、及び7時間後までの活動量（歩行数）

【設定根拠】

1. 外周面積、RMSは総軌跡長以外の重要な重心動揺パラメータであるため。
2. ノイズGVS開始前と長期刺激終了直後の比較を行うため、及び刺激終了後の重心動揺に関する持ち越し効果を検討するため。
3. DGIはvalidateされた歩行機能検査であり、歩行速度、歩幅、ステップ時間と併せて客観的に歩行機能を計測するため。
4. 被験者自身が感じている改善度を簡便な5段階の評価を用いて測定する。
5. 転倒恐怖感及びめまいを、validateされたQOL調査票を用いて測定する。
6. 活動量と他の評価項目との相関を確認するため。
7. 安全性評価項目は以下の通りである。

有害事象及び不具合の発生を評価する。

# 治験の計画

## 治験デザイン

本治験は前庭機能障害に伴う重度のふらつきを有する患者を対象とした多施設共同、二重盲検、ランダム化プラセボ対照クロスオーバー試験である。

### スクリーニング期間

治験責任（分担）医師は文書同意が得られた被験者について被験者の適格性を確認し、組み入れ基準（選択基準を満たし及び除外基準に抵触しない）を満たした被験者を、「仮登録」する。

### 最適刺激の検討

仮登録された被験者毎のノイズGVSによる最適刺激を決定する。最適刺激が決定した被験者を「本登録」する。一方、最適刺激が存在しない被験者はこの時点で中止し、2週後の安全性を確認し終了となる。

### 治療刺激期間

本登録された被験者は、本登録後2週間後に来院し、『第Ⅰ期』に最適刺激のノイズGVSによる体平衡機能改善の効果を評価した後に、『第Ⅱ期』にプラセボ刺激による評価を行う群（A群）と、その逆の順序で『第Ⅰ期』にプラセボ刺激による体平衡機能改善の効果を評価した後に、『第Ⅱ期』に最適刺激のノイズGVSによる評価を行う群（B群）の2群に1：1の比率でランダム割り付けされる。なお、本登録から第Ⅰ期まで、最低7日間の期間を空けることとする。

各被験者に、携帯型前庭電流刺激装置による刺激（「被験者毎の最適刺激」又は「プラセボ刺激」）を4時間加え、刺激前及び刺激中におけるふらつきと安全性を測定する。さらに、刺激後3時間についても安全性と有効性を評価することとし、全ての有害事象及び不具合の確認を行う。なお、第Ⅰ期の評価の後、第Ⅱ期の評価までに14日、最低7日間の期間を空けることとする。

なお、安全性については、刺激装置の使用時に生じた全ての有害事象及び不具合の確認を行う。

### 安全性の追跡調査期間

治療刺激期間の『第Ⅱ期』の評価終了2週間後に有害事象の有無についての聞き取り調査を行う。

## 治験デザインの考察

主要評価項目に使用する重心動揺検査は患者間のばらつきが大きいため、同一患者で経皮的ノイズGVS期とプラセボ刺激期の両方のデータを収集することで、患者間のばらつきを抑えて精度を高めることができること、前庭障害患者のうち、本治験の対象となるような重度のふらつきを有する患者数は、明確なデータはないものの非常に限定されると考えられること、及び重心動揺評価や歩行機能検査の手順がやや複雑なため、限られた施設数・患者数で最大限の結果の精度を確保するため、本治験はクロスオーバー試験としてデザインした。

ウォッシュアウト期間については、自主臨床試験3（4.4節）において、2週間の間隔を空けてノイズGVSを行えば、以降の重心動揺に影響が残らないことが示された。14日のウォッシュアウト期間を基本とするが、患者の都合によるプロトコル逸脱を最小限にするため、また経皮的ノイズGVSの刺激終了後の持ち越し効果の検討の結果、刺激後4時間以降では持ち越し効果が確認できなかったことから、最低7日間のウォッシュアウト期間でも許容できると考え、最低7日間と設定した。

なお、ノイズGVSの電流を0μAとした刺激をプラセボ刺激、感覚閾値以下で最も改善がみられた強度の電流を最適刺激とする。

# 被験者の選択と中止基準

## 被験者の組み入れ基準

以下の選択基準を満たし、除外基準に抵触しない難治性の前庭機能障害に基づく重度のふらつきを有する患者を対象とする。

### 選択基準

1. 氷水2 mLの温度刺激検査にて、一側あるいは両側の前庭障害を有する患者

一側障害はCP（Canal Paresis，半規管麻痺）(%)=(健側の最大緩徐相速度 - 患側の最大緩徐相速度)/(健側の最大緩徐相速度＋患側の最大緩徐相速度)x100 ≧ 20%、両側障害は眼振が誘発されないか、両側の最大緩徐相速度が10度/秒以下の末梢前庭障害患者とする。なお、CPの算出において、最大緩徐相速度の代わりに、眼振の持続時間を用いることも可とする。

1. 閉眼起立時の重心動揺計において、60秒間で総軌跡長が180cm以上の患者。
2. ふらつきを発症後1年以上経過し、リハビリテーション治療を行ってもふらつきの症状が少なくとも6か月以上持続する患者。
3. 年齢20歳以上85歳未満の前庭障害に伴う重度の患者とする。性別、難聴の有無は問わない。
4. 本治験の内容を理解し、本治験の参加前に自らの自由意思で文書同意した患者

【選択基準の設定根拠】

1. 前庭機能障害の定義については、従来より使用されている基準であり、国際的にも認められている。（両側前庭障害^5）^、一側前庭障害^6）^）
2. 重度のふらつきの基準としては、65歳以上の健常者の平均＋2SDの値とした（データはアニマ社提供）
3. 難治性のふらつきの基準として設定した。

4) ～5）安全性を考慮し年齢上限を設け、適切なインフォームドコンセントによる同意を得るために設定した。

### 除外基準

以下のいずれかの条件に該当する場合は、治験の組入れ対象としない。

1. 脳動脈クリップ、人工内耳やペースメーカーなど体内に金属を有する患者（銀歯は可）
2. 整形外科的疾患を有する患者（例として骨折、捻挫、肉離れ等、急性疼痛性疾患のある患者等）
3. 小脳障害や脊髄疾患による四肢の運動障害を有する患者
4. ペースメーカーが必要となりうる重度の不整脈（心房細動、重度のQT延長症候群、重度の房室ブロック（II度以上））、歩行に支障をきたすような重症の心不全など、重大な心疾患を有する患者
5. 悪性腫瘍を有する患者
6. 発熱、倦怠感又はふらつき等を伴う感染症疾患を有する患者
7. 妊娠中、出産直後の患者
8. 自力歩行が出来ない患者
9. 装着部位の皮膚に異常（感染症、創傷など）のある患者、又はアナフィラキシーショックの既往がある等、重度のアレルギー体質のある患者
10. 法的能力の欠如又は制限のある患者
11. 同意取得日より前3ヶ月以内に他の治験（臨床試験）に参加した者、又は治験と同時期に他の治験（臨床試験）に参加する患者
12. その他、治験責任（分担）医師が被験者として不適当と判断した患者

【除外基準の設定根拠】

　1)～6)、9)、11) 被験者の安全性確保のため設定した。7) 妊婦及び胎児への影響が完全には否定できないため、及び安全性確保のため設定した。8) 評価に影響を及ぼす可能性があるため設定した。10) 倫理的配慮より設定した。

### 本登録基準

ノイズGVSにて最適刺激が存在する患者

【本登録基準の設定根拠】

有効性評価のため設定した。

## 治験の中止

### 個々の被験者の中止

中止基準：

治験期間中、被験者が以下に該当した場合、治験責任（分担）医師は当該被験者の治験を中止する。

1. 選択基準を満たさない、あるいは除外基準に抵触していることが治験開始後に判明した場合
2. 有害事象の発現により治験責任（分担）医師が治験の継続を困難と判断した場合
3. 被験者が治験中止を希望、又は治験参加の同意を撤回した場合（脱落）
4. 被験者が著しく治験実施計画書の規定を遵守しなかった場合
5. 被験者が治験実施施設に継続的に再来院しなかった場合（追跡不能）
6. 自ら治験を実施する者が治験を中止した場合、治験審査委員会（以下、IRB）又は厚生労働省に中止するよう指示された場合
7. その他、治験責任（分担）医師が治験の継続が困難又は被験者に不利益が生じると判断した場合

### 治験全体の中止又は中断

自ら治験を実施する者は、以下の事項に該当する場合は治験実施継続の可否を判断する。

1. 治験機器の品質、安全性、有効性に関する重大な情報が得られたとき。
2. 被験者のリクルートが困難で予定症例を達成することが到底困難であると判断されたとき。
3. IRBにより、実施計画等の変更の指示があり、これを受入れることが困難と判断されたとき。

また、IRBにより、中止の勧告あるいは指示があった場合は、治験を中止する。

なお、治験の中止又は中断を決定した時は、速やかに病院長（あるいは各医療機関の長）にその理由とともに文書で報告する。

# 治験機器及び使用方法

## 治験機器

### 治験機器の名称等

1. 治験機器の名称

携帯型前庭電流刺激装置（GVS-100）

1. 被験機器の法規制上のクラス分類

- 類別：器具器械 12 理学診療用器具
- 一般的名称： 低周波治療器
- クラス分類：クラスII

### 治験における使用目的

前庭機能障害に基づく重度のふらつきの改善

### 治験機器の構成

本機器「携帯型前庭電流刺激装置」は、①刺激装置本体、②電極ケーブルにより構成されている。電極*A（ブルーセンサー　N-00-S/25、製造販売元　株式会社メッツ）、電極*B（ブルーセンサー　NF-00-S/12、製造販売元　株式会社メッツ）、又は電極*C（ディスポ電極 Fビトロード、製造販売元　日本光電工業株式会社）を耳後部の皮膚に貼付し、電極ケーブルを通じて刺激装置本体に接続して電気刺激を与える。

*：電極はディスポーザブルであり市販されているものを使用する。

### 表示

　治験機器の刺激装置本体にはラベルが1枚貼付されており、下記に掲げる事項が邦文で記載されている。

治験用である旨

治験調整医師の氏名及び職名並びに住所

原材料名又は識別記号

製造番号又は製造記号

貯蔵方法

### 治験機器の取扱いと保管

治験機器は、安全な場所に適切な物理的条件下で保管する。治験機器の取扱い及び管理は、治験責任（分担）医師及び許可された実施医療機関の職員以外は行わない。治験機器は、治験実施計画書に従って、治験に組入れられた被験者のみに使用する。治験機器の取扱い及び管理は、自ら治験を実施する者から提供された「治験機器概要書」及び「治験機器管理に関する標準業務手順書」に従い、適切に取扱う。

#### 使用方法

準備

1. 被験者の耳の後ろの皮膚を清拭し、乾燥させる。
2. 電極を左右の乳様突起部に貼付する。
3. 電極ケーブルを接続し、絆創膏等で補強する。
4. 使用した電極の種類を記録する。

使用中

1. 電源スイッチを入れる。
2. 電源ON/BATT LEDが2秒周期で点灯（正常動作）を確認する。
3. 電源ON/BATT LEDが正常動作をしていないときは使用を中止する。
4. 規定した刺激時間が経過したら、電源を切り、電極を外し、使用を終了する。
5. 電極外れが発生したときはLEDが0.4秒周期の早い点滅になる。この時電流出力は停止する。復旧方法は一度電源を切って、電極を正常に装着し直し、電源を切ってから6秒以上経ってから電源を再投入する。電源を投入してLEDが2秒周期のゆっくりした点滅になれば正常動作している。尚、LEDの点滅については、点灯しっぱなし、又は点灯しないという異常がごく希ですが確認されている。この場合も上記復旧方法に従って復旧させる。

### 使用上の注意

**機器の使用時の注意事項**

- 機器全般及び患者に異常がないか監視する。
- 機器及び患者に異常が発見された場合には、患者の安全を確保し、機器の使用を中止する。
- 電極は、ディスポーザブル製品なので再使用しないこと。

波形B、レンジ2mA、出力調整99%における電池寿命の平均で16時間程度である。1回4時間使用で、4回使用目安に電池交換する。電池の電圧が低下するとLEDの点滅は0.8秒周期となるので電池交換を行う。

**・その他の注意事項**

- 長期間使用しなかった機器を再使用する時は、使用前に必ず機器が正常かつ安全に作動することを確認すること。感電や故障の原因となる恐れがある。
- 故障した時は勝手に操作せず、適切な表示を行い、修理業者に連絡する。
- 機器を勝手に改造したり、また他社の機器との接続はしないこと。

#### 貯蔵・保管方法及び使用期限

**貯蔵・保管方法**

- 水等のかからない場所に設置すること。
- 気圧、温度、湿度、風通し、日光、ほこり、塩分、イオウ分を含んだ空気等により悪影響の生ずる恐れのない場所に設置すること。

使用期限

出荷可否判定日から1年とする。その後は1年毎の定期点検を行い、定期点検日から1年とする。

### リスクマネジメント：

#### 残留リスクの特定を含むリスク分析の概要

製造元で実施されたリスク分析の結果、本治験機器のリスクとしては、①出力停止による性能の消失、②電池の逆接続による回路機能の停止、③電源ラインショートによる電池発熱による火傷の危険性が考えられている。

これらのリスク低減手段として、①については定期保守による規格の維持、②についてはシリーズダイオードの追加と極性ラベルの表示、③についてはチップフューズの追加を実施することにより、残留リスクの低減が行われた。

#### リスクアセスメントの結果

本治験機器のリスクマネジメントをJIS T 14971: 2012「医療機器 - リスクマネジメントの医療機器への適用」に従って、製造元において予め規定されたリスク分析手順で行った結果、特定した各ハザードにおいて、推定したリスクについて、適切なレベルまで低減することにより、受容できないと判断される残留リスクは存在しなかった。

## 治験機器の管理

治験責任（分担）医師、指名された実施医療機関の職員、又は（該当する場合は）実施医療機関の長は、被験者に使用した治験機器の数量、自ら治験を実施する者から受領した数量及び自ら治験を実施する者に返却した数量を適宜記録する。治験機器の管理記録は治験期間を通して保管する。

実施医療機関の治験機器管理者は、すべての治験機器を、自ら治験を実施する者から提出された「治験機器管理に関する標準業務手順書」に従い、適切に取扱う。

## 使用遵守

治験機器の使用は治験責任（分担）医師の監督下で実施する。各被験者の使用状況に関連する情報を症例報告書に記録する。

## 治験機器以外の医薬品・医療機器の使用

治験機器以外に薬剤又は医療機器を使用する必要があると治験責任（分担）医師が判断した場合、治験責任（分担）医師は、薬剤名又は医療機器名（可能であれば商品名）、用法、用量、投与経路、投与日、使用目的を症例報告書に記録する。ただし、抗不安薬、抗うつ薬、睡眠薬、鎮暈薬及び効能効果にめまいの適応のある薬剤の新規の使用は禁止する（11.4項参照）。

# 被験者の同意

　適用されるすべての規制要件に従った以下の内容と手順により、被験者が治験に参加する前に同意を取得する。

## 同意文書

　本治験のスクリーニング検査の開始に先立ち、治験責任（分担）医師は被験者として適切と思われる人に治験審査委員会により承認された説明文書及び同意文書を用いてGCPで規定されている被験者に対する説明事項を十分に説明する。その際、質問する機会と、治験に参加するか否かを判断するのに十分な時間を与えるものとする。その上で、被験者の自由意思による同意が得られた後、説明文書及び同意文書に被験者の署名又は記名捺印、及び同意日を入手する。さらに説明者は、説明日を説明文書及び同意書に記入し、署名又は記名捺印する。また、治験協力者が補足的な説明を行った場合には、治験協力者も説明日を説明文書及び同意書に記入し、署名又は記名捺印する。治験責任（分担）医師は、上記の署名又は記名捺印と日付が記入された説明文書及び同意書の原本をカルテなどの原医療記録に添付（実施医療機関で保管の定めがある場合はそれに従う）し保存、写しを被験者に渡す。

　同意取得前の温度刺激検査を治験データとして使用する場合、同意取得前から3年以内とする。

## 被験者の意思に影響する重要な情報が得られた場合（説明文書・同意文書の改訂）

　被験者の同意に関連し得る新たな重要な情報（通常、説明文書・同意文書の改訂を必要とする情報）が得られた場合には、治験責任医師等は、当該情報を直ちに被験者に伝え、治験継続の意思を確認し、被験者に伝えられたことを文書に記録する。また、治験責任医師は治験依頼者の協力のもと速やかに当該情報に基づき説明文書・同意文書を改訂し、被験者への説明を行う前に治験審査委員会の承認を得る。

治験責任医師等は、改訂された説明文書・同意文書を用いて改めて説明し、治験への参加の継続について被験者から自由意思による再同意を文書により得なければならない。

治験責任医師等は、新たに記名押印又は署名と日付を記入した同意文書の写し及び説明文書を被験者に渡さなければならない。

同意文書の原本を実施医療機関で保存する。治験責任医師等は、同意文書の写し及び説明文書を被験者に手渡したことを記録する。

# 被験者の登録

## 被験者スクリーニング名簿の作成

治験責任医師等は、同意取得した全ての被験者に対して被験者識別コード、被験者名、カルテ番号、同意取得年月日、説明文書・同意文書の提供の有無等を記載した被験者スクリーニング名簿を作成する。このうち、最適刺激の検討を行った被験者については、治験の終了又は中止についても記載する。

## 登録手順

文書同意が得られ、スクリーニングで適格性が確認された全ての被験者の登録を行う。被験者識別コードを用いた中央登録方式とし、症例登録はすべてweb上で行う。本治験では仮登録と本登録を行う。

1. 仮登録

文書同意が得られ、スクリーニングで適格性が確認された全ての被験者を仮登録する。

1. 本登録

最適刺激の検討において、最適刺激が存在した被験者のみ本登録を行う。最適刺激が存在しない被験者はこの時点で中止し、2週後の安全性を確認し終了となる。

## 無作為化の方法及び盲検性の維持

治験に関わるすべての者（治験機器の無作為化割付け作業を行ったものを除く）は、本治験の無作為化情報に対し盲検化される。

本登録された被験者に対して、Web上でブロックランダム化により1：1の比率で2群（A群又はB群）に割付ける。スクリーニング時の総軌跡長（60秒当たり200cm未満、以上）、一側／両側前庭障害を割付因子として設定する。本割付けの手順は別途作成する「治験機器無作為化割付けに関する手順書」に従い、治験責任医師が指名した割付責任者が実施する。第Ⅰ期及び第Ⅱ期について、登録システム上で各被験者がランダム化された結果を、当該施設の治験責任医師等以外の者（割付担当者と呼ぶ）が確認する。治験責任医師等はスクリーニング期に測定した最適刺激を割付担当者に伝え、割付担当者は割付結果に従って、プラセボ刺激もしくは最適刺激に設定した機器（プラセボ刺激か実刺激かは、被験者及び治験責任医師等には識別不可能）を渡す。治験責任医師等は設定された刺激を30秒流し、感覚閾値以下であるかを確認する。確認する方法として、ノイズGVSに特異的な刺激（少し間隔のある、連続した刺激）を感知するか否かを患者に尋ねる。感覚閾値を超えている場合には、以降のデータについて収集は継続するが、主要の解析からは除く（ただし、それまでに測定されているデータは用いる）。感覚閾値以下の場合、又は感覚閾値を超えても患者が不快と感じない場合には、その強度の刺激を4時間持続的に流す。刺激中は所定の場所で待機してもらい、刺激電極や刺激機には触れないように指示する。また、刺激中の評価項目測定時点においても、感覚閾値以下であるかを確認し、もし感覚閾値を超える場合にはその時点の前までのデータを主要な解析に含める。感覚閾値を超えたことが判明した後のデータは、補足的な解析に用いる。割付責任者及び割付担当者は開鍵時まで無作為化割付情報を秘匿する。

なお、プラセボ刺激の際にも、治験責任医師等及び被験者がプラセボ刺激であることがわからないようにするため、前庭電気刺激機の点滅は、実際に刺激を行っている場合と同様にLEDランプが点滅するよう設定する。

### 開鍵の手続き

すべての症例報告書の作成が終了し、解析のためのデータが固定された後に治験機器割付責任者が開鍵する。

### 治験中の開鍵手続き

治験責任医師等は、重篤な有害事象が発現し、被験者の安全性確保のため割付情報を知る必要があると判断した場合、当該被験者の割付情報を知ることができる。当該被験者の割付情報を開示した場合、治験責任医師等は、割付情報を開示したことを速やかに割付責任者に連絡する。割付情報を開示した場合、当該被験者のノイズGVSを中止する。また、治験責任医師等は割付情報を開示した場合、開示日及び理由を記録する。緊急の事態を回避した後、治験責任医師等は、割付情報の開示について実施医療機関の長に連絡する。治験実施途中で個別に割付情報を開示した場合、その実施医療機関で実施されている他の症例の盲検性、取扱い及び今後の方針については、割付責任者が決定する。

# 治験の観察・検査・調査項目、実施期間

## 治験スケジュール

表3　治験スケジュール

## 被験者背景及びスクリーニング検査

文書同意が得られ、組み入れ基準（選択及び除外基準）を満たし、スクリーニングで適格性が確認された被験者を仮登録する。

1. 被験者背景

被験者識別コード、性別、生年月、身長、体重、既往歴、合併症、併用薬（療法）について調査する。

1. 自覚症状、他覚所見

問診、診察（体温・血圧測定を含む）にて確認する。

1. 温度刺激検査

氷水2 mLを外耳道に20秒間注水し、外側半規管の刺激を行う。刺激によって誘発される眼振を電気眼振図計あるいはビデオ眼振図計にて記録し、眼振の緩徐相速度の測定を行う。一側注水後には、少なくとも5分間の間隔を空けて、対側の刺激を行う。

一側障害はCP(%)=(健側の最大緩徐相速度 - 患側の最大緩徐相速度)/(健側の最大緩徐相速度＋患側の最大緩徐相速度)ｘ100 > 20%、両側障害は両側の最大緩徐相速度が10度/秒以下の末梢前庭障害患者とする。

1. 重心動揺検査

フォームラバーを置かない（体性感覚入力を遮断せず）重心動揺計に開眼起立した状態で重心動揺を60秒間測定する。その後、閉眼起立し、身体動揺が定常状態に達した後に60秒間重心動揺を測定する。

閉眼起立時の重心動揺計において、60秒間で総軌跡長が180cm以上を重度のふらつきと定義する。

- 1. **最適刺激の検討**

仮登録された被験者毎のノイズGVSによる最適刺激を決定する。最適刺激検討スケジュールを表4に示した。

最適刺激の検討のための刺激前、刺激中、刺激後に有害事象、刺激中に不具合の確認を行う。

最適刺激が決定した被験者を「本登録」する。一方、最適刺激が存在しない被験者はこの時点で中止し、2週後の安全性を確認し終了となる。

表4　最適刺激検討スケジュール

### 刺激に関する特記事項

刺激開始前の確認事項：

急性疾患を有する者、体温38℃以上（有熱期）の者、血圧に異常がある者（180 mmHg以上）、治験前日22時以降に飲酒した者には刺激を行わないこととする。その場合、スケジュールを許容範囲内で再調整する。再調整できない場合は終了とする。

### 刺激の種類

**被験刺激：**

被験者毎の最適刺激：電流100、200、300、500、700、1,000、1,200、1,500、1,700、2,000 μAの10個の刺激強度のうち、感覚閾値（ノイズGVSに特異的な刺激（少し間隔のある、連続した刺激）を感じた最小の電流強度）より下で、総軌跡長が短くなった複数の電流の強さのうち、重心動揺検査で総軌跡長が最も改善した1,000μA以下の強度の電流とする。

被験者の訴えにより中断する場合は、それまでに行った刺激の中から最適刺激の判定を行う。

**対照刺激：**

プラセボ刺激として、電流0 μAで刺激を行う。

【刺激電流の設定根拠】

2015年に健常高齢者を対象にして3時間刺激の刺激を行い、刺激中及び刺激後の持続効果を検討する自主臨床試験を行った結果、刺激中及び刺激4時間後までの間、総軌跡長の改善効果があることを明らかにした。ノイズGVSを使用した他の研究において、2,000 µAまでの強度で刺激を行っている研究は多く見られ、有害事象などの報告もなされていないことから感覚閾値の検討のための上限を2,000 μAとし、最適刺激の上限を長時間の刺激実績がある1,000 μAとした^10-12)^。

- - 1. **刺激方法**

**最適刺激の検討：**

この最適刺激を決定するために、重心動揺検査を実施する。感覚閾値下（ノイズGVSに特異的な刺激（少し間隔のある、連続した刺激）を感じた最小の電流強度）より下で、連続した複数の電流強度において総軌跡長が短くなり、そのうち最も総軌跡長が改善した電流強度において総軌跡長がベースライン時（電流を流さない状態で閉眼起立したときの3回の測定の平均値）と比較して10%以上改善した症例を最適刺激が存在する症例と定義し、「本登録」に進めることとする。また、最適刺激は上記複数の電流のうち、最も総軌跡長が改善した電流を最適刺激と定義する。

最適刺激の検討手順

1) フォームラバーを置かない（体性感覚入力を遮断せず）重心動揺計に電流を流さない状態で閉眼起立し、身体動揺が定常状態に達した後に30秒間重心動揺を測定する。

2) 1)の測定を2分の間隔を空けて3回実施する。

3) 測定後、重心動揺計から降り2分の間隔を空けた後、100、200、300、500、700、1,000、1,200、1,500、1,700、2,000 μAの電流を流した際の重心動揺を上記と同様の方法で30秒間測定する。

100 μA ～1,000 μA の6つの刺激強度において、感覚閾値下で、総軌跡長が改善した連続した複数の電流強度のうち最も総軌跡長が改善した電流強度を最適刺激とする。被験者の訴えにより中断する場合は、それまでに行った刺激の中から最適刺激の判定を行う。なお、最大2,000 μAまで電流強度を上げ、痛みを感じるか否かを調べる。ただし、痛みを感じた時点で終了する。ノイズGVSに特異的な刺激を感じた最小の電流強度を感覚閾値とする。

最適刺激の検討のための刺激前、刺激中、刺激後に有害事象と、刺激中に不具合の確認を行う。

最適刺激が決定した被験者を「本登録」する。一方、最適刺激が存在しない被験者はこの時点で中止し、2週後の安全性を確認し終了となる。

### 治療刺激

本登録された被験者に、当該被験者に割付けられたノイズGVS（「被験者毎の最適刺激」又は「プラセボ刺激」）を行う。本登録の2週間後に『第Ⅰ期』の刺激を行い、効果と安全性の評価を行う。第Ⅰ期の評価の後、7日間以上の期間を空けて『第Ⅱ期』の刺激を行い、同様に効果と安全性の評価を行う。

表5　治療・持ち越し効果の検討スケジュール

### 治療刺激方法

被験者毎に定まった最適刺激の電流又はプラセボ刺激の電流（0 μA）を4時間持続的に流す。なお、刺激中は、所定の場所で待機してもらい、刺激電極や刺激機には触らないように指示する。

### 評価項目

第Ⅰ期及び第Ⅱ期では表3に示したスケジュールで評価項目を実施する。

#### 重心動揺検査

刺激前、刺激中、刺激後にはフォームラバーを置かない重心動揺計に、身体動揺が定常状態に達した後に30秒間重心動揺を測定する。

測定するタイミングは刺激直前、ノイズGVS開始直後、30分後、1時間後、2時間後、3時間、4時間後、5時間後、6時間後及び7時間後の10回計測を行う。

重心動揺計計測中に大きくバランスをくずして、両上肢を体側に接した姿勢が維持できない、あるいは重心動揺計から落ちてしまった場合には、再度計測を行う。

#### 歩行機能検査（可能な限り実施）

刺激直前、ノイズGVS開始直後、1時間後、2時間後、3時間後、4時間後、5時間後、6時間後、7時間後の9回、別途定める手順書に従い以下の計測を行う。

1. 歩行分析計（WALK-MATE LAB株式会社、Walk Mate Viewer）を用いて測定する10メートル歩行時の歩行速度、歩幅、ステップ時間及び左右の揺れ幅
2. DGI （Short Version）(別添１)のスコア

#### 自覚的改善度評価

ノイズGVS開始直後、1時間後、2時間後、3時間後、4時間後、5時間後、6時間後、7時間後の8回、被験者自身に刺激前の状態と比較して「1：改善、2：やや改善、3：不変、4：やや悪化、5：悪化」の5段階で評価させる。刺激直前は実施しない。

#### QOL

刺激直前、ノイズGVS開始3時間後及び7時間後に日本語版mFES及び日本語版DHIを使用し評価する。

#### 活動量

ノイズGVS開始直後から3時間後、及び7時間後までの活動量（歩行数）について歩数計を用いて測定する。

## 併用制限薬・併用禁止療法

【実施時期】

同意取得時からWeek4又は中止時まで

【実施内容】

同意取得時からWeek4又は中止時まで、抗不安薬、抗うつ薬、睡眠薬、鎮暈薬及び効能効果にめまいの適応のある薬剤の新規の使用を禁止する。なお、最適刺激の検討日を起点として8週以上継続投与されている薬剤は、症状が安定している場合は用法用量を変更しないことを条件に、使用する。また、ふらつきの改善を目的とした新規のリハビリテーションの実施を同意取得時からWeek4又は中止時まで禁止する。なお、最適刺激の検討日を起点として8週以上継続されているリハビリテーションは、症状が安定している場合は、内容を変更しないことを条件に実施する。

## 併用薬・併用療法

【実施時期】

同意取得時からWeek4又は中止時まで

【実施内容】

同意取得時からWeek4又は中止時までに使用した薬剤又は療法について調査する。併用薬については、薬剤名、投与期間（頓用の場合、薬剤使用日が判明する場合は可能な限り薬剤使用日の情報を収集）、使用目的を調査し症例報告書に記載する。

## 安全性の評価

【実施時期】

有害事象については同意取得時からWeek6（追跡調査終了時）又は中止時まで、不具合については治験機器使用時

【実施内容】

スクリーニング及び治療刺激期間：

刺激前に診察により、自覚症状/他覚所見を確認する。また刺激前、刺激中、刺激後に有害事象と不具合の確認を行う。

安全性の追跡調査：

治療刺激期間の『第Ⅱ期』の評価終了2週間後に有害事象の有無についての聞き取り調査を行う。

### 有害事象・不具合の定義

「有害事象」とは、治験機器の使用時に被験者、使用者その他の者（以下、「被験者等」という。）に生じた全ての好ましくない又は意図しない疾病又は障害並びにその徴候（臨床検査値の異常を含む。）をいい、当該治験機器との因果関係の有無は問わない。ただし、被験者以外の者に生じたものについては、治験機器の使用による影響と疑われるものに限る。

「不具合」とは、破損、作動不良等広く品質、安全性、性能等に関する治験機器の具合がよくないことをいい、設計、交付、保管、使用のいずれの段階によるものであるかを問わない。

被験者等又は治験機器に有害事象又は不具合が発現した場合、被験者等の安全性の確保及び報告等について治験責任（分担）医師は直ちに適切な措置を講ずるとともに、重篤な有害事象に関しては、治験開始直前の状態もしくは正常に回復するまで追跡調査し、その結果を症例報告書に記録する。

### 有害事象・不具合及び重篤な有害事象・不具合の調査期間、頻度及び方法

治験責任医師又は実施医療機関職員は、有害事象又は重篤な有害事象の定義に合致する事象を検出し、記録、報告する責任を負う。

有害事象の収集期間は、同意取得時から追跡調査期又は中止時までとする。また不具合の収集期間は、治験機器使用時とする。

1） 有害事象

治験責任（分担）医師は、発現した有害事象に関する以下の項目について記録する。

- ノイズGVSとの因果関係（ノイズGVSとの因果関係がない、ノイズGVSとの因果関係が否定できない）
- 時期（発現日（時）*、転帰日又は転帰確認日）

*：時間は治験機器使用時の有害事象のみ記録する。

- 重症度（高度、中等度、軽度）

軽度：容易に我慢でき、不快さがわずかであり日常生活に支障がない程度の有害事象

中等度：日常生活に支障を来す程度の不快な有害事象

高度：通常の日常生活が営めない程度の有害事象

- 重篤性（重篤、非重篤）
- ノイズGVSの中止の有無
- 処置の有無
- 有害事象の転帰（回復、軽快、未回復、後遺症あり、死亡、不明）

2） 不具合

治験責任（分担）医師は、不具合が発現した場合、発現した不具合に関する以下の項目について記録する。

- 健康被害発生のおそれの有無
- 不具合の内容
- 発現日時
- 発生事象の重篤性
- 予測可能性

### 重篤な有害事象の定義

重篤な有害事象とは、有害事象のうち以下のいずれかに該当するものとする。

(1) 死亡に至るもの

(2) 生命を脅かすもの

(3) 治療のため入院・入院期間の延長が必要なもの

(4) 永続的もしくは重大な障害・機能不全に陥るもの

(5) 先天異常を来すもの

(6) その他の重大な医学的事象

その他の状況、すなわち即座に生命を脅かしたり死亡や入院に至らなくとも、被験者を危機にさらしたり、上記のような結果に至らぬように処置を必要とするような重大な事象の場合には、それらも重篤とみなすべきである。

### 重篤な有害事象・不具合発現時の対応

治験期間中に重篤な有害事象もしくは重篤な有害事象が発生する恐れのある不具合（以下、不具合等という）が発現した場合、自ら治験を実施する者は、「安全性情報の取扱いに関する手順書」に従い当該不具合等情報を取り扱う。

自ら治験を実施する者は、「安全性情報の取扱いに関する手順書」に従って厚生労働大臣（以下、当局）への報告の要否等に関する判断を行う。

当局報告が必要と判断された場合には、自ら治験を実施する者は当局報告書を作成し、表4の報告期限内に、独立行政法人医薬品医療機器総合機構に提出する。

自ら治験を実施する者は「安全性情報の取扱いに関する手順書」に従って、各施設の治験責任医師及び治験機器提供者に情報共有する。

表6　重篤な有害事象の報告期限

|  | 予測できないもの | 予測できるもの |
| --- | --- | --- |
| 死亡に至るもの  生命を脅かすもの | 7日  1年毎の定期報告 | 15日  1年毎の定期報告 |
| 治療のため入院・入院期間の延長が必要なもの  永続的もしくは重大な障害・機能不全に陥るもの  先天異常を来すもの | 15日  1年毎の定期報告 | 1年毎の定期報告 |
| 重篤な有害事象につながるおそれのある不具合 | 30日  1年毎の定期報告 | |

### 予想される副作用等

これまで実施された臨床研究において報告された有害事象は脳梗塞、眩暈感及び右難聴であった。そのうち、ノイズGVSとの因果関係が否定できない事象（副作用）はなかった。機器の不具合事象としては、「刺激装置本体の電源ON/BATT LEDの高速点灯」があった。

予期される不具合

- 電気刺激に非常に敏感な場合、電気刺激による痛みを生じる可能性がある。
- 前庭神経が非常に敏感な場合、めまいや吐き気、頭痛などの症状や、起立・歩行中に転倒する可能性がある。
- アレルギー体質の場合、耳後部に貼付した表面電極に対して、じんましんや皮膚炎を生じる可能性がある。
- 電気刺激装置の不具合により、電気刺激が中断したり、プログラムした値よりも過小あるいは過大な電流が流れたりする可能性がある。
- 刺激装置本体の電源スイッチをONからOFFにし、再度ONにするまで3秒以下で行うと、LEDが電極外れの速い点滅や、常時点灯、又は非点灯になり出力が停止する可能性がある。

# データ解析と統計学的考察

集計・解析の詳細は「統計解析計画書」に記載する。本治験実施計画書に記載された解析からの変更はすべて「統計解析計画書」、「統計解析報告書」及び「総括報告書」に記載する。

本治験が途中で中止となった場合、利用可能なデータはすべて一覧表にまとめ、適切な統計解析を実施する。

## 治験デザインの検討

### 症例数の設定

本治験の目標症例数を60例（本登録50例、完了例40例）と設定する。

### 症例数設定の根拠

先行研究^6)^では、健常人、一側性末梢前庭障害患者、両側性末梢前庭障害患者の総軌跡長はそれぞれ1.69, 2.08, 2.17 cm（1秒当たり）であった。一側性末梢前庭障害患者で約19%の改善、両側性末梢前庭障害患者では約23%の改善があれば健常者と一致することから、プラセボ期と本治験機器を使用している間の差として、19%, 23%の約半分に相当する10%以上の総軌跡長の改善が見られれば臨床的な意義が存在すると考える。

自主臨床試験データを参考に、総軌跡長のデータに対して下記[1]のデータ発生を想定した下での1000回のシミュレーションにおける、Type 1エラーと検出力は下表のようになる。これより、40例の症例を集積すれば、10%の治療効果が存在するとき、約90%の検出力が確保できる。20%程度の脱落を想定し、また、15%程度の最適刺激が存在しない被験者を考慮し、目標症例数を60例（本登録50例、完了例40例）とする。

表7　症例数と検出力（%）の関係

| 治療効果^*1^ | N=30 | N=40 | N=50 |
| --- | --- | --- | --- |
| 0% ^*2^ | 4.7% | 5.2% | 4.7% |
| 5% | 31.5% | 38.2% | 46.1% |
| 7% | 55.0% | 70.6% | 78.2% |
| **10%** | **84.1%** | **91.2%** | **96.1%** |

*1プラセボ期に対するGVS期の改善率。

*2 治療効果0%の行がType1エラーを表す。名目の有意水準5%を担保していることが確認できた。

[1] 自主臨床試験データでは、刺激直後が最も改善が大きく（第Ⅱ期23%、第Ⅲ期19%）、3時間後まででも12%以上の改善率が存在した（下表参照）。自主臨床試験データよりもやや保守的に、GVS期、プラセボ期での総軌跡長の改善率の標準偏差を30%、各時点間での改善率の相関係数を0.3とし、[-80, 80]の範囲に切断した多変量切断正規分布から改善率のシミュレーションデータを発生させた。なお、3時間通して一定の治療効果を仮定し、その大きさは0%, 5%, 7%, 10%と変化させた。

表8　探索的臨床研究における総軌跡長の改善率の記述統計量、

および刺激直後の改善率と各時点の改善率との相関係数（%）の関係

|  |  | 平均 | 標準偏差 | 最小値 | 最大値 | 相関係数 |
| --- | --- | --- | --- | --- | --- | --- |
| 第2期 | |  |  |  |  |  |
|  | 刺激直後 | 23.1% | 14.7% | 3.1% | 49.0% | 1.00 |
|  | 30分後 | 17.1% | 14.1% | -14.3% | 44.3% | 0.49 |
|  | 1時間後 | 16.5% | 18.0% | -21.1% | 52.3% | 0.53 |
|  | 2時間後 | 17.1% | 15.0% | -6.3% | 41.2% | 0.30 |
|  | 3時間後 | 14.8% | 11.1% | -0.9% | 32.3% | 0.62 |
| 第3期 | |  |  |  |  |  |
|  | 刺激直後 | 19.2% | 23.6% | -25.2% | 69.5% | 1.00 |
|  | 30分後 | 17.0% | 22.7% | -22.5% | 70.0% | 0.84 |
|  | 1時間後 | 14.8% | 21.5% | -13.1% | 70.1% | 0.74 |
|  | 2時間後 | 12.6% | 25.5% | -31.9% | 61.3% | 0.70 |
|  | 3時間後 | 14.0% | 26.3% | -43.5% | 57.2% | 0.82 |

### 被験者数の再推定

本治験では、被験者数の再推定は計画していない。

## データ解析の検討

### 完了例

治験実施計画書に規定された刺激と検査をすべて実施し、事後（追加・追跡）検査を完了した被験者を治験の完了例とする。

### 解析対象集団

有効性に関する解析対象集団（Full Analysis Set; FAS）：

本登録例のうち、第Ⅰ期もしくは第Ⅱ期のいずれかに来院し治験機器を装着し、かつ有効性の評価項目が1項目でも測定されている症例の集団とする。ただし、以下に示す症例は除外する。

- GCP不遵守例：適切なインフォームド・コンセントが得られていない患者

治験実施計画書に適合した解析対象集団（Per Protocol Set; PPS）：

FASから、以下に示す症例を除外した集団とする。

- 選択基準に合致しない症例
- 除外基準に抵触した症例
- 併用禁止療法を施工した症例
- 第Ⅰ期と第Ⅱ期の両方に来院していない症例
- その他の重大なプロトコル逸脱例

安全性に関する解析対象集団（Safety Analysis Set; SAS）：

本登録例のうち、安全性の評価項目が1項目でも評価された集団とする。

解析から被験者を除外する場合は、その詳細及び理由を総括報告書中に記載する。

### 中間解析

　本治験では、中間解析は行わない。

### 有効性の解析手法

主要評価項目に対する主解析：

刺激中（ノイズGVS開始直後～3時間後）の主要評価項目（総軌跡長のベースラインからの変化率）を反応変数とし、時期（第I期、第II期）、治療機器（プラセボ、ノイズGVS）、時点、治療機器と時点の交互作用、および割付因子（スクリーニング時の総軌跡長（60秒当たり200cm未満、以上）、一側／両側前庭障害）を説明変数とし、個人の効果を変量効果とした混合効果モデルをあてはめる。刺激中を通した治療効果の最小二乗平均を求め、95%信頼区間、治療効果が0%という帰無仮説に対するP値を算出する。なお、自由度の算出にはKenward-Roger法を用いる。

　なお、感覚閾値を超えていることが判明した場合には、以降のデータの採否を症例検討会にて決定する。

上記モデルにより算出された治療機器・時点別の変化率の最小二乗平均の点推定値と95%信頼区間を求める。また、その経時推移を図示する。

主要評価項目に対する副次的解析：

1. 全時点における総軌跡長の生値、ベースラインからの変化量、変化率について、割付群、治療刺激期間、時点別に記述統計量を算出する。次に、割付群を併合して、治療機器、時点別に記述統計量を算出する。また、個人内での変化量、変化率の差について、時点ごとの記述統計量を算出し、1標本t検定を行いP値を算出する。
2. GVS期の刺激開始直後から刺激3時間後までの総軌跡長の変化率の平均値と、プラセボ期における刺激開始直後から刺激3時間後までの総軌跡長の変化率の平均値の差に対して、対応のあるt検定を行う。
3. ベイズ流の手法を用いた治療効果に関する推測：刺激中の総軌跡長の変化率を反応変数とし、割付群、治療機器、時点、治療機器と時点の交互作用、およびベースライン値を説明変数とし、個人の効果を変量効果とした混合効果モデルをあてはめ、治療効果に関する最小二乗平均の事後分布を得る。事前分布はJeffreysの無情報事前分布とし、GVS期の改善がプラセボ期の改善に少しでも勝る、5%、10%、15%以上勝る事後確率を計算する。また、事後確率の95%信用区間、90%信用区間を算出する。
4. GVS期における刺激中の総軌跡長の変化率を反応変数とし、ベースライン値を説明変数とした混合効果モデルをあてはめ、時点を平均した変化率の最小二乗平均の事後分布を得る。事前分布はJeffreysの無情報事前分布、自主臨床試験3の第2期刺激3時間後までのデータから得られる事前分布に対して、GVS期の改善が少なくとも存在する、5%、10%、15%、20%以上となる事後確率を計算する。また、事後確率の95%信用区間、90%信用区間を算出する。
5. 症例数設計におけるシミュレーション実験の設定の妥当性を確認するため、刺激中の総軌跡長の変化率の間の相関係数を、治療機器、時点別に算出する。
6. 刺激中の総軌跡長のベースラインからの変化量に対して、主要評価項目に対する主解析と同様のモデルを用いて解析する。
7. 主要評価項目の主解析および副次的解析①、②と同様の解析を、感覚閾値を超えていることが判明した以降のデータも利用して行う。

副次評価項目に対する解析：

- 重心動揺検査に対する解析

1. 全時点における外周面積、RMS値のベースラインからの変化率について、割付群、治療刺激期間、時点別に記述統計量を算出する。次に、割付群を併合して、治療機器、時点別に記述統計量を算出する。また、個人内での変化率の差について、時点ごとの記述統計量を算出し、1標本t検定を行いP値を算出する。
2. 全時点における総軌跡長、外周面積、RMS値の変化率に対して、治療機器・時点別の変化率の平均値と95%信頼区間の経時推移を図示する。

- 歩行機能検査に対する解析

1. 全時点における歩行検査の評価項目（DGIスコア、10メートル歩行時の歩行速度、歩幅、ステップ時間と左右の揺れ幅）のベースラインからの変化率について、割付群、治療刺激期間、時点別に記述統計量を算出する。次に、割付群を併合して、治療機器、時点別に記述統計量を算出する。また、時点ごとの個人内での変化率の差について、全体および群別に時点ごとの記述統計量を算出し、群間のt検定を行いP値を算出する。
2. 刺激中の外周面積、RMS値、歩行検査の評価項目（DGIスコア、10メートル歩行時の歩行速度、歩幅、ステップ時間と左右の揺れ幅）の変化率に対して、主要評価項目に対する主解析と同様のモデルを用いて解析する。
3. 全時点における歩行検査の評価項目の変化率に対して、治療機器・時点別の変化率の平均値と95%信頼区間の経時推移を図示する。

- 自覚的改善度スコアに対する解析

1. 全時点における自覚的改善度スコアについて、割付群、治療刺激期間、時点別に記述統計量を算出する。次に、割付群を併合して、治療機器、時点別に記述統計量を算出する。
2. 刺激中の自覚的改善度スコアを反応変数とし、割付群、治療機器、時点、治療機器と時点の交互作用を説明変数とし、個人の効果を変量効果とした混合効果モデルをあてはめる。刺激中を通した治療効果の最小二乗平均を求め、95%信頼区間、P値を算出する。なお、自由度の算出にはKenward-Roger法を用いる。
3. 全時点における自覚的改善度スコアに対して、治療機器・時点別の平均値と95%信頼区間の経時推移を図示する。

- QOLデータに対する解析

1. 全時点におけるmFESスコア、DHIスコア、DHI各サブスケールのスコアのベースラインからの変化量について、割付群、治療刺激期間、時点別に記述統計量を算出する。次に、割付群を併合して、治療機器、時点別に記述統計量を算出する。また、個人内での変化量の差について、時点ごとの記述統計量を算出し、1標本t検定を行いP値を算出する。
2. 刺激開始3時間後、および7時間後のそれぞれのmFESスコア、DHIスコア、DHI各サブスケールのスコアを応答変数とし、割付群、治療機器、ベースライン値を説明変数とした共分散分析を行う。

- 評価項目間の相関の検討

1. 刺激中の各時点における総軌跡長の変化率と、以下の変数の間でのPearson/Spearmanの相関係数を、治療機器別に算出する。

・ 歩行検査の評価項目の変化率

・ 自覚的改善度スコア

1. 刺激開始3時間後、および7時間後それぞれの時点で、総軌跡長の変化率と、以下の変数の間でのPearson/Spearmanの相関係数を、治療機器別に算出する。

・ mFESスコア

・ DHIスコア

・ DHI各サブスケールのスコア

・ 歩行数

- 事後確率の算出

1. それぞれの副次評価項目について、主要評価項目の副次的解析③と同様にベイズ流の手法を用いて、事前分布をJeffreysの無情報事前分布とし、GVS期の改善がプラセボ期の改善に少しでも勝る、5%を上回る（自覚的改善度スコアでは0点、1点）事後確率、その95%信用区間を算出する。

### 安全性の解析手法

　安全性の解析には安全性解析対象集団を用いる。安全性データの解析ではデータの集計表及び一覧表を作成し、統計学的な検定は行わない。

#### 有害事象

　有害事象は、ICH国際医薬用語集（MedDRA）によりコード化し、器官別大分類（SOC）及び基本語（PT）別に分類し、刺激別に集計を行う。

　死亡、重篤、中止に至った有害事象及びその他の重要な有害事象が認められた場合は、一覧表を別途作成する。

#### 治験機器の不具合

　治験機器の不具合について事象別に集計を行う。

# 治験の品質管理及び品質保証

## 同意取得手順を含む、規制及び倫理上の考慮事項

　本治験は、医療機器の臨床試験の実施に関する基準（GCP）、薬機法第14条の3項及び第80条の2に定める基準、適用されるすべての被験者のプライバシー保護の要件、及びヘルシンキ宣言（最新改定を含む）を遵守して実施する。これには次の項目を含むが、これに限定されるものではない。

治験審査委員会（IRB）による治験実施計画書とその後の改訂の検討及び承認

被験者の同意

治験責任医師の報告要件

## 治験計画の規制当局への届出

　自ら治験を実施する者は、薬器法第80条の2に従って、規制当局に治験の計画届を提出する。

## 治験実施計画の逸脱及び変更

### 治験実施計画書からの逸脱

　被験者の緊急の危険を回避するために、治験責任（分担）医師は、自ら治験を実施する者との事前の文書による合意及び治験審査委員会の事前の承認なしに治験実施計画書から逸脱することができる｡このような逸脱を行った場合には、治験責任医師はすべての逸脱の内容及び理由を記録し、速やかに実施医療機関の長及び治験審査委員会の承認を得るとともに実施医療機関の長を経由して自ら治験を実施する者の合意を文書で得る。

　また、治験責任（分担）医師は、治験実施計画書から逸脱した行為をすべて記録する。治験責任医師は、被験者の緊急の危険を回避するために行った逸脱についてのみ、その理由等を説明した記録を作成して自ら治験を実施する者及び実施医療機関の長に提出し、その写しを保存する。

### 治験実施計画書の変更

1. 治験実施計画の重大な変更（被験者に対する危険を増大させるか又は治験の実施に影響を及ぼす事項に関する変更など）が必要となった場合、自ら治験を実施する者（治験調整医師）は、治験責任医師と協議のもとに速やかにすべての変更内容及び理由を文書で記録するとともに治験実施計画書を改訂し、実施医療機関の長及び治験責任医師にその治験実施計画書（必要に応じ同意文書及びその他の説明文書見本）の変更について通知する。治験責任医師は、治験審査委員会の承認を得る前は重大な変更を実施しない。
2. 上記1）に該当しない変更が必要となった場合、自ら治験を実施する者（治験調整医師）は治験責任医師との協議のもとに、すべての変更内容及び理由を文書にて記録し、実施医療機関の長及び治験責任医師に通知する。緊急の危険の回避の場合を除き、これらの変更も事前にすべて治験審査委員会の承認を得なければならない。

## 品質管理

自ら治験を実施する者は、治験実施に関する標準業務手順書、治験に先立ち定めた標準業務手順書及び本治験におけるモニタリングの実施に関する手順書に従い、治験の品質管理を行う。

### モニタリング

　自ら治験を実施する者は，当該治験がヘルシンキ宣言の精神に基づいて実施され，薬機法GCP及び治験実施計画書を遵守して行われていること、並びに治験データ等が正確かつ完全で，原資料等の治験関連記録に照らして検証できることを確認するため、モニタリングを実施させる。自ら治験を実施する者は、モニターの要件を満たすことを確認した上で当該治験のモニターとして指名する。

モニターは実施医療機関、治験責任医師、治験分担医師、治験協力者、治験機器管理者など、治験実施関係者に対するモニタリングにより以下のことを行う。

1. 被験者の同意取得状況、治験機器の管理状況、治験の進捗状況（中止、有害事象を含む）などを調査し、治験がGCP、本治験実施計画書及び自ら治験を実施する者と実施医療機関、治験責任医師との間のその他の合意文書に基づいて行われていることを確認する。
2. 適切に治験を実施するために必要な情報（治験機器に関する安全性・有効性・品質などに関する情報）の収集と提供を行う。
3. 実施医療機関及び治験責任医師が治験を適切に実施するのに求められる要件を満たし、それが治験期間を通して維持していることを確認する。また、検査室や必要な装置及びスタッフを含む設備が、治験を安全かつ適正に実施するのに十分であり、それが治験期間を通して継続していることを確認する。
4. 正確かつ完全で、最新に至る原資料などのすべての治験関連記録が作成、保存されていることを確認する。
5. 実施医療機関において保存すべき必須文書をそれぞれの保管責任者が保存していることを確認する。
6. 症例報告書の内容と原資料などのすべての治験関連記録を相互に照合し、これらが正確であることを確認する。

　治験責任医師及び実施医療機関は、モニターによる本治験に関連する原資料などの直接閲覧に応じなければならない。

　モニターによる直接閲覧及び原資料の特定については、別途当該治験に特有の「モニタリング手順書」に定める。

### データマネジメント

データの品質管理は自ら治験を実施する者が治験に先立ち定めた標準業務手順書に従い、モニタリング、データマネジメントによって行われる。

データマネジメントは、治験実施計画書にもっとも有効なデータ収集及びマネジメント方法を特定して実施し、治験実施計画書の目的に応じたデータセットを提示する。

被験者データを自ら治験を実施する者が指定する電子症例報告書に入力し、自ら治験を実施する者に電子的に送信し、バリデートされたデータマネジメントシステムで他のデータと合わせる。

データマネジメントは、適用されるデータクリーニングの手順に従って行われ、データの誤り及び矛盾を取り除くことなどデータの完全性を確保することを目的としている。有害事象用語は、最新版のMedDRA（ICH国際医薬用語集）を、併用薬剤名は、医薬品名データファイルをそれぞれ用いてコード化する。

電子症例報告書（クエリ、監査証跡を含む）は自ら治験を実施する者が保管し、写しは治験責任医師に送付され、治験責任医師の写しとして保管する。いかなる場合でも、被験者のイニシャルは収集されず、自ら治験を実施する者に報告されない。

また、治験参加に不適格と判断された被験者など、治験機器を使用しなかった被験者の症例報告書は作成しない。

## 品質保証

　自ら治験を実施する者は、GCP、適用されるすべての規制要件、治験実施計画書及び治験の実施に係る手順書を遵守して、治験の実施並びにデータの作成、記録及び報告が行われていることを保証するために、モニタリングを担当する部門を含む治験に係る部門からは独立した者が監査を行わせる。

また、治験審査委員会及び規制当局は、治験期間中又は治験終了後、随時、調査を行うことがある。

監査又は調査が行われる場合、自ら治験を実施する者、治験責任医師（及び実施医療機関の長）は、監査担当者及び調査官がすべての関連文書を直接閲覧できること、また、治験責任（分担）医師及び治験協力者などが、適切な監査及び調査の実施に協力することに同意する。

## 実施医療機関における治験の中止

　自ら治験を実施する者は、安全性又は倫理上の問題、あるいは重大な不遵守を含む理由により、随時、治験を一時的に中断又は早期に中止することができる。自ら治験を実施する者がそのような措置が必要であると判断した場合、治験の中断・中止及びその理由に関して治験責任医師及び医療機関の長（該当する場合）と協議する。可能な場合には、自ら治験を実施する者は治験を中断・中止する前に、治験責任医師又は医療機関の長にその旨を報告する。

　治験を安全性の理由で中断又は中止する場合、自ら治験を実施する者は直ちにすべての治験責任医師、医療機関の長（該当する場合）、及び／又は医療機関に報告する。さらに、自ら治験を実施する者は、規制当局に対して、直ちに治験の中断・中止及びその理由を報告する。適用される規制で定められている場合、治験責任医師又は医療機関の長は直ちにIRBに報告し、中断・中止の理由を説明する。

## 記録の保存

　治験終了後、治験責任医師又は医療機関の長は、すべての治験記録を安全な場所で保存しなければならない。この記録は、自ら治験を実施する者の監査又は規制当局の調査等の場合に容易に取り出すことができなければならない。

　自ら治験を実施する者は、適用されるすべての規制要件を遵守するため、医療機関の記録を保存する期間を治験責任医師に通知する。最低保存期間は、各国の法律／規制、自ら治験を実施する者の標準業務手順書、及び／又は実施医療機関の要件のうち、原則として実施医療機関に適用されるもっとも長い基準に従うものとする。

　治験責任医師は、実施医療機関以外の施設における記録の長期保存、又は治験責任医師の転出に伴う記録の所有権の譲渡など、長期保存に関する合意を自ら治験を実施する者に通知する。

　本治験に関する被験者診療記録、検査データ、治験審査委員会の記録、契約書、被験者の同意に関する記録、治験機器管理記録等、GCPにおいて実施医療機関にて保管することが義務付けられている資料について、実施医療機関の定める保管責任者は次の1）又は2）の日のうち後の日までの間保存しなければならない。ただし、自ら治験を実施する者がこれよりも長期間の保存を必要とする場合には、保存期間及び保存方法について自ら治験を実施する者と協議するものとする。記録の保存に際しては、それぞれの記録ごとに記録の保管責任者を定めて保存するものとする。なお、保存の必要がなくなった場合には、自ら治験を実施する者から実施医療機関の長へ書面による通知がなされる。

1. 当該治験機器にかかる製造販売承認日から5年が経過した日（開発を中止した又は臨床試験の試験成績に関する資料が申請書に添付されないことを決定した旨の通知をした日から3年が経過した日）
2. 治験の中止又は終了後3年が経過した日

## 治験責任医師への治験結果及び情報の提供

　適用される規制要件で求められている場合、治験責任医師は署名により治験の総括報告書を承認する。治験責任医師には、統計図表や関連する報告書を適宜入手し、自ら治験を実施する者又は相互に合意された場所で、治験全体の結果を検討する機会を提供する。

　総括報告書の完成後、自ら治験を実施する者はJAPIC等を通じて治験の結果を公開し、治験結果の全要約を治験責任医師に提供する。治験責任医師は、適宜、治験結果の要約を被験者に知らせる。

## 治験実施期間

2018年12月から2022年6月まで（最初の被験者の同意取得日から最後の被験者の治験終了まで）

# 治験実施体制

別紙のとおり。

# 引用文献

1. Neuhauser HK, von Brevern M, Lezius F, Fedomannn M, Ziese T, Lempert T. Epidemiology of vestibular vertigo: A neurotologic survey of the general population. Neulology 2005; 65: 898-904.
2. Agrawal Y, Carey JP, Della Santina CC, Schubert MC, Minor LB. Disorders of balance and vetibular function in US adults: Data from the National Health and Nutritional Examination Survey, 2001-2004. Arch Intern Med 2009; 169: 938-944.
3. Mulavara AP, Fiedler MJ, Kofman IS, Wood SJ, Serrador JM, Peters B, Cohen HS, Reschke MF, Bloomberg JJ. Improving balance function using vestibular stochastic resonance: optimizing stimulus characteristics. Exp Brain Res 2011; 210: 303-312
4. Flores A, Manilla S, Huidobro N, De La Torre-Valdovinos B, Kristeva R, Mendez-Balbuena I, Galindo F, Trevin˜o M, Manjarrez E. Stochastic resonance in the synaptic transmission between hair cells and vestibular primary afferents in development. Neuroscience 2016; 322: 416–429.
5. Iwasaki S, Yamamoto Y, Togo F, Kinoshita M, Yoshifuji Y, Fujimoto C, Yamasoba T. Noisy vestibular stimulation improves body balance in bilateral vestibulopathy. Neurology 2014; 82: 969-975
6. Fujimoto C, Murofushi T, Chihara Y, Ushio M, Sugasawa K, Yamaguchi T, Yamasoba T, Iwasaki S. Assessment of diagnostic accuracy of foam posturography for peripheral vestibular disorders: Analysis of parameters related to visual and somatosensory dependence. Clinical Neurophysiol 2009; 120: 1408-1414.
7. 東京大学医学部附属病院資料
8. 東京大学医学部附属病院試験総括報告書
9. Shumway-Cook A, Gruber W, Baldwin M, Liao S. The effect of multidimensional exercises on balance, mobility, and fall risk in community-dwelling older adults. Phys Ther. 1997; 77: 46-57.
10. R.GoelaM.J.RosenbergbH.S.Cohenb, et.al. Calibrating balance perturbation using electrical stimulation of the vestibular system. J Neurosci Methods. 2018; 311:193-199
11. Nakamura J, Kita Y, Ikuno K, et. al. Influence of the stimulus parameters of galvanic vestibular stimulation on unilateral spatial neglect. Neuroreport. 2015; 26: 462-466.
12. Aw ST, Todd MJ, Halmagyi GM. Latency and initiation of the human vestibuloocular reflex to pulsed galvanic stimulation. J Neurophysiol. 2006; 96: 925-30.

# 別添1　Dynamic Gait Index

# 別添2　日本語版modified Fall Efficacy Scale


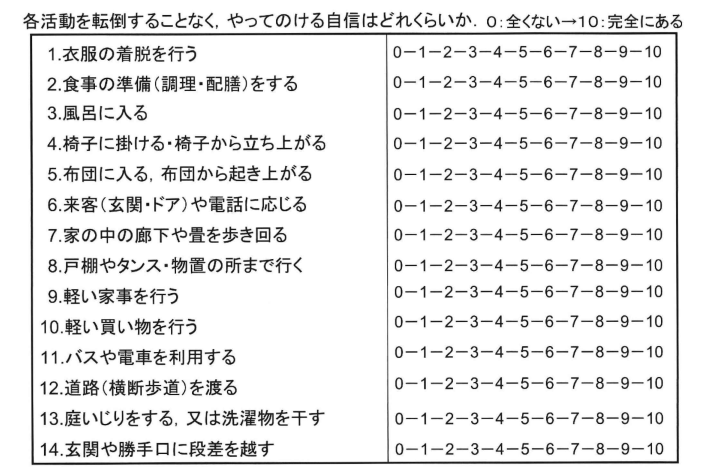


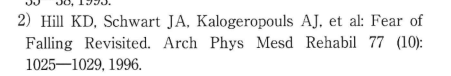


# 別添3　日本語版Dizziness Handicap Inventory


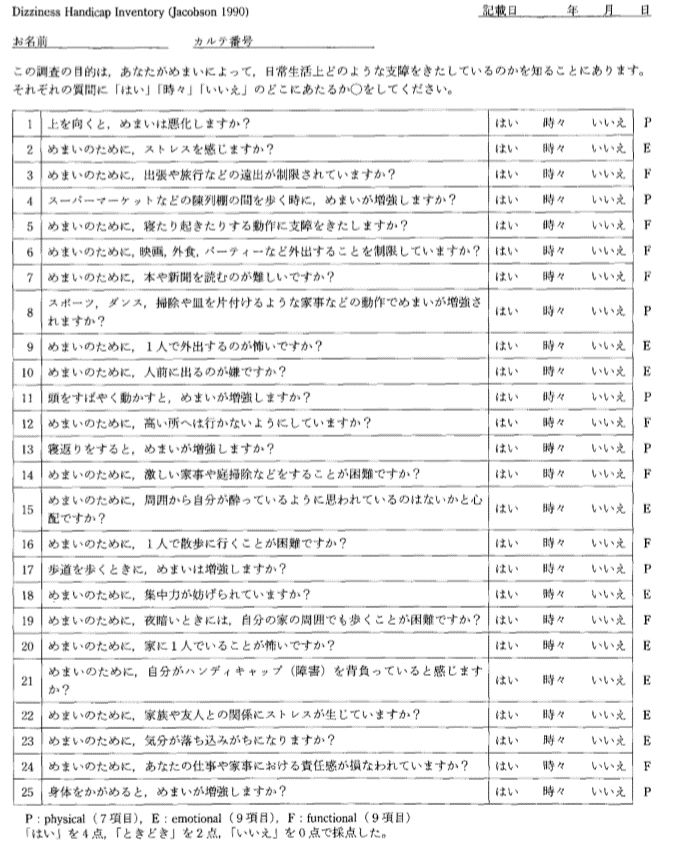


増田佳奈子，五島史行，藤井正人，他：めまいの問診表（和訳Dizziness　Handicap　Inven－ tory）の有用性の検討．　Equilibrium　Res　63：555－563，2004
