# Supplementary material for: Multicenter randomized double-blind placebo-controlled crossover study of the effect of prolonged noisy galvanic vestibular stimulation on posture or gait in vestibulopathy
Source: PLoS One. 2025 Jan 24;20(1):e0317822. doi: 10.1371/journal.pone.0317822 (PMC11760040; doi:10.1371/journal.pone.0317822)
Supplement: S3 File — (DOCX) [file pone.0317822.s003.docx]

## Supplementary Figures


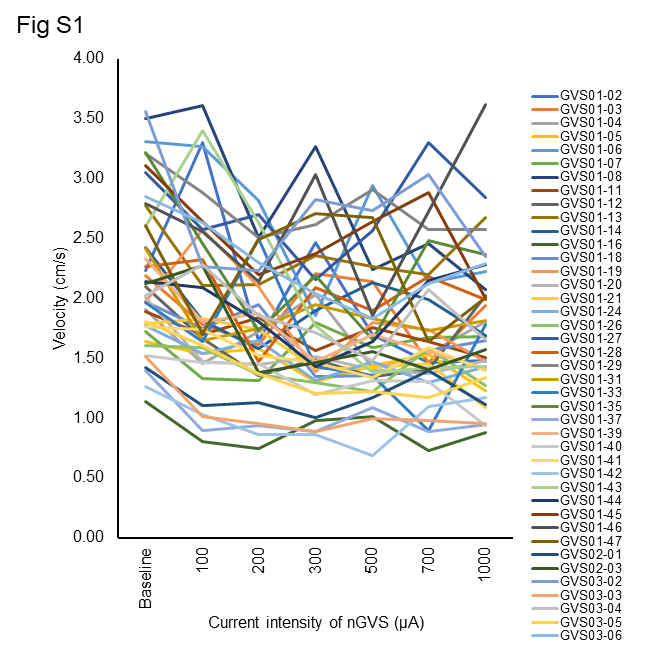


**Fig S1 Velocity of COP movement at applied nGVS current intensity for each enrolled subject when measuring optimal intensity.** nGVS = noisy galvanic vestibular stimulation

**
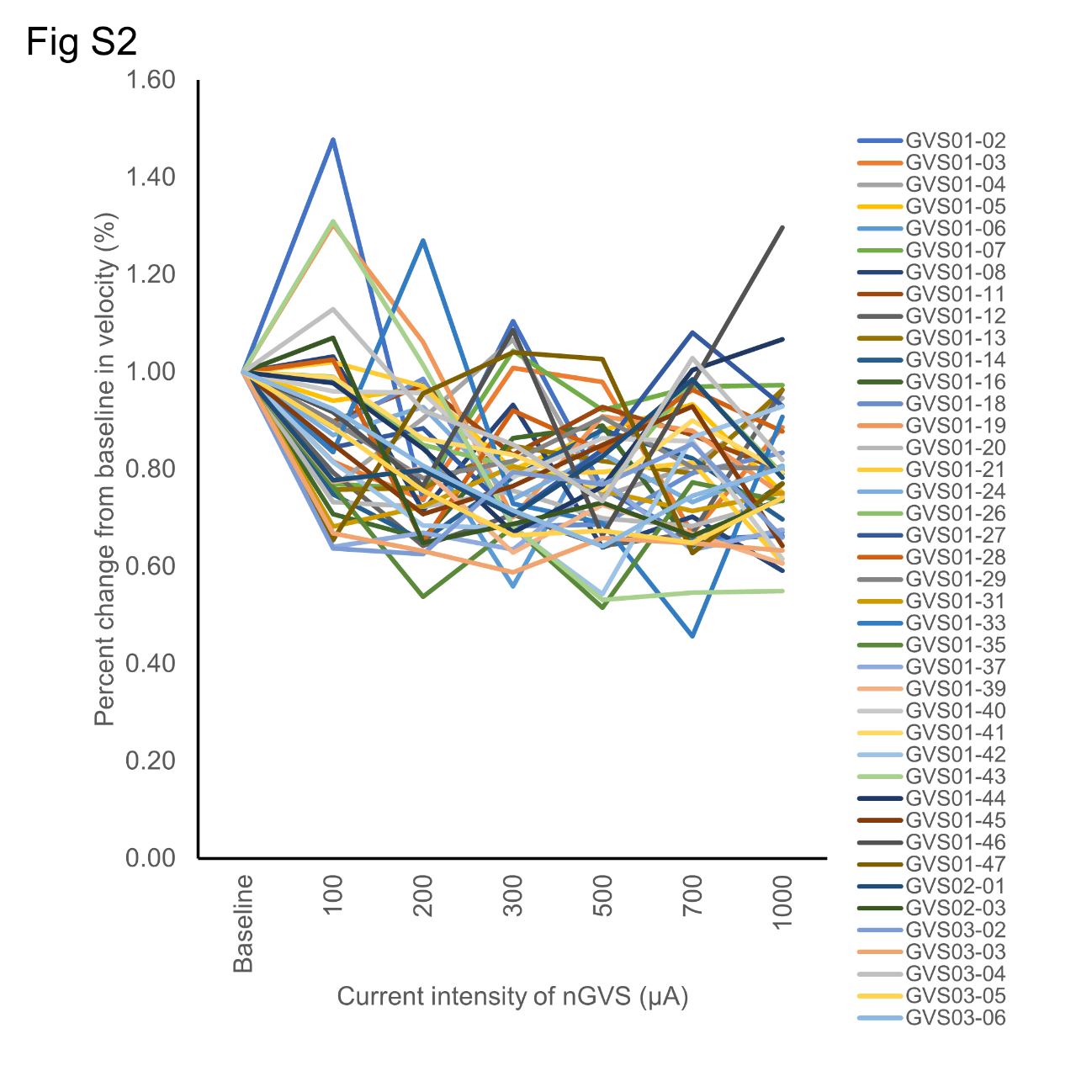
**

**Fig S2 Percent change from the baseline in velocity of COP movement at applied nGVS current intensity for each enrolled subject when measuring optimal intensity.** nGVS = noisy galvanic vestibular stimulation


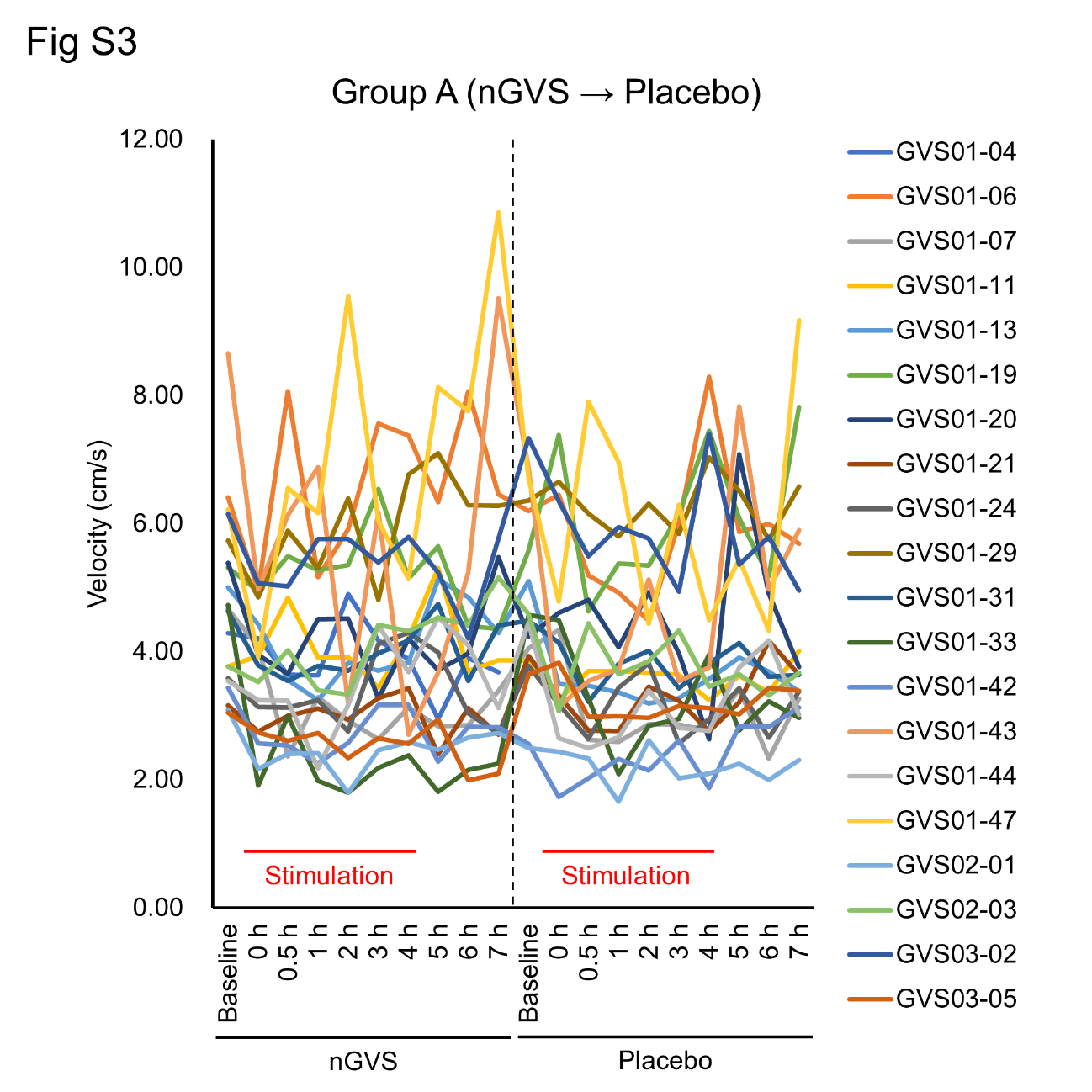


**Fig S3 Velocity of COP movement for each enrolled subject in Group A.** nGVS = noisy galvanic vestibular stimulation


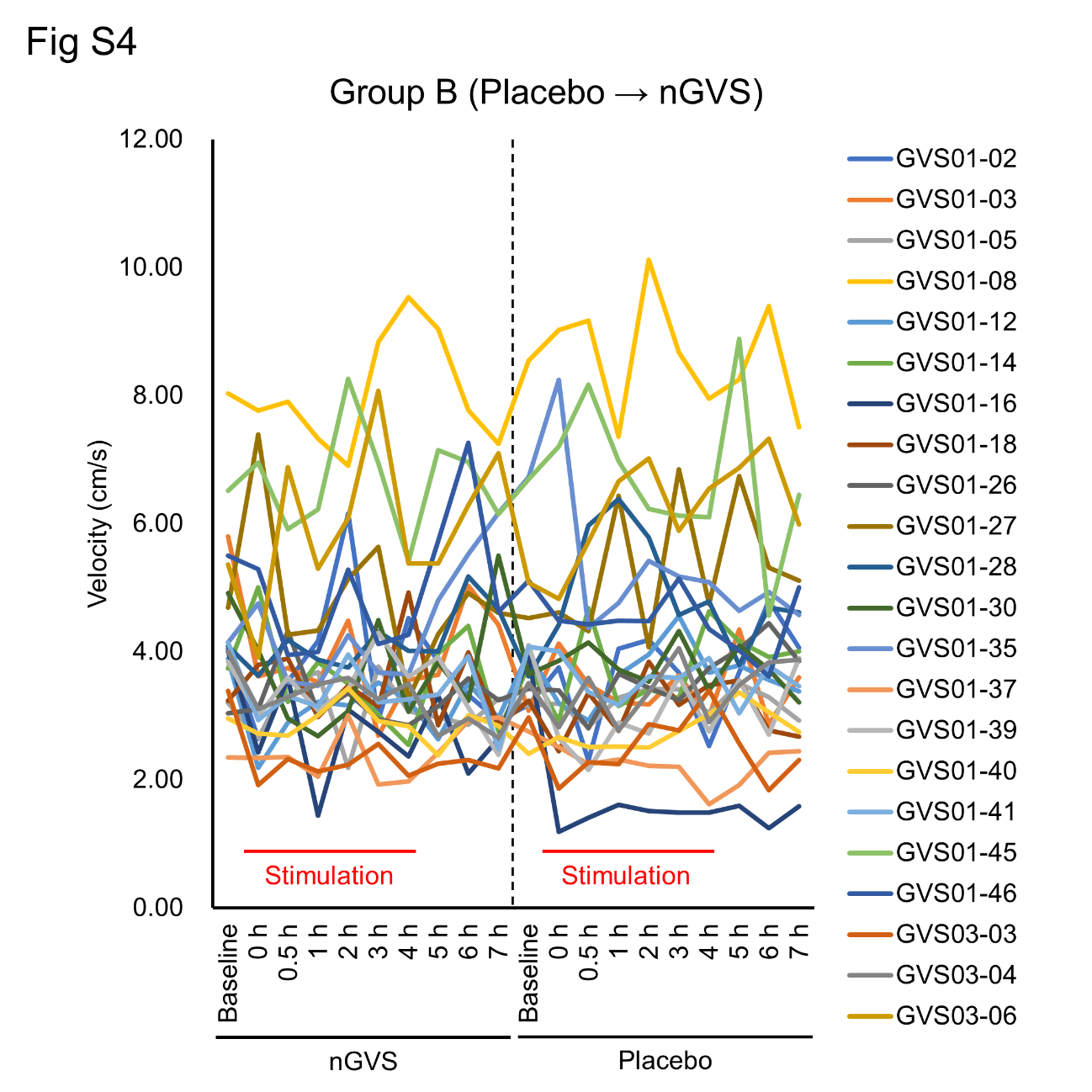


**Fig S4 Velocity of COP movement for each enrolled subject in Group B.** nGVS = noisy galvanic vestibular stimulation


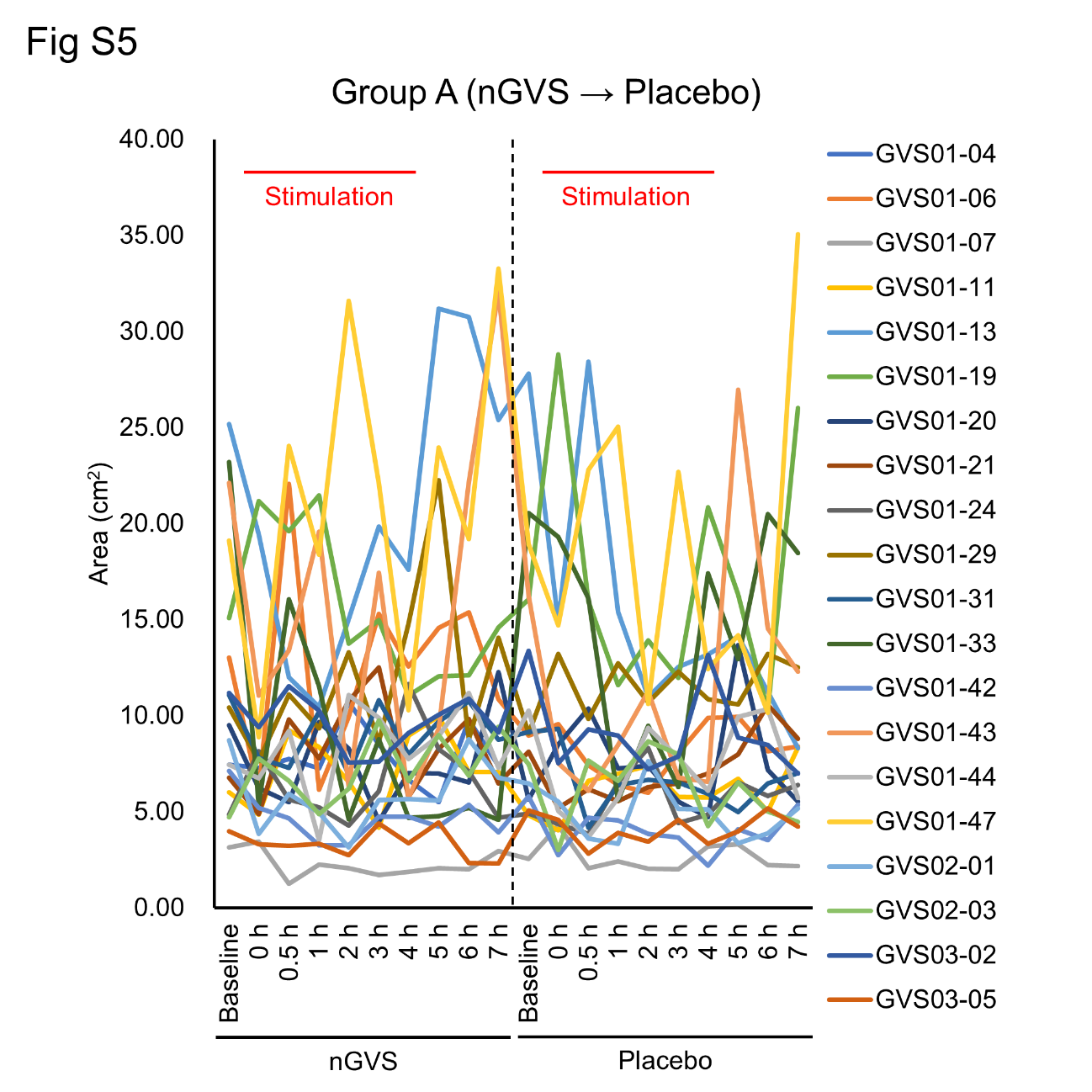


**Fig S5 Area of COP movement for each enrolled subject in Group A.** nGVS = noisy galvanic vestibular stimulation


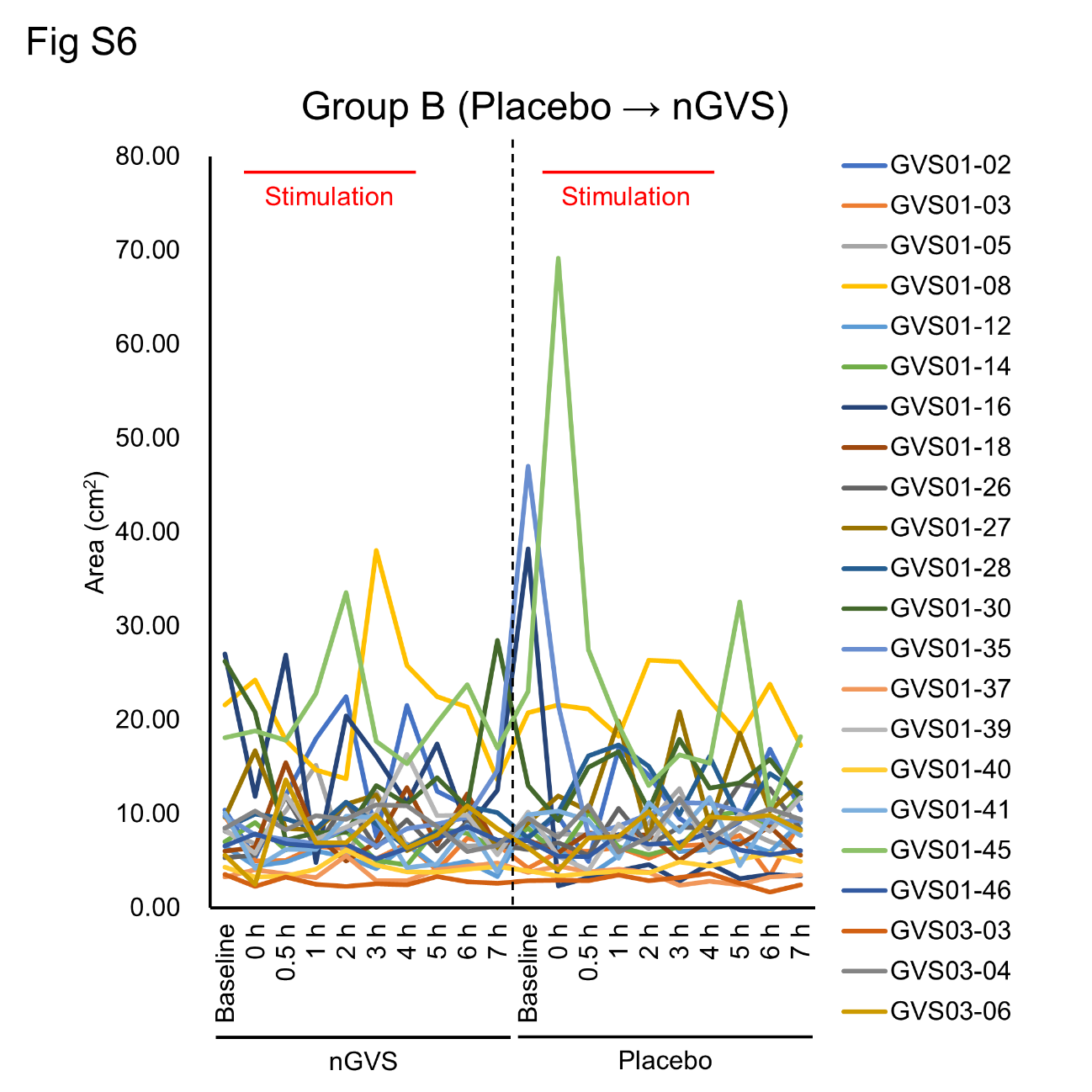


**Fig S6 Area of COP movement for each enrolled subject in Group B.** nGVS = noisy galvanic vestibular stimulation


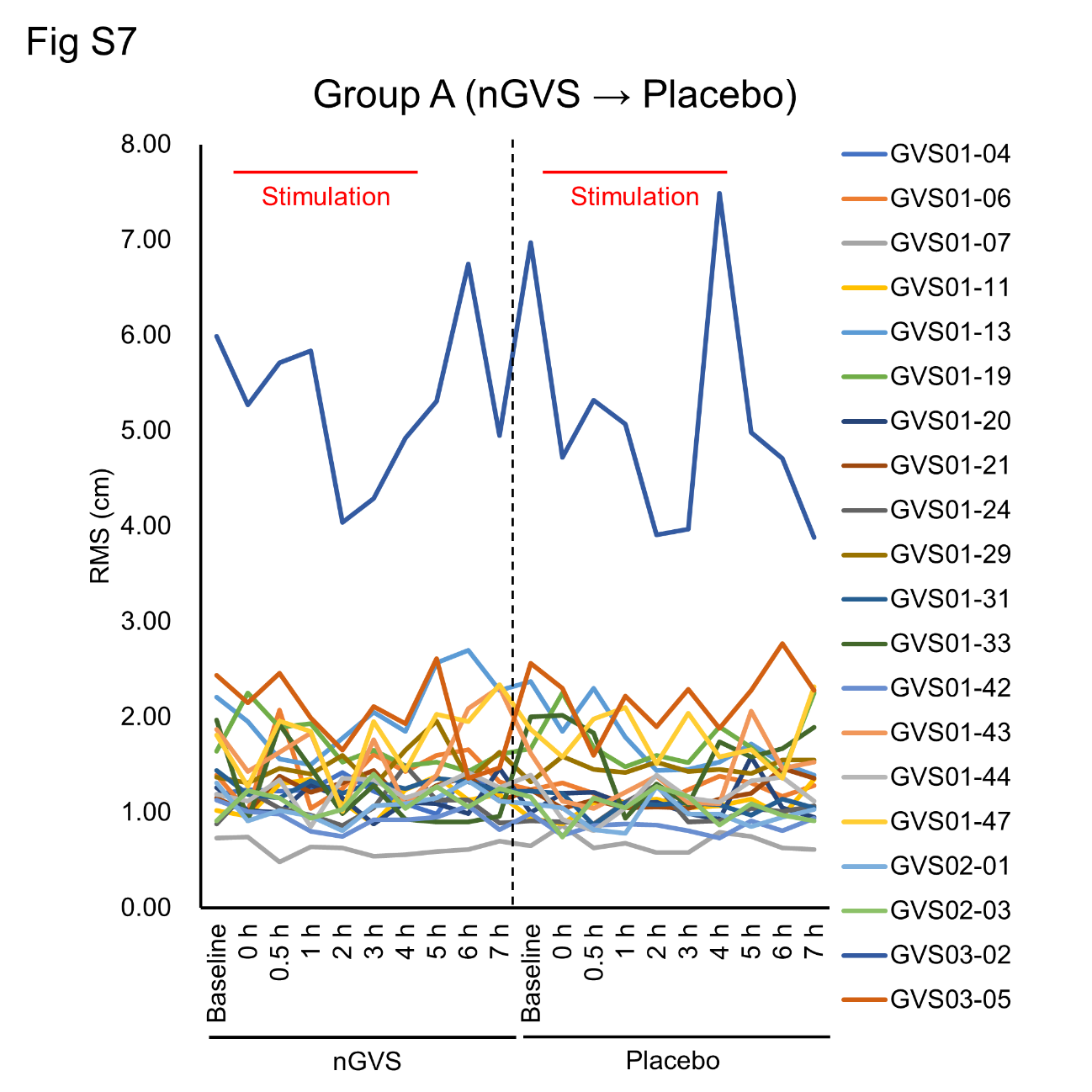


**Fig S7 RMS of COP movement for each enrolled subject in Group A.** nGVS = noisy galvanic vestibular stimulation, RMS = root mean square


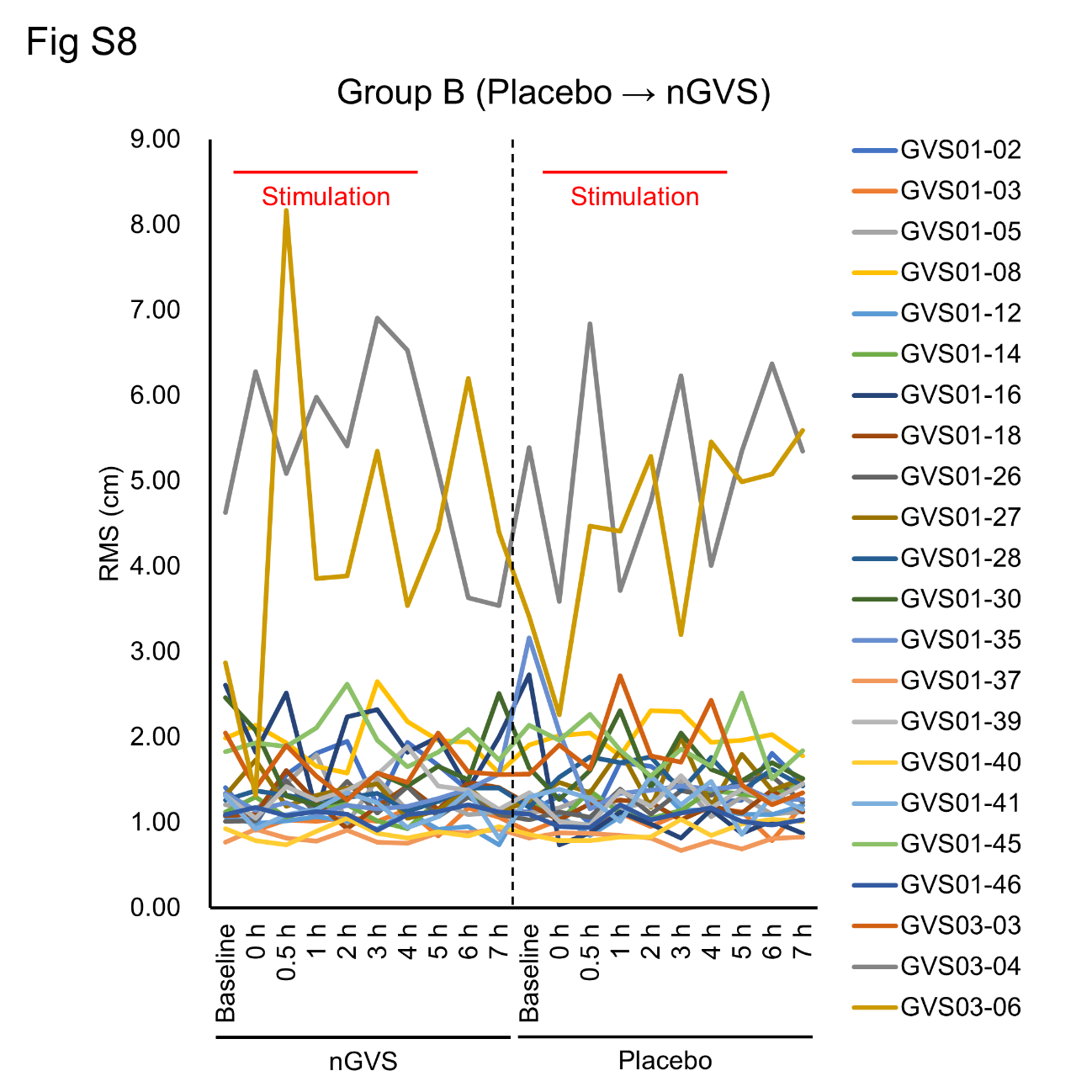


**Fig S8 RMS of COP movement for each enrolled subject in Group B.** nGVS = noisy galvanic vestibular stimulation, RMS = root mean square


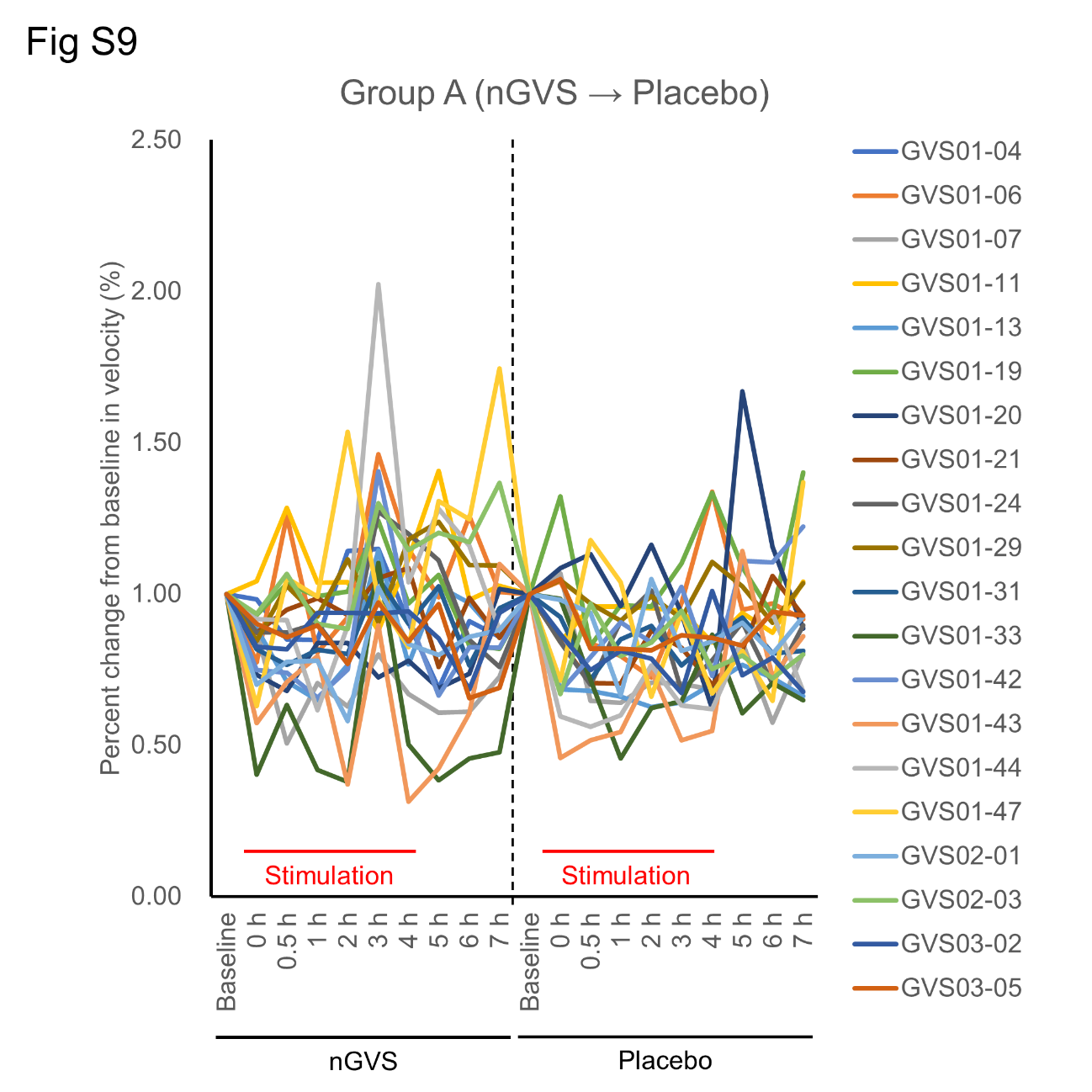


**Fig S9 Percent change from the baseline in velocity of COP movement for each enrolled subject in Group A.** nGVS = noisy galvanic vestibular stimulation


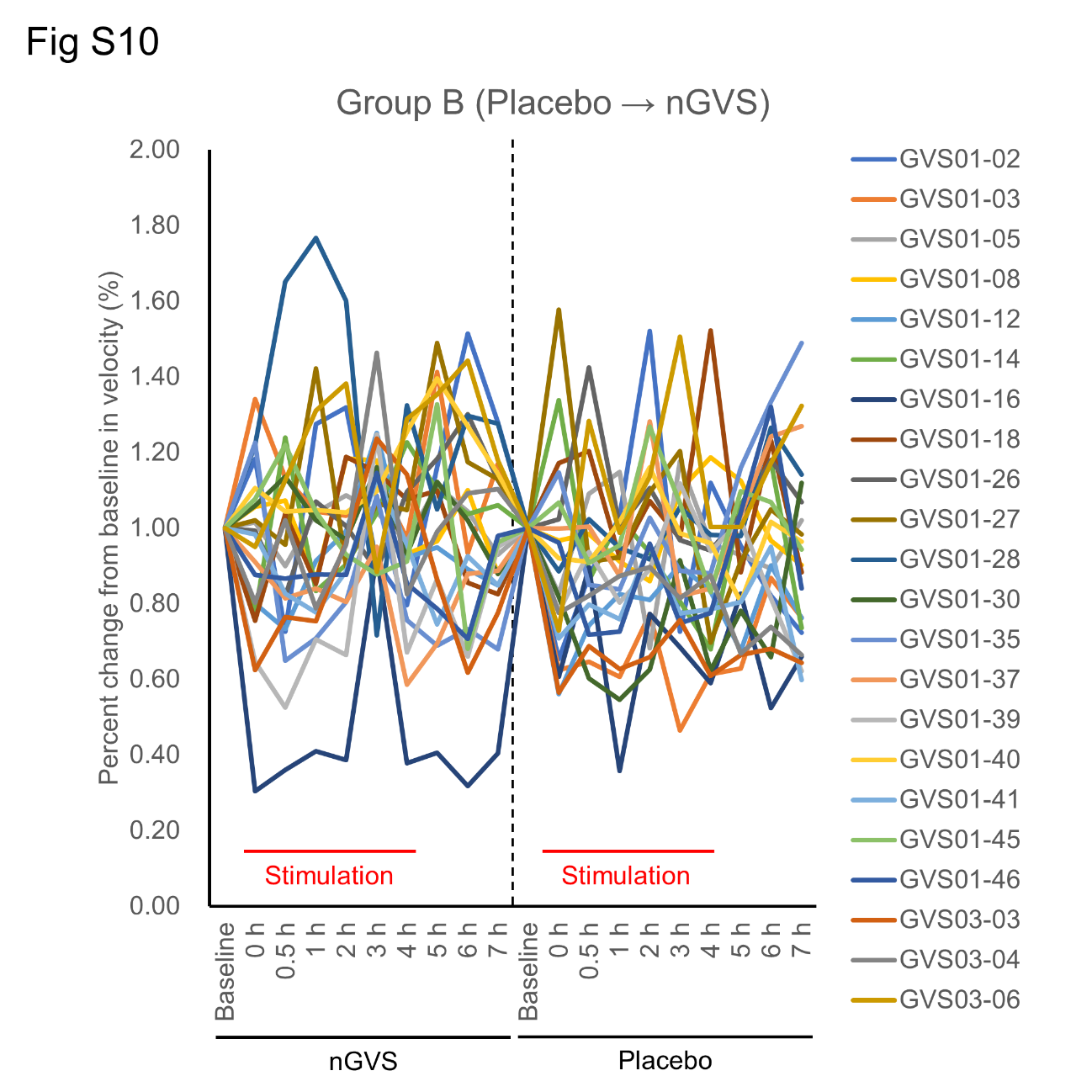


**Fig S10 Percent change from the baseline in velocity of COP movement for each enrolled subject in Group B.** nGVS = noisy galvanic vestibular stimulation


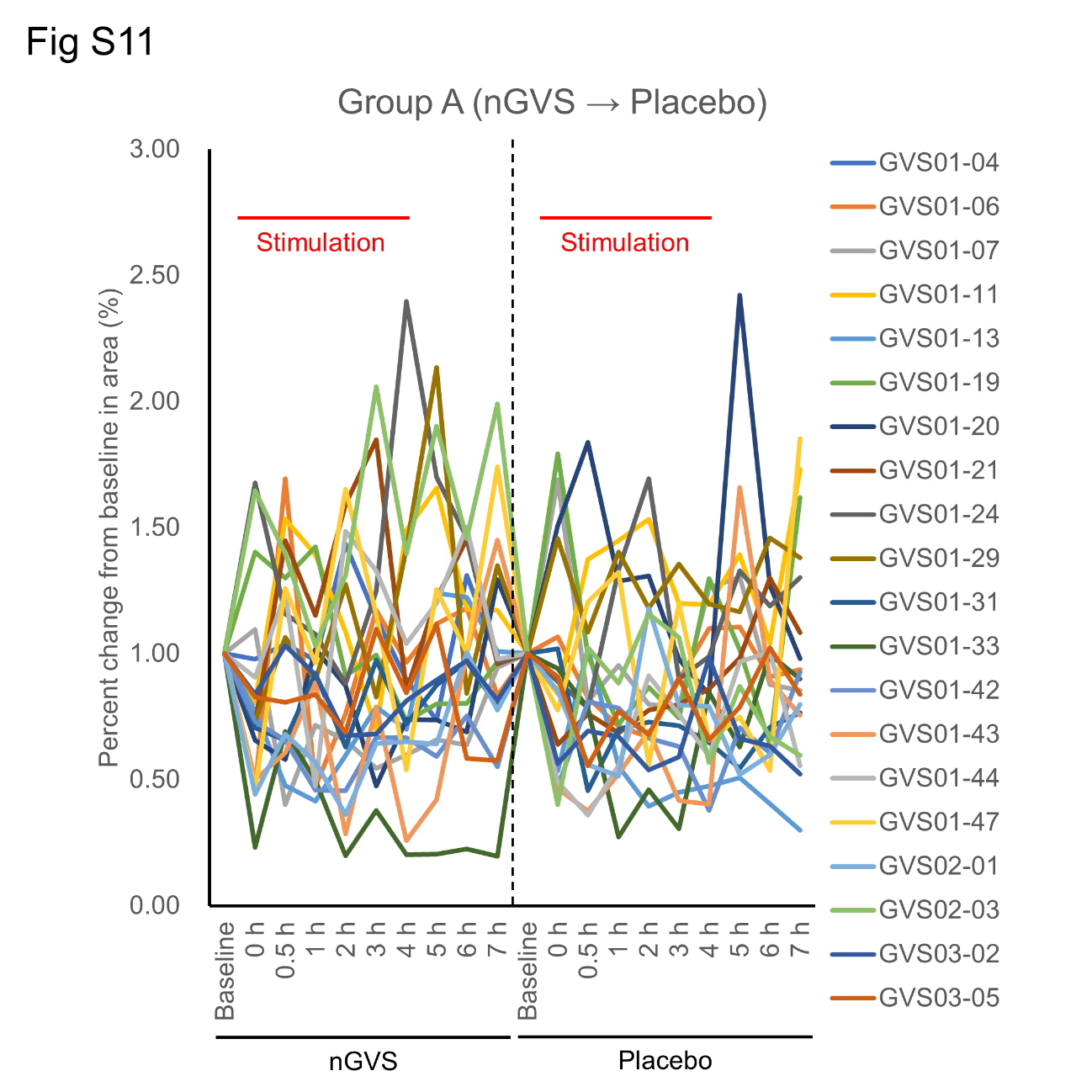


**Fig S11 Percent change from the baseline in area of COP movement for each enrolled subject in Group A.** nGVS = noisy galvanic vestibular stimulation


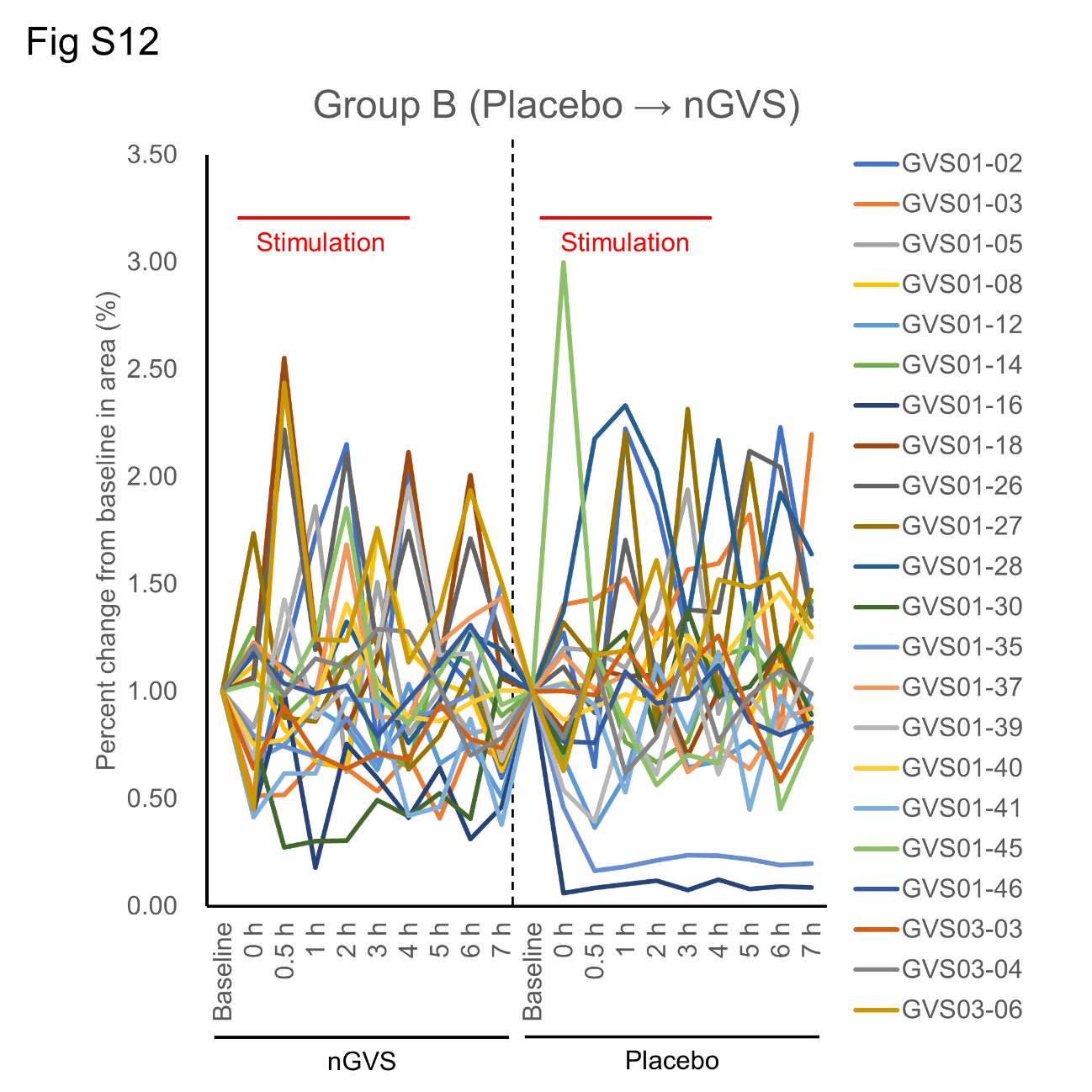


**Fig S12 Percent change from the baseline in area of COP movement for each enrolled subject in Group B.** nGVS = noisy galvanic vestibular stimulation


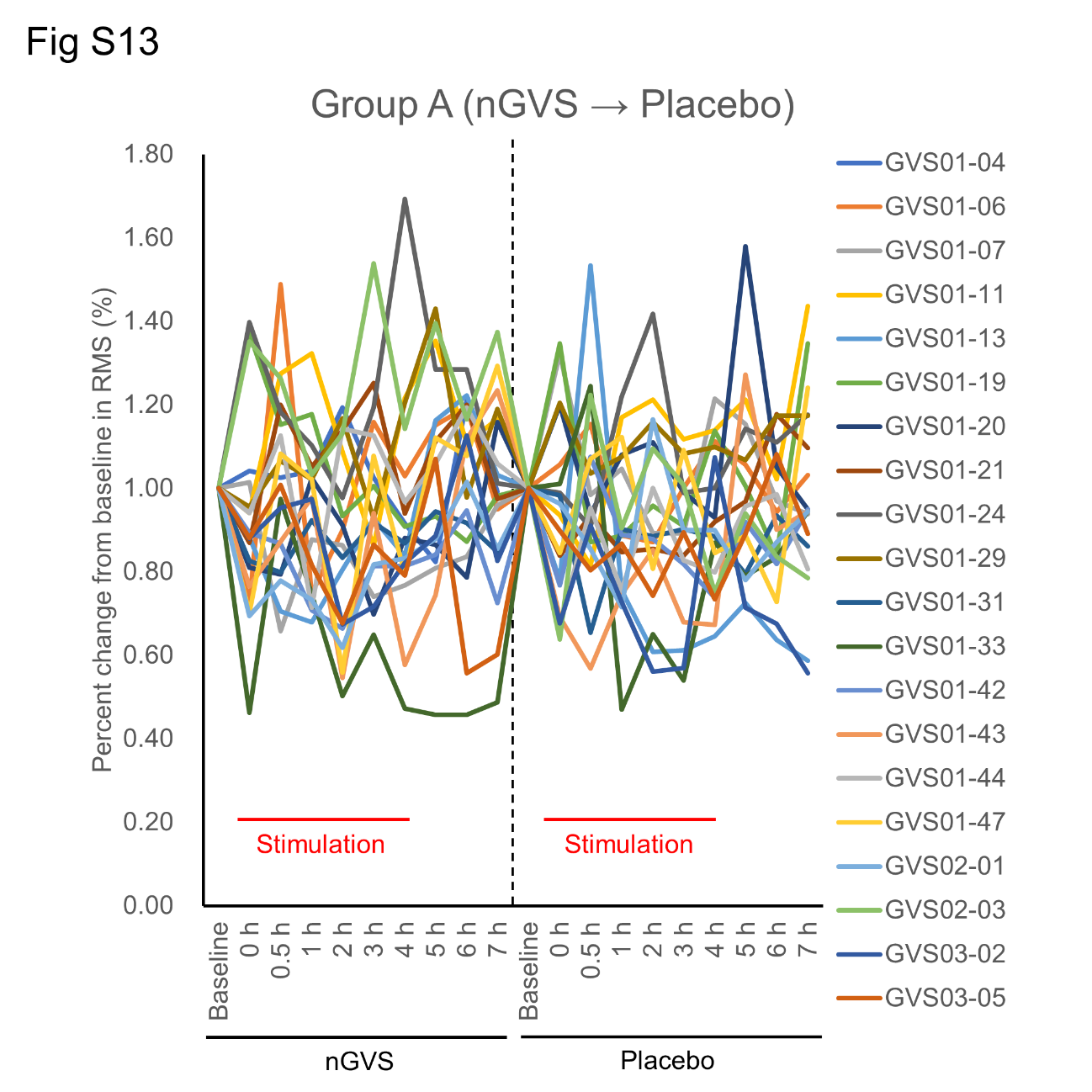


**Fig S13 Percent change from the baseline in RMS of COP movement for each enrolled subject in Group A.** nGVS = noisy galvanic vestibular stimulation, RMS = root mean square


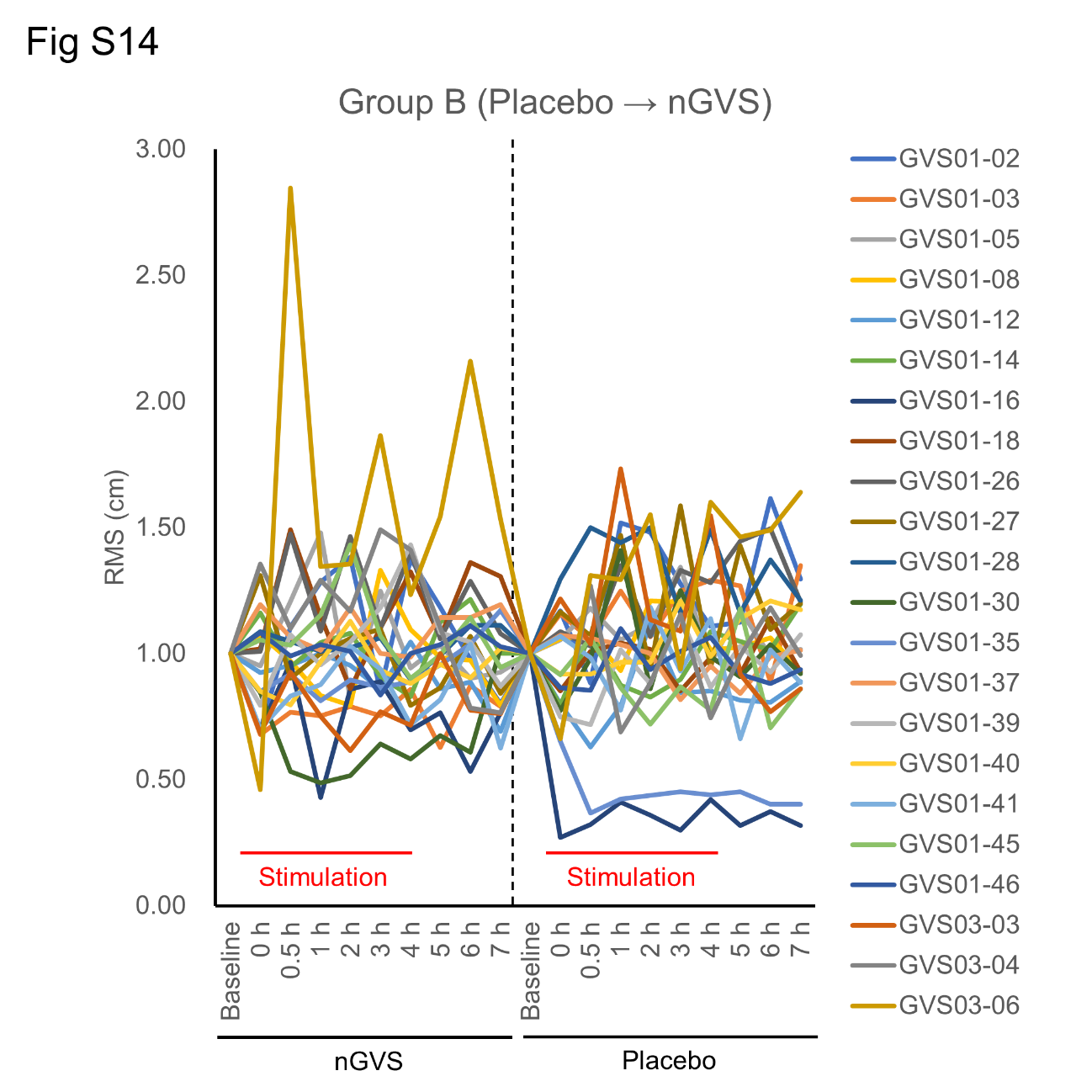


**Fig S14 Percent change from the baseline in RMS of COP movement for each enrolled subject in Group B.** nGVS = noisy galvanic vestibular stimulation, RMS = root mean square
