# Supplementary material for: Multicenter randomized double-blind placebo-controlled crossover study of the effect of prolonged noisy galvanic vestibular stimulation on posture or gait in vestibulopathy
Source: PLoS One. 2025 Jan 24;20(1):e0317822. doi: 10.1371/journal.pone.0317822 (PMC11760040; doi:10.1371/journal.pone.0317822)
Supplement: S4 File — (DOCX) [file pone.0317822.s004.docx]

# Study protocol

Randomized, double-blind, placebo-controlled, crossover study to confirm the efficacy and safety of percutaneous noisy galvanic vestibular stimulation to improve severe balance disorder in patients with refractory vestibulopathy

Clinical Trial Protocol

**Study protocol number: GVS001**

**Version: Ver 16.0 (Prepared on June 1, 2022)**

**Coordinating Investigator**

**Shinichi Iwasaki (Professor,** **Department of Otorhinolaryngology, Head and Neck Surgery, Nagoya City University Graduate School of Medical Sciences)**

# Clinical Trial Protocol Summary

Category: Investigator Initiated Trial

Name and symbol of investigational device: Portable vestibular current stimulator ・GVS-100

Development stage (phase): Verification study (phase III)

Prepared on: June 1, 2022

Version No. 16.0

Title:

A randomized, double-blind, placebo-controlled, crossover study to confirm the efficacy and safety of percutaneous noisy galvanic vestibular stimulation to improve severe balance disorder in patients with refractory vestibulopathy

Coordinating Investigator

Shinichi Iwasaki

Professor, Department of Otorhinolaryngology, Head and Neck Surgery, Nagoya City University Graduate School of Medical Sciences

1, Kawasumi, Mizuho-cho, Mizuho-ku, Nagoya, Aichi, Japan

Tel: 052-851-5511 (main)

Mail: iwashin-tky@umin.ac.jp

Clinical Trial Sites

This clinical trial will be conducted at the University of Tokyo Hospital (Department of Otolaryngology and Head and Neck Surgery), Tokyo Teishin Hospital (Department of Otolaryngology), and Nagoya City University Hospital (Department of Otorhinolaryngology, Head and Neck Surgery).

Clinical Trial Duration:

December 2018 to June 2022

(From the acquisition of consent from the first subject to the end of the clinical trial of the last subject)

Objective

The primary objective of this study is to compare the effects of transcutaneous noisy galvanic vestibular stimulation (noisy GVS [Galvanic Vestibular Stimulation]) using a portable vestibular current stimulator for 4 h on patients with severe unsteadiness symptoms based on vestibular disorder and to evaluate the effects of the stimulation on the improvement of body balance function during the stimulation. The secondary objective is to investigate the efficacy of noisy GVS after 4 h of stimulation and the safety of noisy GVS during and after stimulation.

Clinical Trial Design:

This is a multicenter, randomized, double-blind, placebo-controlled, crossover study.

As shown in Figure 1, the subjects whose written consent is obtained and whose eligibility is confirmed by screening are provisionally enrolled. Subsequently, only subjects for whom optimal intensity exist in the optimal intensity study will be enrolled in the main study. The subjects without optimal intensity will be discontinued at this point, and the study will be terminated after confirming safety 2 weeks later.

The subjects enrolled in the study will visit the hospital approximately two weeks after enrolment and will be randomly assigned in a 1:1 ratio to one of two groups: A group where the effect of optimal stimulation by noisy GVS on improving the body balance function is evaluated in ‘Session I’ followed by evaluation of the effect of placebo stimulation in ‘Session II’ (group A), and the group where the evaluation is performed in the reverse order with the effect of placebo stimulation on the body balance function being evaluated in ‘Session I’ followed by the evaluation of the optimal intensity of noisy GVS in ‘Session II’ (group B). A period of at least 7 days will be allowed between this enrolment and Session I.

Each subject will be stimulated with a portable vestibular current stimulator ("optimal stimulation for each subject" or "placebo stimulation") for 4 h to measure the unsteadiness and safety before and during the stimulation. In addition, the safety and efficacy of such stimulation will be evaluated for 3 h after stimulation to confirm all adverse events and incidents. After the evaluation of Session I, a period of approximately 14 days, but at least 7 days, shall be allowed before the evaluation of Session II. At 2 weeks after the end of the evaluation of "Session II" of the treatment stimulation period, the subjects will be interviewed for adverse events.

For safety, all the adverse events and incidents that occur during the use of the stimulator shall be confirmed.

**screening**

**Acquistion of constn**

**Confirmation of eligibili**

**Provisional enrollment**

**Consideration of optimal intensity**

2 weeks later

**Enrollment**

**Yes**

**Session I**

**Session II**

**Two weeks.**

**washout**

**4 h of GVS**

**4 h of placebo stimulation**

**3h later**

**3h later**

**4 h of placebo stimulation**

**3h later**

**4 h of GVS stimulation**

**3h later**

Group A

**Follow-up (tracing) survey**

**Confirmation of safety**

**End**

**No**

Group B

2 weeks later

**Double blind trial**

**Double blind trial**

6 weeks

2 weeks later

Figure 1. Flowchart of the clinical trial

Number of subjects:

A total of 60 patients (50 enrolled in this study, 40 completed) (Group A: 25 cases, Group B: 25 cases)

Study population:

Patients with severe unsteadiness associated with vestibular disorder who meet the following selection criteria and do not meet the exclusion criteria.

- Inclusion criteria:

1. Patients with unilateral or bilateral vestibular disorder on temperature stimulation test with 2 ml of ice water

- Unilateral impairment refers to CP (Canal Paresis) (%)=(healthy maximum slow phase velocity - affected maximal slow phase velocity)/(healthy maximal slow phase velocity + affected maximal slow phase velocity) x100 ≥ 20; bilateral impairment refers to peripheral vestibular disorder with no nystagmus induction or bilateral maximal slow phase velocity < 10 deg/s. In the calculation of CP, the duration of nystagmus may be used instead of maximum slow-phase velocity.

1. Patients with a total trajectory length of at least 180 cm in 60 s on a stabilometer while standing with closed eyes.
2. Patients who have experienced unsteadiness for at least 1 year after the onset of unsteadiness and whose symptoms have persisted for at least 6 months despite rehabilitation therapy.
3. Patients must be between 20 and 85 years of age and severely associated with vestibular disorders. The sex and status of hearing loss are irrelevant.
4. Patients who understand the details of this clinical trial and have given their written informed consent freely and voluntarily prior to participation in this clinical trial

- Exclusion criteria

1. Patients with metal in their body, such as cerebral artery clips, cochlear implants, or pacemakers (silver teeth are acceptable)
2. Patients with orthopedic diseases, such as fractures, sprains, separated flesh, etc., or patients with acute painful diseases, etc.
3. Patients with limb movement disorders due to cerebellar disorders or spinal cord disease
4. Patients with significant cardiac disease, including severe arrhythmias that may require a pacemaker (atrial fibrillation, severe QT prolongation syndrome, severe atrioventricular block (degree II or higher)), or severe heart failure that interferes with walking
5. Patients with malignant tumors
6. Patients with infectious diseases accompanied by fever, malaise, or dizziness
7. Pregnant and postpartum patients
8. Patients who cannot walk independently
9. Patients with skin abnormalities, such as infection or wound at the site of application, or patients with a history of anaphylactic shock or other severe allergies
10. Patients with lack of or limited legal capacity
11. Patients who have participated in another clinical trial (clinical study) within 3 months prior to the date of obtaining consent or who will participate in another clinical trial (clinical study) at the same time as this study
12. Other patients who have been judged to be inappropriate as subjects by the investigator (sub-investigator)

- Criteria for this registration

Patients with optimal intensity by noisy GVS

Clinical trial endpoints and assessments:

Primary endpoint:

The percentage change from baseline in the total trajectory length at all time points immediately after the start of noisy GVS by the investigational device after 30 min, 1 h, 2 h, and 3 h is measured using a Gravicorder G-620 (Anima, Inc.)

Secondary endpoints:

< Efficacy Evaluation>

- The percentage change from baseline in the outer circumference area and RMS (Root Mean Square [square root of the mean square of the distance between the mean position of all center-of-gravity sway and the measured center-of-gravity position]) values at all time points immediately after the start of noisy GVS, and at 30 min, 1 h, 2 h, and 3 h after the start of noisy GVS
- Percent change from the baseline in the total trajectory length, perimeter area, and RMS values at all time points of 4, 5, 6, and 7 h after the start of noisy GVS
- Ambulatory function

Percent change from baseline in the following items immediately after the start noisy GVS, and at 1 h, 2 h, 3 h, 4 h, 5 h, 6 h, and 7 h after the start of noisy GVS

- Dynamic Gait Index (pha) short form score
- Walking speed, stride length, step time, and left-right sway width during a 10-m walk measured using a gait analyzer (Walk-Mate Viewer, WALK-MATE LAB Co.)
- Subjective improvement score

The items below at immediately after the start noisy GVS, and at 1 h, 2 h, 3 h, 4 h, 5 h, 6 h, and 7 h after the start of noisy GVS

- - The subject's own evaluation in 5 steps of "1: improved, 2: slightly improved, 3: unchanged, 4: slightly worsened, and 5: worsened" compared to the state before the stimulation
- Quality of Life (QOL)

Change from baseline in the items below at 3 and 7 h after the start of noisy GVS

- - Japanese version of the modified Fall Efficacy Scale (mFES) (Appendix 2)
  - Japanese version of the Dizziness Handicap Inventory (DHI) (Appendix 3)
- Activity level

Activity (number of steps walked) immediately after the start of noisy GVS to 3 and 7 h after the start of noisy GVS

< Safety Evaluation>

Evaluate the occurrence of adverse events and defects.

# Table of contents

Page

1. Clinical Trial Protocol Summary 1

Table of contents 6

2. Definitions of abbreviations and terms 9

3. Introduction 10

3.1. Background and History 10

3.2. Non-clinical study summary 12

3.2.1. Mechanical and electrical testing 12

3.2.2. Biological Safety Assessment 13

3.3. Clinical Trial Summary 14

3.3.1. Results of Investigator-Initiated Clinical Trials 1 and 2 14

3.3.2. Safety 15

3.4. Results of an Investigator-initiated Clinical Study 38) 15

3.4.1. Carryover effects after the end of percutaneous noisy GVS 15

3.4.2. Status of variation in optimal intensity 17

3.4.3. Safety 17

3.5. Positioning of Clinical Trials 18

4. Objective 19

5. Clinical Trial Endpoints 19

5.1. Primary endpoint 19

5.2. Secondary endpoint 20

6. Clinical Protocol 21

6.1. Clinical Trial Design 21

6.1.1. Screening period 21

6.1.2. Examination of optimal intensity 21

6.1.3. Treatment Stimulation Period 21

6.1.4. Safety follow-up period 22

6.2. Discussion on the Clinical Trial Design 22

7. Subject Selection and Discontinuation Criteria 22

7.1. Subject inclusion criteria 22

7.1.1. Inclusion criteria 22

7.1.2. Exclusion criteria 23

7.1.3. Criteria for enrolment 24

7.2. Discontinuation of the Clinical Trial 24

7.2.1. Discontinuation of individual subjects 24

7.2.2. Discontinuation or interruption of the entire clinical trial 25

8. Investigational Device and Usage 25

8.1. Investigational device 25

8.1.1. Name of investigational device.. 25

8.1.2. Purpose of use in the clinical trial 25

8.1.3. Configuration of the investigational device 25

8.1.4. Display 26

8.1.5. Handling and storage of investigational devices 26

8.1.6. Precautions for use 27

8.1.7. Risk Management 27

8.2. Management of investigational devices 28

8.3. Use compliance 28

8.4. Use of drugs and medical devices other than the investigational device 28

9. Subject Consent 29

9.1. Consent Form 29

9.2. When important information affecting the subject's decision is obtained (Revision of the Written Explanation and Consent Form ) 29

10. Subject Enrollment 30

10.1. Preparation of a subject screening name list 30

10.2. Enrollment Procedure 30

10.3. Method of randomization and maintenance of blindness 30

10.3.1. Key opening procedure 31

10.3.2. Key opening procedures during clinical trials 31

11. Observation, examination, and investigation items of the clinical trial, and duration of the trial 33

11.1. Clinical Trial Schedule 33

11.2. Subject Background and Screening Tests 34

11.3. Examination of optimal intensity 34

11.3.1. Special note on stimulation 35

11.3.2. Stimulus type 35

11.3.3. Stimulation method 36

11.3.4. Therapeutic stimulus 37

11.3.5. Treatment Stimulation Method 38

11.3.6. Endpoints 38

11.4. Restricted concomitant medications and prohibited concomitant therapies 39

11.5. Concomitant medications/ therapy 39

11.6. Safety evaluation 39

11.6.1. Definition of adverse events and defects 40

11.6.2. Duration, frequency, and methods to examine adverse events/defects and serious adverse events/defects 40

11.6.3. Definition of serious adverse events 41

11.6.4. Response to serious adverse events and defects 41

11.6.5. Expected adverse effects. 43

12. Data Analysis and Statistical Considerations 43

12.1. Study design considerations 44

12.1.1. Setting of the number of cases 44

12.1.2. Rationale for setting the number of cases 44

12.1.3. Re-estimation of the number of subjects 47

12.2. Consideration of data analysis 47

12.2.1. Completed cases 47

12.2.2. Analysis sets 47

12.2.3. Interim analysis 47

12.2.4. Efficacy Analysis Methods 47

12.2.5. Safety analysis Methods 51

13. Clinical Trial Quality Management and Quality Assurance 52

13.1. Regulatory and ethical considerations, including procedures for obtaining consent 52

13.2. Notification of the Clinical Trial Protocol to the Regulatory Authorities 52

13.3. Deviations and amendment to the Clinical Trial Protocol 52

13.3.1. Deviation from the Clinical Trial Protocol 52

13.3.2. Amendment to the Clinical Trial Protocol 53

13.4. Quality control 53

13.4.1. Monitoring 53

13.4.2. Data management 54

13.5. Quality assurance 55

13.6. Discontinuation of the clinical trial at the medical institution 55

13.7. Record Keeping 56

13.8. Provision of clinical trial results and information to investigators 57

13.9. Clinical Trial Period 57

14. Clinical Trial Implementation System 57

15. References 57

16. Appendix 1. Dynamic Gait Index 59

17. Appendix 2. Modified Fall Efficacy Scale 68

18. Appendix 3. Dizziness Handicap Inventory 69

# Definitions of abbreviations and terms

| BMI | Body-Mass Index |
| --- | --- |
| GCP | Good Clinical Practice (Standards for the Conduct of Clinical Trials of Medical Devices) |
| GVS | Galvanic Vestibular Stimulation |
| LED | Light Emitting Diode |
| RMS | Root Mean Square (square root of the mean of the squares of the distances between the mean position of the total center of gravity sway and the measured center-of-gravity position) |
| DGI | Dynamic Gait Index |
| DHI | Dizziness Handicap Inventory |
| FAS | Full Analysis Set (largest analysis population) |
| mFES | modified Fall Efficacy Scale |
| PPS | Per Protocol Set (analysis population that conforms to the study protocol) |
| SAS | Safety Analysis Set |

#

# Introduction

## Background and History

1) Epidemiology, pathogenesis, and prognosis of subject diseases

The annual number of deaths due to falls among the elderly in Japan has been increasing every year; additionally, in 2009, the number was approximately 7,300, exceeding the number of deaths due to traffic accidents. Falls and fractures are the main causes of death and becoming bedridden among the elderly, and the prevention of falls has become one of the most important issues for the Health, Labour, and Welfare Administration. In particular, bedridden patients due to fractures are one of the causes of dementia, and the development of effective measures to prevent falls among the elderly is an urgent issue for Japan, which is facing a rapidly aging society.

One of the most common causes of falls in the elderly is dizziness and balance disorder, approximately 40% of which are attributed to peripheral vestibular disorders. It has been reported that the risk of falling in the elderly with dizziness and balance disorders is approximately three times that of the elderly without such disorders. The treatment of vestibular disorders is extremely important in terms of the prevention of falls and fractures in the elderly.

Vestibular disorders are caused by damage to the vestibule in the inner ear, which controls the sense of balance, and the main symptoms are marked dizziness, unsteadiness, and difficulty in walking. Although the disease per se does not have a significant impact on the prognosis of life, it affects the activities of daily living of the patient and significantly impairs the quality of life. Furthermore, as mentioned above, it can increase the risk of falls, especially in the elderly.

The exact number of patients with vestibular disorders is unknown; however, estimates from telephone interviews in Germany report that approximately 8% of adults experience dizziness due to vestibular disorders^1)^. In addition, screening for vestibular disorders in the United States reported that 35% of adults have some form of vestibular disorder, with or without symptoms of dizziness, and a 12-fold increased risk of falling^2)^.

(2) Current treatment methods of the subject diseases and their problems

For dizziness and balance disorders caused by a unilateral peripheral vestibular disorder, it is effective to promote vestibular compensation by rehabilitation. In unilateral vestibular disorders, the phenomenon of vestibular compensation, in which the symptoms of dizziness and balance disorder gradually lessen over time without recovery of vestibular function is known, and is thought to be achieved based on the plasticity of the central nervous system, mainly in the cerebellum and brainstem. In vestibular rehabilitation, the vestibular compensation is promoted by actively moving the body and providing effective stimulation to the vestibular, oculomotor, and deep sensory systems involved in body balance, thereby improving the symptoms of dizziness and dynamic state of equilibrium disorders. Although this vestibular rehabilitation can improve balance disorders to some extent, it does not necessarily eliminate dizziness and balance disorders completely. There are also a small number of cases of unilateral vestibular disorders in which vestibular rehabilitation is not effective and dizziness and balance disorder remain permanently.

In addition, vestibular rehabilitation is not effective for bilateral peripheral vestibular disorders, and there is no effective treatment for these disorders. Although vestibular regeneration has been investigated as a potential fundamental treatment, it is still in the research stage and has not yet been applied clinically. Moreover, as a treatment for refractory bilateral vestibular disorders, an artificial vestibule that replaces the vestibular function by inserting stimulating electrodes into the three semicircular canals and directly stimulating the nerves electrically in the semicircular canals is being developed in Europe and the United States. However, no clinical trials have been reported and evidence as a treatment method has not been established. In addition, this treatment is also surgically invasive, and the implantation of electrodes into the semi-circular canal may cause hearing loss and worsen the vestibular disorder. Furthermore, the cost of this treatment is high. Therefore, the development of a safer and simpler treatment method is strongly desired.

(3) Principles and benefits of the treatment method

Galvanic vestibular stimulation (GVS) is a method of electrical current stimulation of the vestibular nerve and is conventionally used in actual clinical practice to test the vestibular function. Noisy GVS is a method in which the current used for vestibular stimulation is a weak noisy current. Recently, the stochastic resonance phenomenon, in which a nonlinear response to a weak input signal is reinforced by noise^3）^(Fig. 2), has attracted attention, and its principle has been elucidated^4）^, suggesting that noisy GVS is related to this stochastic resonance phenomenon. Furthermore, it has been shown that noisy GVS, which is weak enough not to cause side effects, such as pain or discomfort, is effective in improving the autonomic reflexes and performance in neurodegenerative diseases, such as Parkinson's disease. Portable noisy GVS has various advantages, such as no surgical invasion, low cost; additionally, the patient does not feel stimulation during use because the balance disorder is treated by applying a surface electrode to the back of the patient's ear and applying a weak current using a small stimulator. If noisy GVS can be shown to be effective in treating refractory peripheral vestibular disorders, it will be possible to treat a large number of patients suffering from dizziness and balance disorders using a simple, non-surgical method.

Threshold

Fig. 2. Principle of stochastic resonance

A nonlinearity signal response is reinforced by providing noise of appropriate magnitude.

(Left) Signals below the threshold bring no information. (Center) When noise of an appropriate magnitude is provided, signals above the threshold appear and the information is increased. (Right) When a larger signal is provided, the information decreases.

## Non-clinical study summary

### Mechanical and electrical testing

#### Electrical safety

The electrical safety of the investigational device was conducted by the Japan Quality Assurance Organization in accordance with the requirements of JIS T 0601-1:2017 "Medical electrical equipment Part 1: General requirements for basic safety and basic performance", and it was determined that all the items met the standard and that the electrical safety of the product was sufficiently ensured.

#### Mechanical safety

The mechanical safety of the investigational device was conducted by the Japan Quality Assurance Organization in accordance with the requirements of JIS T 0601-1:2017 "Medical electrical equipment Part 1: General requirements for basic safety and basic performance", and it was determined that all items met the standard and that the mechanical safety of the product was sufficiently ensured.

#### Electromagnetic compatibility

The electromagnetic compatibility of the investigational device was verified by the Japan Quality Assurance Organization (JQA) in accordance with the requirements of JIS T 0601-1-2:2012 "Part 1-2: General requirements for safety of electrical equipment for medical use - Electromagnetic compatibility - Requirements and tests, and it was determined that all the items met the standard and that electromagnetic compatibility of the product was sufficiently ensured".

#### Specific requirements for basic safety and basic performance of nerve and muscle stimulators

The basic safety and basic performance of the nerve and muscle stimulators for this experimental device was conducted by the Japan Quality Assurance Organization in accordance with the requirements of JIS T 0601-2-10:2015 "Medical electrical equipment Part 2-10: Individual requirements for basic safety and basic performance of nerve and muscle stimulators". It was determined that all the items met the standard and that the individual requirements were judged to be sufficiently secured.

### Biological Safety Assessment

In this experimental device, the device in contact with the human body is an electrode.

Since this electrode is an approved product, no new biological safety evaluation was conducted at this time.

## Clinical Trial Summary

### Results of Investigator-Initiated Clinical Trials 1 and 2

#### Effects of Short-Term Stimulation

The effects of noisy GVS on body balance in the standing position were examined in 21 normal adults and 11 patients with bilateral vestibular disorders. Noisy GVS improved the body balance in 76% of normal subjects and 91% of patients with bilateral vestibular disorders when compared with no stimulation at 80% of the sensory threshold. The improvement in body balance by noisy GVS was greater in patients with bilateral vestibular impairment than in normal subjects5^）^(Figure 3).

A. Electrodes were affixed to the posterior part of the ear and the patient stood on the gravimeter for 30 s with closed eyes while noisy GVS was applied.

B. Effect of noisy GVS in a case of bilateral vestibular disorder (mitochondrial encephalomyopathy). Notably, 400 μA of noisy GVS markedly improved the balance disorder.

**Fig. 3. Effect of noisy GVS on the improvement of balance in patients with bilateral vestibular disorders**

#### Sustained stimulation effect

We conducted an investigator-initiated clinical trial to investigate the effects of sustained stimulation in healthy elderly subjects in 2014.

The results showed that the noisy GVS for 30 min had a lasting effect for 3 h after the end of stimulation^6)^.

### Safety

#### Safety of Investigator-Initiated Clinical Trial 1

Noisy GVS was performed for 30 s using the GVS-100 in normal subjects and patients with bilateral vestibular disorders, and no adverse events were observed during or after stimulation5^)^.

#### Safety of an Investigator-initiated Clinical Study 2

In an investigator-initiated clinical study in healthy subjects, noisy GVS long-term stimulation (30-min and 3-h stimulation) was performed using GVS-100, and no adverse events were observed during or after stimulation^7)^.

## Results of an Investigator-initiated Clinical Study 3^8)^

From October 2016 to January 2017, we conducted an exploratory clinical trial in 13 patients with bilateral vestibular disorders at our hospital to examine the safety of long-term 30-min percutaneous noisy GVS, the carryover effect after the end of stimulation, and whether the optimal stimulation intensity varied twice with an interval of 2 weeks. In Session I, only the optimal intensity was examined, and in Session II and III, the safety and carryover effects of long-term noisy GVS were examined.

### Carryover effects after the end of percutaneous noisy GVS

In patients with bilateral peripheral vestibular disorders, the total trajectory length was significantly improved from baseline at 3 h after stimulation with 30 min of noisy GVS (total trajectory length change in Session II: Fig. 4). A test of contrast comparing the mean values after noisy GVS with baseline values in a mixed-effects model showed that, on average, there was a significant improvement in the total trajectory length up to 6 h after the end of stimulation in both Session II and III (Table 1). In addition, the subjective degree of improvement measured on a 5-point scale also showed a trend toward improvement (Fig. 5). The degree of subjective improvement was correlated with the total trajectory length, suggesting the clinical significance of the total trajectory length.

**Table 1. Analysis of average change from baseline**

| Parameter |  | *P*-value | |
| --- | --- | --- | --- |
|  |  | Up to three h | Up to six h |
| Total trajectory length | Session II | <.001 | 0.004 |
|  | Session III | <.001 | 0.002 |
| Subjective symptom score | Session II | 0.064 | 0.033 |
|  | Session III | 0.023 | 0.020 |

Least squares mean of changes over time in the amount of change in

subjective symptom score in Phase II


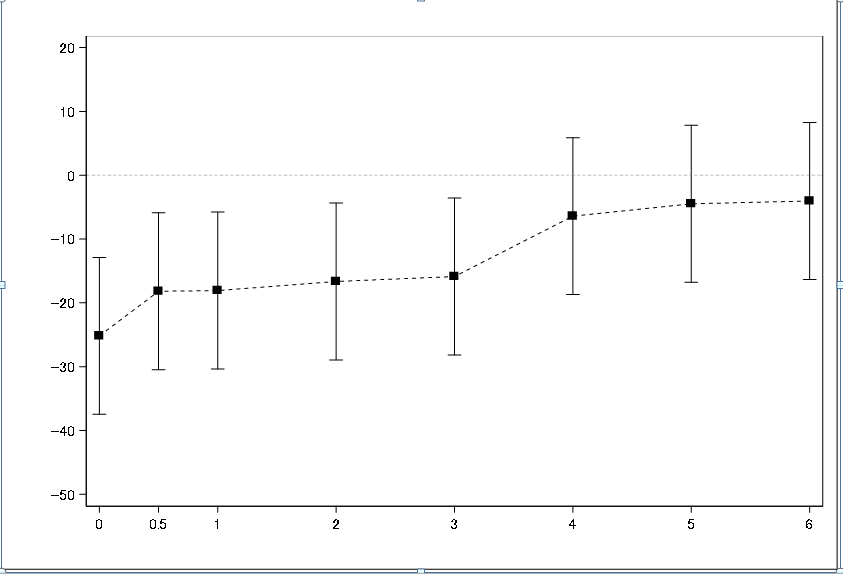


Least squares mean ± 95% confidence interval

Time after stimulation (h)


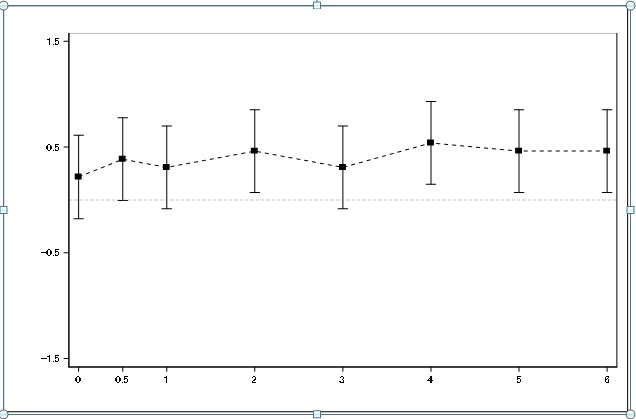


Least squares mean ± 95% confidence interval

Least squares mean of changes over time in the amount of change in

subjective symptom score in Phase II

**Fig. 4. Change in the total trajectory length over time (with eyes closed)**

**Fig. 5. Change in the subjective symptom score over time (with eyes closed)**

Time after stimulation (h)

### Status of variation in optimal intensity

The optimal intensity was defined as the current intensity that was below the sensory threshold, had improvement in all the three parameters measured by the stabilometer, and showed the most improvement in the total trajectory length. The optimal intensity was present in all cases before stimulation with noisy GVS in the second and third periods. There were 8 cases in which the optimal intensity could be measured at all time points, and 5 cases in which the optimal intensity could not be measured at any time point.

The optimal intensity in each case ranged from 100 µA to 1000 µA. Few cases remained constant at each measurement time point, indicating a diurnal variation in the optimal intensity. A decreasing trend in the optimal intensity was also observed over the course of the day.

### Safety

A list of all the reported adverse events is presented in Table 2.

There were no reports of serious adverse events or failures leading to serious adverse events.

Three adverse events were observed in 2 of 13 patients. The adverse events were cerebral infarction, dizziness, and right-sided hearing loss in one case each. Of these, there was no event (adverse drug reaction) for which a causal relationship to noisy GVS could not be ruled out.

The severity of the adverse events was moderate (cerebral infarction) or mild (dizziness, right-sided hearing loss), but not severe. None of the events were serious and resolved without discontinuation or treatment.

There were no incidents that were not associated with adverse events.

Table 2. List of adverse events

Statistical analysis report: see Listing 6.1_1

| Sex/  age  (Age in years) | Height  (cm)/  weight  (kg) | Onset date/  comment | Name of adverse event name | Severity | Causal relationship with noisy GVS | Severity | Status of discontinuation | Status of Treatment | Date of outcome/.  outcome |
| --- | --- | --- | --- | --- | --- | --- | --- | --- | --- |
| Male  78 | 154/  47 | 2016-11-11/  Occurred the next day of starting Session III (after the end of the study period) | Cerebral infarction | Moderate | No causal relationship | Not serious | None | None | 2016-11-27/  recovery |
| Male  45 | 165/  58 | 2016-11-08/  Occurred on the day of starting Session II ( before the start) | Dizziness | Mild | No causal relationship | Not serious | None | None | 2016-11-08/  recovery |
| Male  45 | 165/  58 | 2016-10-28/  Occurred on the day of starting Session II (before the start) | Right-sided deafness | Mild | No causal relationship | Not serious | None | None | 2016-11-08/  recovery |

## Positioning of Clinical Trials

In this clinical trial, we will expand the subjects from patients with bilateral vestibular disorders to patients with vestibular disorders, including unilateral vestibular disorders with severe body balance disorder, and investigated the effect of noisy GVS on improving the body balance function during stimulation as the main objective, as well as the carryover effect after the end of stimulation and safety as a secondary objective. The secondary objective of this study is to develop a treatment device that may reduce the risk of falling and improve the quality of life of patients with severe unsteadiness associated with vestibular disorder.

# Objective

Primary objective:

The primary objective of this study is to compare the effects of transcutaneous noisy galvanic vestibular stimulation (noisy GVS [Galvanic Vestibular Stimulation]) using a portable vestibular current stimulator for 4 h on patients with severe unsteadiness symptoms based on vestibular disorder, and to evaluate the effects of the stimulation on the improvement of body balance function during the stimulation.

Secondary objective

The secondary objective of this study is to investigate the efficacy of noisy GVS for 4 h, including improvement in gait function and subjective symptoms during stimulation and up to 3 h after the end of the 4-h stimulation, as well as the safety during and after the stimulation.

# Clinical Trial Endpoints

## Primary endpoint

The percentage change from baseline in the total trajectory length at all time points immediately after the start, after 30 min, 1 h, 2 h, and 3 h of noisy GVS by the investigational device measured using a Gravicorder G-620 (Anima, Inc.)

[Primary endpoint]

The difference between the mean rate of change in the total trajectory length from immediately after the start of stimulation to 3 h after stimulation in the GVS period and the mean rate of change in the total trajectory length from immediately after the start of stimulation to 3 h after stimulation in the placebo period is compared.

[Rational for the setting]

The correlation coefficient between the mean change in the total trajectory length up to 3 h measured in the investigator-initiated clinical trial and the total subjective improvement score (possible range [-10, 10], with higher scores indicating subjective improvement) was -0.60 (*P* = 0.032), suggesting the clinical utility of the total trajectory length. Because total trajectory length was measured at 5 time points (immediately after the start, after 30 min, 1 h, 2 h, and 3 h of noisy GVS) during the 4-h noisy GVS in this trial, the percentage change from baseline in total trajectory length at 5 time points was set as the primary endpoint.

## Secondary endpoints

1. Secondary efficacy endpoints were as follows:
2. The percentage change from baseline in the outer circumference area and RMS (Root Mean Square [square root of the mean square of the distance between the mean position of all center-of-gravity sway and the measured center-of-gravity position]) values at all time points immediately after the start, after 30 min, 1 h, 2 h, and 3 h of noisy GVS
3. Percent change from baseline in the total trajectory length, perimeter area, and RMS values at all the time points 4, 5, 6, and 7 h after the start of noisy GVS
4. Ambulatory function

Percent change from baseline in the following items immediately after the start, after 1 h, 2 h, 3 h, 4 h, 5 h, 6 h, and 7 h of noisy GVS.

- - Dynamic Gait Index (DGI) short form scores^9)^ (Appendix 1)
  - Walking speed, stride length, step time, and left-right sway width during a 10-m walk measured using a gait analyzer (Walk-Mate Viewer, WALK-MATE LAB Co.

1. Subjective improvement score

- The following items immediately after the start after 1 h, 2 h, 3 h, 4 h, 5 h, 6 h, and 7 h of noisy GVS. The subject's own evaluation in 5 steps of "1: Improved, 2: Slightly improved, 3: Unchanged, 4: Slightly worsened, 5: Worsened" compared to the state before stimulation.

1. Quality of Life (QOL)

Change from baseline in the following items at 3 and 7 h after the start of noisy GVS

- Japanese version of the modified Fall Efficacy Scale (mFES) (Appendix 2)
- Japanese version of the Dizziness Handicap Inventory (DHI) (Appendix 3)

1. Activity level

- Activity (number of steps walked) from immediately after the start of noisy GVS to after 3 h and 7 h of noisy GVS.

[Rationale for the setting]

1. Because the outer circumference area and RMS are important center-of-gravital sway parameters other than the total trajectory length.
2. To compare the data before the start of noisy GVS and immediately after the end of long-term stimulation, and to examine the carry-over effect on center-of-gravity sway after the end of stimulation.
3. The DGI is a validated gait function test, to objectively measure the gait function along with the gait speed, stride length, and step time.
4. The degree of improvement as perceived by the subjects themselves is measured using a simple 5-point scale.
5. Fear of falling and dizziness will be measured using a validated quality of life questionnaire.
6. To check the correlation between the amount of activity and other assessment items.
7. The safety endpoints are as follows:

Evaluate the occurrence of adverse events and defects.

# Clinical Protocol

## Clinical Trial Design

This is a multicenter, double-blind, randomized, placebo-controlled crossover study in patients with severe unsteadiness associated with vestibular disorder.

### Screening period

The investigator will confirm the eligibility of subjects from whom written informed consent has been obtained and will "provisionally enrol" the subjects who meet the inclusion criteria (meet the selection criteria and do not meet the exclusion criteria).

### Examination of optimal intensity

The optimal intensity with noisy GVS is determined for each provisionally enrolled subject. The subjects for whom the optimum stimulus has been determined are "fully enrolled". On the other hand, the subjects for whom the optimal intensity does not exist are discontinued at this point, and the study will be terminated after confirming safety after 2 weeks.

### Treatment Stimulation Period

Subjects enrolled in the study will visit the hospital approximately 2 weeks after enrolment and will be randomly assigned in a 1:1 ratio to one of two groups: A group where the effect of optimal stimulation by noisy GVS on improving body balance function is evaluated in ‘Session I’ followed by evaluation of the effect of placebo stimulation in ‘Session II’ (group A), and the group where the evaluation is performed in the reverse order with the effect of placebo stimulation on the body balance function being evaluated in ‘Session I’ followed by the evaluation of the optimal intensity of noisy GVS in ‘Session II’ (group B). A period of at least 7 days will be allowed between this enrolment and Session I.

Each subject will be stimulated with a portable vestibular current stimulator ("optimal stimulation for each subject" or "placebo stimulation") for 4 h, and the unsteadiness and safety before and during stimulation will be measured. In addition, the safety and efficacy will be evaluated for 3 h after stimulation, and all the adverse events and defects will be confirmed. After the Session I evaluation, a period of 14 days, but at least 7 days, will be allowed between the Session I and Session II evaluations.

For safety, all the adverse events and malfunctions that occur during the use of the stimulator shall be confirmed.

### Safety follow-up period

Two weeks after the end of the evaluation of "Session II" of the treatment stimulation period, the patients will be interviewed regarding the presence of adverse events.

## Discussion on the Clinical Trial Design

Because of the large inter-patient variability in the GVS sway test used as the primary endpoint, data from both the percutaneous noisy GVS and placebo stimulation periods in the same patient can be collected to reduce the inter-patient variability and improve the accuracy. Because the number of patients with vestibular disorders who have severe wobbling that would qualify for this study is likely to be very limited, although no definitive data are available, and because the procedures for assessing gravity sway and gait function are somewhat complex, this study was designed as a crossover study to ensure the maximum accuracy of results in a limited number of centers and patients.

Regarding the washout period, it was shown in the investigator-initiated clinical trial 3 (section 4.4) that if noisy GVS was performed after a 2-week interval, there would be no residual effect on subsequent gravimetric sways. Although a 14-day washout period was used as a baseline, a minimum washout period of 7 days is considered as acceptable in order to minimize the deviations from the protocol due to patient convenience, and because the results of an examination of the carryover effect after the end of percutaneous noisy GVS showed no carryover effect after 4 h post-stimulation. Therefore, a minimum washout period of 7 days was set as acceptable.

The stimulus with a noisy GVS current of 0 μA is the placebo stimulus, and the current with the intensity that showed the most improvement below the sensory threshold is the optimal intensity.

# Subject Selection and Discontinuation Criteria

## Subject inclusion criteria

Patients with severe unsteadiness based on intractable vestibular disorder who meet the following selection criteria and do not meet the exclusion criteria.

### Inclusion criteria

1. Patients with unilateral or bilateral vestibular disorder on a temperature stimulation test with 2 mL of ice water

- The unilateral impairment is CP (Canal Paresis) (%)=(maximal slow phase velocity on the healthy side - affected maximal slow phase velocity on the affected site)/(maximal slow phase velocity on the healthy side + maximal slow phase velocity on the affected side) x100 ≥ 20, A bilateral impairment is peripheral vestibular impairment with no nystagmus induction or bilateral maximal slow phase velocity < 10 deg/s. In the calculation of CP, the duration of nystagmus may be used instead of the maximum slow-phase velocity.

1. Patients with a total trajectory length of at least 180 cm in 60 s on a stabilometer when standing with eyes closed.
2. Patients who have been wobbly for at least 1 year after the onset of unsteadiness and whose unsteadiness symptoms have persisted for at least 6 months despite rehabilitation therapy.
3. Patients must be between 20 and 85 years of age and severely associated with vestibular disorders. The sex and status of hearing loss are irrelevant.
4. Patients who understand the details of this clinical trial and have given their free and voluntary written consent prior to participation in this clinical trial.

Rationale for setting the inclusion criteria

1. The definition of vestibular dysfunction is a conventionally used criterion and internationally accepted (Bilateral vestibular disorders^5）^, Unilateral vestibular disorders^6）^).
2. The criterion for severe unsteadiness was the mean +2 SD of healthy subjects aged 65 years and older (data provided by Anima).
3. We set this as a criterion for intractable unsteadiness.

4) ～5) The upper age limit was set in consideration of safety and to obtain appropriate informed consent.

### Exclusion criteria

If any of the following conditions are met, the product will not be eligible for inclusion in a clinical trial.

1. Patients with metal in the body, such as cerebral artery clips, cochlear implants, or pacemakers (silver teeth are acceptable)
2. Patients with orthopedic diseases (e.g., fractures, sprains, separated flesh, acute painful diseases, etc.)
3. Patients with limb movement disorders due to cerebellar disorders or spinal cord disease
4. Patients with significant cardiac disease, including severe arrhythmias that may require a pacemaker (atrial fibrillation, severe QT prolongation syndrome, severe atrioventricular block (degree II or higher)), or severe heart failure that interferes with walking
5. Patients with malignant tumors
6. Patients with infectious diseases accompanied by fever, malaise, or wobbliness
7. Pregnant and postpartum patients
8. Patients who cannot walk independently
9. Patients with skin abnormalities (infection, wound, etc.) at the site of application, or patients with a history of anaphylactic shock or other severe allergies
10. Patients with lack of or limited legal capacity
11. Patients who have participated in another clinical trial (clinical study) within 3 months prior to the date of obtaining consent, or who will participate in another clinical trial (clinical study) at the same time as the clinical trial
12. Other patients who are judged to be inappropriate as subjects by the investigator (or subinvestigator)

[Rationale for setting the exclusion criteria]

### Criteria for this enrolment

Patients with optimal intensity by noisy GVS

Rational for setting these enrolment criteria

The data were set up for the evaluation of efficacy.

## Discontinuation of the Clinical Trial

### Discontinuation of individual subjects

Discontinuation criteria:

During the clinical trial period, the investigator will discontinue the clinical trial for a subject if the subject falls under any of the following conditions

1. If it is found after the start of the clinical trial that the selection criteria are not met or that the inclusion/exclusion criteria are violated
2. If the investigator determines that continuation of the study is difficult due to the occurrence of an adverse event
3. The subject wishes to discontinue the clinical trial or withdraws consent to participate in the clinical trial (dropout)
4. The subject has significantly failed to comply with the stipulations of the study protocol
5. Subject did not continuously return to the study site (untraceable)
6. When the investigator conducting the clinical trial personally discontinues the clinical trial, or when the Institutional Review Board ("IRB") or MHLW instructs the discontinuation of the clinical trial
7. Other cases in which the investigator (subinvestigator) determines that continuation of the study will be difficult or disadvantageous to a subject

### Discontinuation or interruption of the entire clinical trial

The investigator who conducts the clinical trial shall decide whether or not to continue the clinical trial if any of the following items apply

1. Significant information regarding the quality, safety, or efficacy of the investigational device is obtained.
2. When it is judged to be extremely difficult to achieve the planned number of patients due to the difficulties in recruiting subjects.
3. When the IRB has instructed an amendment to the protocol, and it is judged to be difficult to accept the amendment.

If the IRB recommends or directs discontinuation, the clinical trial will be terminated.

When a decision is made to discontinue or suspend a clinical trial, the decision shall be promptly reported in writing to the hospital director (or the director of each medical institution) together with the reasons for the discontinuation or suspension.

# Investigational Device and Usage

## Investigational device

### Name of investigational device

1. Name of the investigational device

- Portable Vestibular Current Stimulator (GVS-100)

1. Regulatory Classification of the device under test

- Category : Instruments and apparatus 12: Physical therapy instrument and apparatus
- Generic Name: Low-frequency therapy device
- Classification: Class II

### Purpose of use in the clinical trial

Improvement of severe unsteadiness based on vestibular disorder

### Configuration of the investigational device

This device, "Portable Vestibular Current Stimulator," consists of (1) the main body of the stimulator and (2) electrode cables. Electrode*A (Blue Sensor N-00-S/25, manufacturer and distributor: Metts Corporation), electrode*B (Blue Sensor NF-00-S/12, manufacturer and distributor: Metts Corporation), or electrode*C (disposable electrode F Vitrode, manufactured and distributed by Nihon Kohden Corporation) is applied to the skin behind the ear and connected to the main body of the stimulator through the electrode cable to administer electrical stimulation.

*: Electrodes should be disposable and commercially available.

### Display

　A label is attached to the stimulator body of the investigational device and the following information is described in Japanese.

The fact that the product is for investigational use.

(Name and title of the Coordinating Investigator and address)

Name or identification code of the raw material

Serial number or serial code

Storage Method

### Handling and storage of investigational devices

The investigational devices are to be stored in a secure location under appropriate physical conditions. Only the investigator (or subinvestigator) and authorized site personnel shall handle and manage the investigational device(s). The investigational device is to be used only for subjects who have been enrolled into the clinical trial in accordance with the study protocol. (2) The investigational devices shall be handled and managed appropriately in accordance with the "Investigational Device Summary Sheet" and "Standard Operating Procedures for Investigational Device Management" provided by the coordinating investigator.

#### Method of use

Preparation

1. Clean and dry the skin behind the subject's ears.
2. Affix the electrodes to the left and right mastoid process areas.
3. Connect the electrode cable and reinforce it with adhesive plaster.
4. Record the type of electrode used.

In use

1. Turn on the power switch.
2. Power ON/BATT LED lights up in 2-s cycles (normal operation).
3. Discontinue use when the power ON/BATT LED is not operating normally.
4. After the specified stimulation time has elapsed, turn off the power, remove the electrodes, and terminate use.
5. When electrode disconnection occurs, the LED flashes quickly with a 0.4-s cycle. At this time, the current output stops. To recover, turn off the power once, reaffix the electrodes properly, and turn the power back on for at least 6 s after turning off the power. If the LED blinks slowly with a 2-s cycle when the power is turned on, the device is operating normally. In addition, it has been confirmed that flashing of the LED remains lit or does not light up, although this is a very rare anomaly. In this case, follow the recovery method described above.

### Precautions for use

**Precautions when using the equipment**

- Monitor the equipment in general and patients for abnormalities.
- If any abnormality is detected in the equipment or a patient, ensure patient safety and discontinue use of the equipment.
- The electrodes are disposable products and should not be reused.

The average battery life at waveform B, range 2 mA, and output adjustment 99% is approximately 16 h. The batteries are used for 4 h of use per time, and the battery should be replaced after 4 uses. When the battery voltage drops, the LED blinks in 0.8-s cycles, and the battery should be replaced.

**Other precautions**

- When re-using equipment that has not been used for a long period of time, always make sure that the equipment is in good and safe working order before use. Failure to do so may cause electric shock or malfunction.
- When a malfunction occurs, do not operate the unit without permission, provide appropriate indications, and contact a repair company.
- Do not modify the equipment without permission, and do not connect it to equipment made by other companies.

#### Storage and expiration date

**Storage and keeping method**

- Install the equipment in a location where it will not be exposed to water or other liquids.
- Install the equipment in a location where the air pressure, temperature, humidity, ventilation, sunlight, dust, salt, sulfur-containing air will not adversely affect the equipment.

**Expiration date**

The period shall be one year from the date when the shipment acceptability is determined. Thereafter, periodic inspections shall be conducted every year, and the period shall be one year from the date of periodic inspections.

### Risk Management

#### Overview of risk analysis, including the identification of residual risks

Based on the risk analysis conducted by the manufacturer, the following risks are considered for this investigational device: (1) loss of performance due to output shutdown, (2) circuit function shutdown due to reverse battery connection, and (3) burn hazard due to battery heat generation caused by a short circuit in the power supply line.

As measures to reduce these risks, the residual risk was reduced by maintaining the standard through periodic maintenance for (1), adding series diodes and polarity labels for (2), and adding chip fuses for (3).

#### Risk Assessment Results

As a result of conducting risk management of this investigational device in accordance with JIS T 14971: 2012 "Medical devices - Application of risk management to medical devices" using the risk analysis procedures predefined by the manufacturer, no residual risk was deemed as unacceptable by reducing the estimated risk to an appropriate level for each hazard identified.

## Management of investigational devices

The investigator (or subinvestigator), designated site staff, or (if applicable) head of the medical institution should record the quantities of investigational devices used on subjects, those received from the investigator conducting the study, and those returned to the investigator conducting the study, as appropriate. The management records of investigational devices shall be kept throughout the clinical trial period.

The investigational device manager at the medical institution shall properly handle all investigational devices in accordance with the "Standard Operating Procedures for Investigational Device Management" submitted by the medical institution's own investigator.

## Use compliance

Use of the investigational device will be conducted under the supervision of the investigator (or subinvestigator). The information related to the use of each subject will be recorded in the Case Report Form.

## Use of drugs and medical devices other than the investigational device

If the investigator determines that it is necessary to use a drug or medical device other than the investigational device, the investigator should record the name of the drug or medical device (product name if possible), dosage, route of administration, date of administration, and purpose of use in the Case Report Form. However, new use of anxiolytics, antidepressants, sleeping pills, dizziness drugs, and drugs indicated for dizziness in the indications is prohibited (see Section 11.4).

# Subject Consent

　The consent of the subject shall be obtained following the content and procedures outlined below, in accordance with all the applicable regulatory requirements prior to the subject’s participation in the clinical study.

## Consent Form

　Prior to the start of the screening test for this clinical trial, the investigator (or subinvestigator) shall fully explain the explanatory items for subjects stipulated in the GCP using the Written Explanation and Consent Form approved by the IRB to those who are considered appropriate as subjects. At that time, the subjects shall be given the opportunity to ask questions and provided with sufficient time to decide whether or not to participate in the clinical trial. Subsequently, after the subject's free and voluntary consent is obtained, the subject's signature or name and seal shall be obtained in the Written Explanation and Consent Form as well as the date on which consent was acquired. Furthermore, the explainer shall enter the date the explanation was provided in the Written Explanation and the Consent Form, and sign or affix his/her name and seal. If a Clinical Trial Collaborator provides a supplementary explanation, he/she should also enter the date of providing the explanation in the Written Explanation and Consent Form, and sign or affix his/her name and seal. The investigator should attach the original Written Explanation and Consent Form signed or stamped and dated as described above to the original medical record, such as the Case Report Form (if there are provisions for retention at the medical institution, the investigator should follow them ) and keep them, and give a copy to the subject.

　If the temperature stimulation test is used as clinical trial data before consent is obtained, it should be used within 3 years from before consent is obtained.

## When important information affecting the subject's decision is obtained (Revision of the Written Explanation and Consent Form)

　When new important information that may be relevant to the subject's consent (information that usually requires revision of the Written Explanation and Consent Form) is obtained, the investigator shall immediately communicate the information to the subject, confirm the subject's willingness to continue the trial, and record in writing that the subject has been informed. In addition, the investigator, with the cooperation of the sponsor, shall promptly revise the Written Explanation and Consent Form based on the said information and obtain approval from the IRB before providing explanations to the subject.

The investigator must explain the clinical trial again using the revised Written Explanation and Consent Form, and obtain the subject's free and voluntary re-consent in writing for continued participation in the clinical trial.

The investigator shall give the subject a copy of the revised Consent Form and Written Explanation with fresh entries of the name, seal, or signature, and date. The original Consent Form shall be retained at the medical institution. The investigator should record that he/she handed a copy of the revised Consent Form and the Written Explanation to the subject.

# Subject Enrollment

## Preparation of a subject screening name list

The investigator shall prepare a subject screening list for all the subjects from whom consent was obtained, including the subject identification code, subject name, Case Report Form number, date on which consent was obtained, and whether or not the Written Explanation and Consent Form were provided. Of these subjects, information on the completion or discontinuation of the clinical trial will also be documented for those subjects for whom optimal stimulation was considered.

## Enrollment Procedure

All subjects whose written consent has been obtained and who have been screened and found eligible will be enrolled into the study. A central registration system using subject identification codes will be used, and all subject enrollments will be done on the internet. Provisional and full enrollment will be conducted for this study.

1. Provisional enrollment

All the subjects from whom written consent is obtained and who are screened and found eligible will be provisionally enrolled.

1. Full enrollment

Only subjects with optimal intensity will be enrolled in the study. Subjects with suboptimal intensity will be stopped at this point, and the study will be terminated after confirming safety 2 weeks later.

## Method of randomization and maintenance of blindness

All persons involved in the clinical trial (except those who performed the randomization assignment work for the investigational device) will be blinded to the randomization information in this trial.

The subjects enrolled in the study will be assigned to 2 groups (group A or group B) in a 1:1 ratio by block randomization on the Web. The total trajectory length at screening (<200 cm per 60 s, ≥200 cm per 60 s) and unilateral/bilateral vestibular disturbance will be assigned as the allocation factors. The allocation procedure will be conducted by the allocation manager appointed by the investigator in accordance with the "Procedure for Randomized Allocation of Investigational Devices" to be prepared separately. For Session I and Session II, the results of randomization of each subject in the registration system will be confirmed by a person other than the investigator (or subinvestigator) at the medical institution (called the Allocation Manager). The investigator will inform the Allocation Manager of the optimal intensity measured in the screening period, and the Allocation Manager will give the placebo stimulus or the device set to the optimal intensity (neither the subject nor the investigator can identify the placebo stimulus or the actual stimulus) according to the allocation result. The investigator or subinvestigator will play the set stimulus for 30 s and confirm that it is below the sensory threshold. As a method of confirmation, the patient is asked whether or not he/she perceives noisy GVS-specific stimuli (slightly spaced, continuous stimuli). If above the sensory threshold, continue to collect for subsequent data, but exclude from the main analysis (however, the data that have been measured up to that point can be used). If below the sensory threshold or above the sensory threshold, but the patient does not feel discomfort, the stimulus of that intensity is played continuously for 4 h. The patient is asked to stay in a designated place during the stimulation and not to touch the stimulating electrodes or the stimulator. We also check that the patient is below the sensory threshold at the time of measurement of the endpoints during stimulation, and if the patient exceeds the sensory threshold, we include the data up to the time before that point in the main analysis. The data obtained after the sensory threshold is found to have been exceeded will be used for supplementary analyses. The Allocation Director and the Allocation Manager shall keep the randomization allocation information confidential until the time of key opening.

In addition, even during placebo stimulation, the vestibular electrical stimulator should be set to blink LED lamps in the same way as during actual stimulation, so that the investigators and subjects do not know that the stimulation is a placebo stimulation.

### Key opening procedure

After all Case Report Forms have been completed and the data for analysis have been fixed, the key will be opened by the Investigational Device Allocation Manager.

### Key opening procedures during clinical trials

If a serious adverse event occurs and the investigator deems it necessary to know information on the allocation to ensure the safety of the subject, the investigator may know the allocation information of a subject. If the allocation information of the relevant subject is disclosed, the investigator shall promptly inform the Allocation Manager that the allocation information on that subject has been disclosed. If the allocation information is disclosed, the noisy GVS of the subject shall be discontinued. If the allocation information is disclosed, the investigator shall record the date of disclosure and the reason. After avoiding an emergency situation, the investigator shall inform the head of the medical institution that the allocation information has been disclosed. If allocation information is disclosed individually during the course of the clinical trial, the Allocation Manager will decide on the blinding, handling, and future policy for other cases conducted at that medical institution.

# Observation, examination, and investigation items of the clinical trial and duration of the trial

## Clinical Trial Schedule

Table 3. Clinical trial schedule

Clinical trial period


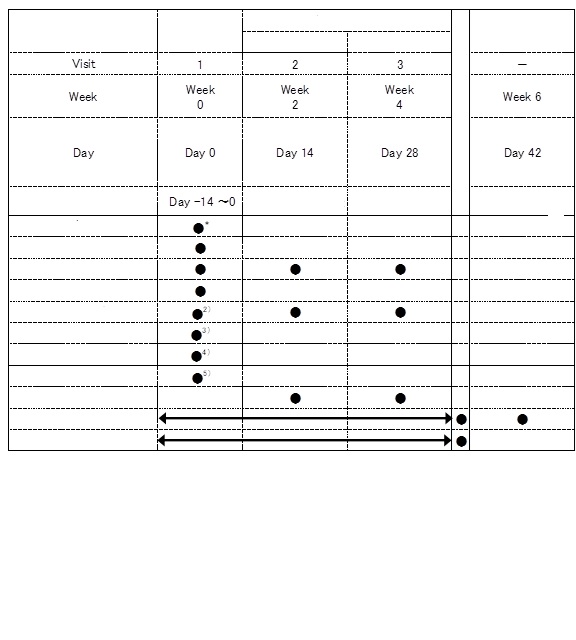


At discontinuation

*: Acquisition of consent is performed before the start of screening, the permitted range is excluded.

**: The body temperature and blood pressure are measured on the day the investigational device is used.

1. Data obtained within 3 years prior to the day of obtaining consent can be used.
2. Measurement related to ‘Patient with total trajectory length of 180 cm in 60 s by the stabilometer when standing with eyes closed’ in inclusion criterion 2 stipulating serious unsteadiness
3. After obtaining the informed consent, the patient who is confirmed to have all the results of the screening test, meets the inclusion criteria, and is not in violation of the exclusion criteria shall be provisionally enrolled promptly.
4. The day of examining the optimal stimulation shall be Day 0.
5. Only subjects with existing optimal stimulation shall be enrolled. Subjects with no existing optimal stimulation shall be discontinued at that time point, and terminated after safety is confirmed 2 weeks later.
6. Defects are examined during use of the investigational device
7. The follow-up examination is performed by phone interview.

Follow-up examination^7)^

Optimum stimulation examination

Subjective symptoms/objective findings**

Permitted range

14-21 days after Visit 3 or day of discontinuation

7-21 days after Visit 2

7-21 days after Visit 1

Concomitant drug/treatment

Adverse event/defect^1)^

Treatment stimulation

Enrollment of subject

Provisional enrolment

Gravitational sway test

Temperature stimulation test^1)^

Subjects’ background

Acquisition of consent

Treatment stimulation period

Phase II

Phase 1

Screening

## Subject Background and Screening Tests

Subjects whose written consent is obtained, who meet the inclusion criteria (selection and exclusion criteria), and who are screened and found eligible will be provisionally enrolled.

1. Subject Background

The subject identification code, gender, date of birth, height, weight, medical history, complications, and concomitant medications (therapies) will be investigated.

1. Subjective symptoms and other findings

Confirm by interview and medical examination (including temperature and blood pressure measurement).

1. Temperature Stimulus Test

2 mL of ice water is injected into the external auditory canal for 20 s to stimulate the lateral semi-circular canal. The stimulation-induced nystagmus is recorded with an electro- or video-nystagmeter to measure the slow phase rate of the nystagmus. After the unilateral injection of water, contralateral stimulation is performed after an interval of at least 5 min.

Unilateral impairment is defined as CP (%)=(maximal slow phase velocity on the healthy side - maximal slow phase velocity on the affected side)/(maximal slow phase velocity on the healthy side + maximal slow phase velocity on the affected side)x100 > 20 %, bilateral impairment is defined as patients with peripheral vestibular impairment with bilateral maximal slow phase velocity of 10 deg/s or less.

1. Stabilometry

The gravity sway is measured for 60 s with eyes open and standing on a stabilometer without placing a foam rubber (without blocking somatosensory input). Subsequently, the subject stands with closed eyes, and after the body sway has reached a steady state, the center of gravity sway is measured for 60 s.

Severe unsteadiness is defined as a total trajectory length of 180 cm or more in 60 s on the stabilometer during closed-eye standing.

- 1. **Examination of optimal intensity**

The optimal intensity with noisy GVS for each provisionally registered subject is determined. The optimal intensity study schedule is shown in Table 4.

Check for adverse events before, during, and after stimulation, and failures during stimulation for the consideration of optimal stimulation.

Subjects for whom the optimal intensity has been determined are "enrolled". On the other hand, the subjects for whom the optimal intensity does not exist will be stopped at this point, and the study will be terminated after confirming safety 2 weeks later.

Table 4: Optimal intensity study schedule

Timing


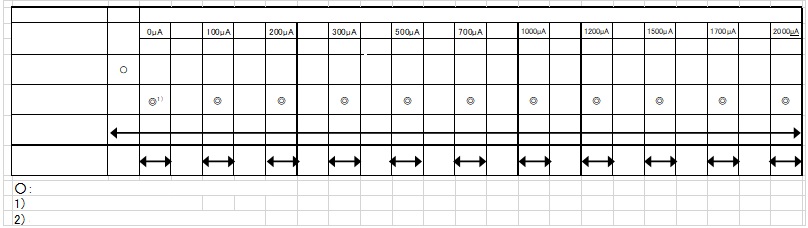


Defects examined during use of the investigational device.

Three measurements performed at 2 min intervals.

Performed before the noisy GVS,⊚performed during the noisy GVS.

Adverse events

≥ 2 min

≥ 2 min

≥ 2 min

≥ 2 min

≥ 2 min

≥ 2 min

≥ 2 min

≥ 2 min

≥ 2 min

≥ 2 min

Before stimulation

Defect^2)^

Gravitational sway test

Subjective symptoms/

Objective findings

Stimulus intensity/duration

### Special note on stimulation

Check before stimulation begins:

Stimulation will not be performed in subjects with acute illness, with a body temperature of 38 °C or higher (febrile phase), with abnormal blood pressure (180 mmHg or higher), or who have consumed alcohol after 22:00 on the day prior to the clinical trial. In such cases, the schedule will be readjusted within acceptable limits. If readjustment is not possible, the study will be terminated.

### Stimulus type

**Stimulus to be tested:**

Optimal intensity for each subject: Among 10 stimulus intensities of currents 100, 200, 300, 500, 700, 1,000, 1,200, 1,500, 1,700, and 2,000 μA, the current intensity below the sensory threshold (the lowest current intensity at which the stimuli specific to noisy GVS (slightly spaced, continuous stimuli) were felt and at which the total trajectory is the current intensity below 1,000 µA that most improved the total trajectory length in the stabilometry among the multiple current intensities that shortened the length.

When interrupted by the subject's complaint, the optimal intensity is determined from the stimuli that have been performed up to that point.

**The Control Stimulus:**

As placebo stimulation, stimulation is performed at a current of 0 μA.

[Rationale for setting the stimulation current]

In 2015, we conducted an investigator-initiated clinical trial to examine the effects of 3-h stimulation in healthy elderly subjects and the sustained effects during and after stimulation, and found that there was an improvement in the total trajectory length during stimulation and up to 4 h post-stimulation. In other studies using noisy GVS, there were several studies that stimulated at intensities up to 2,000 µA and no adverse events were reported. Therefore, we set the upper limit for examining the sensory threshold at 2,000 µA and the upper limit for optimal stimulation at 1,000 µA, which has been proven for long-duration stimulation ^10-12)^.

- - 1. **Stimulation method**

**Optimal intensity consideration:**

To determine this optimal intensity, stabilometry is performed. In multiple continuous current intensities, the total trajectory length is shortened to below the sub-threshold sensitivity (the minimum current intensity where the noisy GVS-specific stimulus (shortly spaced continuous stimuli) is felt). A patient who shows an improvement of at least 10% in the current intensity with the best improved trajectory length among those multiple continuous current intensities when compared with the total trajectory at baseline (the average of three measurements during closed-eye standing with no current) was defined as a patient with existing optimal intensity and the ‘enrolment of the patient in this study’ is performed.

The optimal intensity is defined as the current that improves the total trajectory length the most among the above multiple currents.

Procedures for examining the optimal intensity

1) The subject stands with closed eyes with no foam rubber placed (without interrupting somatosensory input) and no electric current applied to the stabilometer, and after the body sway reaches a steady state, the gravimetric sway is measured for 30 s.

2) Perform the measurement 1) three times with an interval of 2 min.

3) After the measurement, after an interval of 2 min of descending from the gravimeter, measure the center of gravity sway for 30 s in the same manner as above when a current of 100, 200, 300, 500, 700, 1,000, 1,200, 1,500, 1,700, or 2,000 μA is applied.

The optimal intensity is the current intensity that improves the total trajectory length the most among several consecutive current intensities that improve the total trajectory length below the sensory threshold in six stimulus intensities from 100 µA to 1,000 µA. When the subject complains of the interruption, the optimal intensity is determined from the stimuli that have been performed up to that point. The current intensity is increased up to a maximum of 2,000 μA to determine whether or not pain is felt. However, the test is terminated when the subject feels pain. The minimum current intensity at which the subject feels a stimulus specific to the noisy GVS is used as the sensory threshold.

The adverse events before, during, and after the stimulation for consideration of optimal stimulation, and failure checks during stimulation are noted.

The subjects for whom the optimal intensity has been determined are "enrolled". On the other hand, the subjects for whom the optimal intensity does not exist will be stopped at this point, and the study will be terminated after confirming safety after 2 weeks.

### Therapeutic stimulus

Noisy GVS ("optimal stimulation for each subject" or "placebo stimulation") assigned to the subject in question will be performed on the subjects enrolled in this study. Two weeks after this enrolment, "Session I" stimulation will be performed to assess the efficacy and safety. After the evaluation of Session I, "Session II" stimulation will be performed after a period of at least 7 days, and the efficacy and safety will be evaluated in the same manner.

Table 5. Schedule for examining treatment and carryover effects


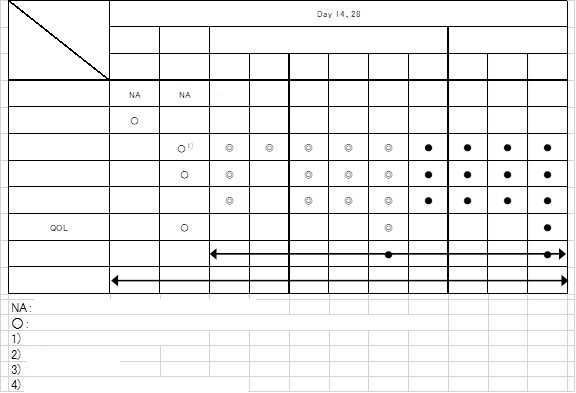


Defects examined during use of the treatment device

Performed whenever possible

Measured with an attached pedometer

Performed before noisy GVS, ⊚: performed during noisy GVS, ⦁: performed after noisy GVS

Three measurements at an interval of 2 min

Not applicable. *: Start of stimulation is the starting point

Adverse events/defect^4)^

Evaluation of subjective improvement level

Subjective symptoms/ objective findings

Amount of activity

Gait function test

Gravitational sway test

Permitted range

+ 15 min

+ 15 min

+ 15 min

+ 15 min

+ 15 min

+ 15 min

+ 15 min

+ 15 min

+ 15 min

1 h

7 h

6 h

5 h

4 h

3 h

2 h

30 min

Immediately

after

Before

≥ 2 min^*^

After stimulation

Noisy GVS

Immediately before stimulation

Before stimulation

Item

Timing

### Treatment Stimulation Method

The optimum stimulation current or the placebo stimulation current (0 μA) determined for each subject is applied continuously for 4 h. During the stimulation, the subjects are asked to remain in a designated area and not to touch the stimulating electrodes or stimulator.

### Endpoints

In Session I and Session II, the endpoints will be implemented according to the schedule shown in Table 3.

#### Stabilometry

Before, during, and after the stimulation, the center-of-gravity sway is measured on a stabilometer with no foam rubber placed, and for 30 s after the body sway reaches a steady state.

Ten measurements are taken immediately before the stimulus, immediately after the start of noisy GVS, 30 min, 1 h, 2 h, 3 h, 4 h, 5 h, 6 h, and 7 h later, respectively.

If the patient loses balance significantly during the stabilometer measurement and is unable to maintain the posture with both upper limbs in contact with the body side, or falls off the stabilometer, the measurement should be performed again.

#### Gait function tests (performed when possible)

The following measurements are performed 9 times immediately before stimulation, immediately after the start of noisy GVS, 1 h, 2 h, 3 h, 4 h, 5 h, 6 h, and 7 h later, according to the separately prescribed procedures.

1. The walking speed, stride length, step time, and left-right sway width during a 10-m walk were measured using a gait analyzer (WALK-MATE LAB Corporation, Walk Mate Viewer)
2. Score of DGI (Short Form) (Appendix 1)

#### Subjective Improvement Assessment

Immediately after the start of, and 1 h, 2 h, 3 h, 4 h, 5 h, 6 h, and 7 h after the start of noisy GVS, the subjects themselves are asked to evaluate in 5 steps of "1: improved, 2: slightly improved, 3: unchanged, 4: slightly worse, 5: worse" compared with the state before stimulation. The test is not conducted immediately before the stimulation.

#### Quality of life

The Japanese version of mFES and Japanese version of DHI will be used immediately before the stimulation and 3 and 7 h after the start of noisy GVS.

#### Activity level

The amount of activity (number of steps) immediately after the start of noisy GVS to 3 h and 7 h after the start of noisy GVS will be measured using a pedometer.

## Restricted concomitant medications and prohibited concomitant therapies

Implementation period

From the time consent is obtained to the time of week 4 or discontinuation

[Details of implementation]

No new use of anxiolytics, antidepressants, sleeping pills, dizzying agents, or drugs indicated for dizziness from the time of obtaining consent until week 4 or discontinuation. Medications that have been continuously administered for at least 8 weeks starting from the date of the optimal stimulation study may be used, provided that the dosage and administration are not changed if symptoms are stable. In addition, new rehabilitation for the purpose of improving unsteadiness is prohibited from the time of obtaining consent until week 4 or discontinuation. Rehabilitation that has been ongoing for at least 8 weeks starting from the date of the optimal stimulation study should be continued, provided that the content is not changed if the symptoms are stable.

## Concomitant medications/ therapy

Implementation period

From the time consent is obtained to the time of week 4 or discontinuation

[Details of implementation]

Medications or therapies used from the time consent is obtained to the time of week 4 or discontinuation will be investigated. For concomitant medications, the name of the drug, duration of administration (in the case of abortive use, if the date of drug use is known, collect information on the date of drug use whenever possible), and the purpose of use should be investigated and included in the case report form.

## Safety evaluation

Implementation period

For adverse events, from the time of obtaining consent to week 6 (end of follow-up) or discontinuation, and for defects, at the time of using the investigational device

[Details of implementation]

Screening and treatment stimulation period:

Subjective symptoms/other findings will be confirmed by medical examination prior to stimulation. The adverse events and malfunctions will be confirmed before, during, and after stimulation.

Safety follow-up:

Two weeks after the end of the evaluation of "Session II" of the treatment stimulation period, the patients will be interviewed about the occurrence of adverse events.

### Definition of adverse events and defects

Adverse Event" means any undesirable or unintended illness or disorder and its symptoms (including abnormal laboratory values) that occur in a subject, user, or other person (hereinafter referred to as “Subject,” (2) The term "Unintended Diseases" shall include all undesirable or unintended diseases or disorders and their signs (including abnormalities in clinical laboratory test values) that occur in subjects and other persons (hereinafter referred to as “Subjects”. However, the occurrence of such symptoms in a person other than the subject shall be limited to those suspected to be caused by the use of the investigational device.

Failure means any failure of the investigational device in terms of quality, safety, performance, etc., including breakage or malfunction, whether due to design, delivery, storage, or use.

(2) In the event of an adverse event or failure of the investigational device or subjects, the investigator shall immediately take appropriate measures to ensure the safety of the subjects and to report the event. The results shall be recorded in the Case Report Form.

### Duration, frequency, and methods to examine adverse events/defects and serious adverse events/defects

The investigator or medical institution personnel are responsible for detecting, recording, and reporting adverse events or events that meet the definition of a serious adverse event.

Adverse events should be collected from the time of obtaining consent to the time of follow-up or discontinuation of the study. The collection period for failure is from the time of use of the investigational device.

1) Adverse events

The investigator shall record the following items regarding the adverse events that occur:

- Causal relationship with noisy GVS (no causal relationship with noisy GVS, a causal relationship with noisy GVS cannot be ruled out)
- Time (date (hour)* of onset, date of conversion, or date of confirmation of conversion)
- *: Time will be recorded only for adverse events during the use of the investigational device.
- Severity (severe, moderate, mild)
- Mild: Adverse events that are easily tolerated, cause only slight discomfort, and do not interfere with daily life
- Moderate: Unpleasant adverse event that interferes with the daily life
- Severe: Adverse event that prevents the patient from leading a normal daily life
- Severity (severe, non-serious)
- Status of Noisy GVS discontinuation
- Treatment
- Outcome of an adverse event (recovered, mildly recovered, not recovered, with sequelae, died, unknown)

2) Defect

When a defect occurs, the investigator (or subinvestigator) shall record the following items related to the defect

- Status of the risk of a health hazard
- Details of the defect
- Date and time of onset
- Severity of incident
- Predictability

### Definition of serious adverse events

Serious adverse events are defined as any of the following among adverse events

(1) Event resulting in death

(2) Life threatening event

(3) Event requiring hospitalization or extended hospitalization for treatment

(4) Permanent or significant disability or disorder

(5) Event resulting in the detection of congenital anomalies

(6) Other serious medical events

Other situations, i.e., events that are not immediately life-threatening or result in death or hospitalization, but are serious enough to put the subject at risk or require treatment to avoid the consequences described above, should also be considered as serious.

### Response to serious adverse events and defects

If a serious adverse event or a defect with the potential to cause a serious adverse event (hereinafter referred to as "defect etc.") occurs during the clinical trial period, the coordinating investigator shall handle the information on the relevant defect, in accordance with the "Procedures for Handling Safety Information".

The coordinating investigator shall make a decision on whether or not to report to the Minister of Health, Labour, and Welfare (the "Authority") in accordance with the "Procedures for Handling Safety Information".

If it is judged that a report to the authority is necessary, the investigator who conducts the clinical trial on his/her own shall prepare a report to the authority and submit it to the Pharmaceuticals and Medical Devices Agency (PMDA) within the reporting deadline in Table 4.

The coordinating investigator shall share the information with investigators and the investigational device provider at each medical institution in accordance with the "Procedures for Handling Safety Information".

Table 6. Serious adverse event reporting deadlines

|  | Unforeseen events | Foreseen events |
| --- | --- | --- |
| Event resulting in death  Event that is life-threatening | Seven days  Periodic report every year | Fifteen days  Periodic report every year |
| Event requiring hospitalization or extended hospitalization for treatment  Event resulting in permanent or significant disability or disorder  Event leading to detection of a congenital anomaly | Fifteen days  Periodic report every year | Periodic report every year |
| Defect that may lead to a serious adverse event | Thirty days  Periodic report every year | |

### Expected adverse effects

The adverse events reported in the clinical studies conducted to date were cerebral infarction, dizziness, and right-sided hearing loss. Of these, there were no adverse events (adverse effects) for which a causal relationship with the noisy GVS could not be ruled out. The event of equipment failure was "fast lighting of the power ON/BATT LED of the main body of the stimulator".

Anticipated Defects

- If the patient is very sensitive to electrical stimulation, the electrical stimulation may cause pain.
- If the vestibular nerve is very sensitive, symptoms, such as dizziness, nausea, headache, and the possibility of falling while standing or walking may occur.
- Individuals with allergies may develop hives or dermatitis to the surface electrode applied to the back of the ear.
- Failure of the electrical stimulator may result in interruption of the electrical stimulation or under- or over-current flow than the programmed value.
- If the power switch of the main body of the stimulator is turned from ON to OFF and back ON again in less than 3 s, the LED may blink fast and switch off the electrode, light up constantly, or become unlit, causing the output to stop.

# Data Analysis and Statistical Considerations

Details of the tabulation and analysis will be described in the "Statistical Analysis Protocol". Any changes from the analyses described in this study protocol will be described in the "Statistical Analysis Protocol," "Statistical Analysis Report," and "Summary Report".

If this clinical trial is terminated during the course of the study, all the available data will be listed and appropriate statistical analyses will be performed.

## Study design considerations

### Setting of the number of cases

The target number of patients for this clinical trial will be set at 60 (50 enrolled in the main study and 40 completed).

### Rationale for setting the number of cases

In a previous study^6)^, the total trajectory lengths of healthy subjects, patients with unilateral peripheral vestibular impairment, and patients with bilateral peripheral vestibular impairment were 1.69, 2.08, and 2.17 cm (per second), respectively. Since an improvement of approximately 19% in patients with unilateral peripheral vestibular disorder and 23% in patients with bilateral peripheral vestibular disorder would be consistent with normal subjects, we consider that clinical significance would exist if there is an improvement in the total trajectory length of at least 10%, equivalent to approximately half of the 19% and 23% improvement between the placebo period and the use of this experimental device.

The Type 1 error and detection power in 1000 simulations are shown in the table below, assuming the following [1] for the total trajectory length data, based on the investigator-initiated clinical trial data. The target number of patients is set to 60 (50 enrolled and 40 complete), assuming a dropout rate of 20% and a suboptimal intensity of 15%.

Table 7. Relationship between the number of cases and detection power (%)

| Effect of treatment^*1^ | N=30 | N=40 | N=50 |
| --- | --- | --- | --- |
| 0% ^*2^ | 4.7 | 5.2% | 4.7 |
| 5% | 31.5%. | 38.2% (in the case of the | 46.1% (in the case of the |
| 7% | 55.0 | 70.6% | 78.2 |
| **10%** | **84.1%** | **91.2%** | **96.1%** |

*1 Rate of improvement in the GVS period relative to the placebo period.

*2 Rows with 0% treatment effect represent Type 1 error. The nominal significance level of 5% was confirmed to be ensured.

[1] In the investigator-initiated clinical trial data, the greatest improvement occurred immediately after stimulation (23% in Session II and 19% in Session III), and improvement rates of more than 12% existed up to 3 h later (see the Table below). To be somewhat more conservative than the investigator-initiated clinical trial data, the standard deviation of the improvement rate of the total trajectory length in the GVS and placebo periods was set to 30% and the correlation coefficient of the improvement rate between each time point to 0.3. In addition, the simulated improvement rate data were generated from a multivariate truncated normal distribution cut in the range [-80, 80]. The magnitude of the treatment effect was assumed to be constant throughout the 3 h and varied from 0%, 5%, 7%, and 10%.

Table 8. Descriptive statistics for the percentage improvement in the total trajectory length in exploratory clinical studies as well as the correlation coefficient (%) between the rate of improvement immediately after stimulation and the rate of improvement at each time point

|  |  | Average | Standard deviation | Minimum value | Maximum value | Correlation coefficient |
| --- | --- | --- | --- | --- | --- | --- |
| Session II | |  |  |  |  |  |
|  | Immediately after stimulation | 23.1% | 14.7 | 3.1% | 49.0 | 1.00 |
|  | 30 min later | 17.1% | 14.1% | -14.3 | 44.3% | 0.49 |
|  | One h later | 16.5%. | 18.0%. | -21.1% | 52.3 | 0.53 |
|  | Two h later | 17.1% | 15.0%. | -6.3%. | 41.2 | 0.30 |
|  | Three h later | 14.8 | 11.1% | -0.9%. | 32.3 | 0.62 |
| Session III | |  |  |  |  |  |
|  | Immediately after stimulation | 19.2% | 23.6 | -25.2 | 69.5%. | 1.00 |
|  | 30 min later | 17.0%. | 22.7 | -22.5%. | 70.0 | 0.84 |
|  | One h later | 14.8 | 21.5%. | -13.1% | 70.1% | 0.74 |
|  | Two h later | 12.6%. | 25.5%. | -31.9%. | 61.3% | 0.70 |
|  | Three h later | 14.0% (1.0) | 26.3 | -43.5%. | 57.2 | 0.82 |

### Re-estimation of the number of subjects

A re-estimation of the number of subjects has not been planned for this study.

## Consideration of data analysis

### Completed case

A subject who has completed all stimuli and tests specified in the study protocol and has completed post-hoc (additional and follow-up) investigations is considered to have completed the study.

### Analysis sets

Full Analysis Set (FAS) for efficacy

Of the enrolled patients in the study, the full analysis set will consist of patients who visited the hospital in either Session I or Session II, were fitted with the investigational device, and had at least one efficacy endpoint measured. However, the following patients will be excluded:

- GCP non-compliance: Patients who have not given appropriate informed consent

Per Protocol Set (PPS)

From the FAS, the following subjects will be excluded from the PPS.

- Subjects not meeting the selection criteria
- Subjects that violated the inclusion and exclusion criteria
- Subjects who administered concomitant prohibition therapy
- Subjects who did not visit the sites in both Session I and Session II
- Other subjects with serious protocol deviations

Safety Analysis Set (SAS)

Of the enrolled patients, the SAS shall consist of those patients in whom at least one safety endpoint was evaluated.

If a subject is excluded from the analysis, the details and reasons should be described in the Clinical Study Report.

### Interim analysis

　No interim analysis will be performed in this trial.

### Efficacy Analysis Methods

Primary analysis for the primary endpoint

The primary endpoint (percent change from baseline in total trajectory length) during stimulation (immediately after noisy GVS initiation to 3 h later) will be used as the response variable, and timing (Session I, Session II), treatment device (placebo, noisy GVS), time point, interaction between treatment device and time point, and allocation factors (total trajectory length at screening (per 60 s (<200 cm, ≥200 cm), unilateral/bilateral vestibular impairment) as the explanatory variables and fitting a mixed-effects model with individual effects as a variable effect. The least-squares mean of the treatment effect throughout the stimulation is calculated, and the *P* values are calculated for the 95% confidence interval and the null hypothesis that the treatment effect is 0%. The Kenward–Roger method is used to calculate the degrees of freedom.

　If the sensory threshold is found to be exceeded, a decision on whether to accept or reject the data thereafter will be made at the Case Review Committee.

The point estimates and 95% confidence intervals should be obtained for the least squares means of the rates of change by treatment device and time point calculated by the model above. The trends over time are also shown in the Figure.

Secondary analyses for the primary endpoints:

1. The descriptive statistics are calculated for the raw values of total trajectory length, change from baseline, and rate of change at all the time points by allocation group, treatment stimulus period, and time points. Subsequently, the allocation groups are combined, and descriptive statistics are calculated for each treatment device and time point. The descriptive statistics of the difference in the amount and rate of change within individuals are calculated for each time point, and a one-sample t-test is performed to calculate the *P*-value.
2. Corresponding t-tests will be performed for the difference between the mean rate of change in the total trajectory length from immediately after the start of stimulation to 3 h after stimulation in the GVS period and the mean rate of change in the total trajectory length from immediately after the start of stimulation to 3 h after stimulation in the placebo period.
3. Inference on treatment effects using Bayesian flow methods: A mixed-effects model was fitted with the rate of change in the total trajectory length during stimulation as the response variable, the allocation group, treatment instrument, time point, interaction between treatment instrument and time point, and baseline values as explanatory variables, and individual effects as variable effects to obtain the posterior distribution of least squares means for the treatment effect. The prior distribution is Jeffrey’s uninformed prior distribution, and the posterior probability that the improvement in the GVS period is at least 5%, 10%, or 15% greater than the improvement in the placebo period is calculated. The 95% and 90% confidence intervals for the posterior probabilities are also calculated.
4. A mixed-effects model is fitted with the rate of change in the total trajectory length during stimulation in the GVS period as the response variable and baseline values as the explanatory variables to obtain a posterior distribution of the least-squares mean of the rate of change averaged over time points. The prior distribution is calculated as the posterior probability that there is at least 5%, 10%, 15%, and 20% improvement in the GVS period for the prior distribution obtained from Jeffrey’s uninformative prior distribution and the data up to 3 h after the second period of stimulation in the investigator-initiated clinical trial 3. The 95% and 90% credit intervals of the posterior probabilities will also be calculated.
5. To confirm the validity of the simulation experiment setting in the sample size estimation, correlation coefficients between the rates of change in the total trajectory length during stimulation will be calculated for each treatment device and time point.
6. The same model as in the main analysis for the primary endpoint will be used for the change from the baseline in the total trajectory length during stimulation.
7. The same analyses as the primary and secondary analyses (1) and (2) for the primary endpoint will also be performed using data after the sensory threshold is found to have been exceeded.

Analysis for secondary endpoints

- Analysis for Stabilometry

1. Descriptive statistics will be calculated for the percent change from baseline in the perimeter area and RMS values at all time points by allocation group, treatment stimulus period, and time point. Subsequently, the allocation groups are combined and descriptive statistics are calculated for each treatment device and time point. The descriptive statistics of the difference in the rate of change within individuals will be calculated at each time point, and a one-sample t-test will be performed to calculate the *P*-value.
2. For the rate of change in the total trajectory length, perimeter area, and RMS value in all time points, the mean rate of change and change over time in 95% confidence interval by treatment device and time point will be illustrated.

- Analysis of gait function tests

1. Descriptive statistics will be calculated for the percentage change from baseline in the gait test endpoints (DGI score, walking speed at 10-m gait, stride length, step time, and left-right sway) at all time points by allocation group, treatment stimulus period, and time point. Next, the allocation groups will be combined and descriptive statistics will be calculated for each treatment device and time point. The descriptive statistics of the difference in the rate of change within individuals at each time point will be calculated for each time point for the whole group and for each group, and a t-test between groups will be performed to calculate the *P*-value.
2. The same model as the main analysis for the primary endpoints will be used for the percent change in the perimeter area, RMS values, and gait test endpoints (DGI score, walking speed at 10 m gait, step length, step time, and left-right sway) during stimulation.
3. For the rate of change in endpoints of the gait test in all time points, the mean value of the rate of change and the changes over time in 95% confidence interval by device and time point shall be illustrated.

- Analysis for subjective improvement scores

1. Descriptive statistics will be calculated for subjective improvement scores at all time points by allocation group, treatment stimulus period, and time point. Subsequently, the allocation groups will be combined and descriptive statistics will be calculated by treatment device and time point.
2. With the subjective improvement score during stimulation as the response variable; allocation group, treatment device, and interaction of time point as the explanatory variables; the mixed effect model with individual effect as the random effect is applied. The least squares means of the treatment effects throughout the stimulations is determined, and the 95% confidence intervals and *P*-values are calculated. The Kenward–Roger method is used to calculate the degrees of freedom.
3. For the subjective improvement scores in all the time points, the mean value and changes over time in the 95% confidence interval by treatment device and time point are illustrated.

- Analysis of QOL data

1. Descriptive statistics will be calculated for the change from baseline in the mFES score, DHI score, and DHI subscale scores at all time points by allocation group, treatment stimulus period, and time point. Subsequently, the allocation groups will be combined and descriptive statistics will be calculated by treatment device and time point. Regarding the difference in the amount of change within individuals, the descriptive statistics are calculated by time point and a one-sample t-test will be performed to calculate the *P*-value.
2. An analysis of covariance will be performed using the mFES score, DHI score, and DHI subscale scores at 3 and 7 h after the start of stimulation, respectively, as the response variables, and the allocation group, treatment device, and baseline values as the explanatory variables.

- Examination of correlations between endpoints

1. Pearson/Spearman correlation coefficients between the rate of change in total trajectory length at each time point during stimulation and the following variables are calculated for each treatment device.

- Percentage change in gait test endpoints
- Subjective Improvement Score

1. Pearson/Spearman correlation coefficients between the rate of change in total trajectory length and the following variables are calculated for each treatment device at 3 and 7 h after the start of stimulation, respectively.

- mFES Score
- DHI Score
- Scores for each subscale of DHI
- Number of steps
- Calculation of posterior probabilities

1. For each secondary endpoint, as in the secondary analysis (3) for the primary endpoint, a Bayesian flow technique was used to set the prior distribution as Jeffreys uninformed prior distribution, and the posterior probability that the improvement in the GVS period was at least slightly better than improvement in the placebo period, greater than 5% (0 or 1 on the subjective improvement score) to calculate the 95% credit interval.

### Safety analysis Methods

　The safety analysis set will be used for the safety analysis. During the analysis of safety data, tabulations and lists of data will be prepared and no statistical tests will be performed.

#### Adverse events

　Adverse events will be coded according to the ICH International Pharmacovigilance Dictionary (MedDRA), classified by organ category (SOC) and basic term (PT), and tabulated by stimulus.

　A separate list of deaths, serious adverse events, or adverse events resulting in discontinuation and other significant adverse events will be prepared.

#### Investigational device defect

　Defects of the investigational device shall be aggregated by event.

# Clinical Trial Quality Management and Quality Assurance

## Regulatory and ethical considerations, including procedures for obtaining consent

　This clinical trial will be conducted in compliance with Good Clinical Practice (GCP), the standards set forth in Article 14-3 and Article 80-2 of the Pharmaceutical Affairs Law, all applicable subject privacy protection requirements, and the Declaration of Helsinki (including its latest revision). This includes, but is not limited to, the following:

Review and approval of the study protocol and subsequent revisions by the Institutional Review Board (IRB)

Subject Consent

Reporting Requirements of the Principal Investigator

## Notification of the Clinical Trial Protocol to the Regulatory Authorities

　The coordinating investigator shall submit a notification of the clinical trial protocol to the regulatory authority in accordance with Article 80-2 of the Pharmaceuticals and Medical Devices Act.

## Deviations and amendment to the clinical trial protocol

### Deviation from the clinical trial protocol

　In order to avoid immediate risk to subjects, the investigator (or subinvestigator) may deviate from the study protocol without prior written agreement from the coordinating investigator and prior approval from the IRB. In case of such deviation, the investigator shall record the details of and reasons for all deviations, promptly obtain approval from the head of the medical institution and IRB, and obtain agreement in writing from the coordinating investigator via the head of the medical institution.

　In addition, the investigator (or subinvestigator) shall record all the deviations from the study protocol. Only for deviations made to avoid immediate risk to subjects, the investigator shall prepare a record explaining the reasons for the deviation and submit it to the coordinating investigator and the head of the medical institution, and retain a copy of the record.

### Amendment to the Clinical Trial Protocol

1. If a major amendment to the study protocol (e.g., a change that increases the risk to subjects or affects the conduct of the study) becomes necessary, the person who conducts the study on his/her own (investigator coordinator) shall, in consultation with the investigator, promptly record all changes and the reasons in writing, revise the study protocol, and notify the head of the medical institution and the investigator of the changes to the study protocol (and consent and other sample explanatory documents as necessary). The coordinating investigator shall revise the protocol and notify the head of the medical institution and the investigator of the amendments to the protocol (consent document and other sample Written Explanation, if necessary). The investigator shall not implement any major amendments before obtaining approval from the IRB.
2. If any amendments that do not fall under 1) above become necessary, the person who coordinating investigator shall, in consultation with the investigator, record all changes, and the reasons for them in writing and notify the head of the medical institution and the investigator. All such amendments must also be approved in advance by the IRB, except in cases where emergency risk is to be avoided.

## Quality control

The coordinating investigator shall conduct quality control of the clinical trial in accordance with the standard operating procedures for conducting clinical trials, the standard operating procedures established prior to the clinical trial, and the procedures for conducting monitoring in this clinical trial.

### Monitoring

　The coordinating investigator shall have the investigator conduct monitoring to confirm that the clinical trial is being conducted in accordance with the Declaration of Helsinki and in compliance with the GCP of the Pharmaceutical Affairs Law and the protocol, and that the trial data are accurate and complete and can be verified against the clinical trial-related records, such as source documents. The coordinating investigator shall appoint the monitors for the clinical trial after confirming that they meet the requirements for the monitors.

The monitors shall conduct the following through monitoring of the site, investigators, subinvestigators, collaborators, investigational device managers, and other parties involved in the conduct of the clinical trial.

1. Investigate the status of obtaining consent from subjects, management of investigational devices, and progress of the clinical trial (including discontinuation and adverse events), and confirm that the clinical trial is being conducted in accordance with GCP, this protocol, and other agreed documents between the coordinating investigator, the head of the medical institution, and the investigators.
2. Collect and provide the information necessary to appropriately conduct clinical trials (information on safety, efficacy, quality related to the investigational device).
3. Confirm that the site and investigators meet the requirements required to properly conduct the clinical trial and that these requirements are maintained throughout the clinical trial period. Also, confirm that the medical institutions, including laboratories and necessary equipment and staff, are sufficient to conduct the clinical trial safely and appropriately, and that they will continue to be so throughout the clinical trial period.
4. Ensure that all clinical trial-related records, including the source documents, are created and maintained to be accurate, complete, and up-to-date.
5. Confirm that the respective custodians are keeping the essential documents to be kept at the medical institutions participating in the study.
6. Cross-check the contents of Case Report Forms with all clinical trial-related records, including the source documents, to ensure that they are accurate.

　The investigator and the medical institution must allow the monitor direct access to the source documents and other materials related to the study.

　Direct inspection by monitors and identification of source documents shall be separately stipulated in the "Monitoring Procedures" specific to the relevant clinical trial.

### Data management

Data quality control is conducted by monitoring and data management in accordance with the standard operating procedures established prior to the clinical trial by the coordinating investigator conducting the clinical trial.

Data management will identify and implement the most effective data collection and management methods for the study protocol and present data sets that meet the objectives of the study protocol.

The subject data will be entered into an electronic Case Report Form designated by the investigator and sent electronically to the coordinating investigator, and will be matched with other data in a validated data management system.

Data management is performed in accordance with applicable data cleaning procedures and is intended to ensure data integrity, including the removal of data errors and inconsistencies. Adverse event terms and concomitant drug names are coded using the latest version of MedDRA (ICH International Glossary of Pharmaceutical Terms) and the drug name data file, respectively.

The electronic Case Report Forms (including queries and audit trails) will be retained by the coordinating investigator and a copy will be sent to the investigator and retained as the investigator's copy. In no event will subject initials be collected and reported to the coordinating investigator.

In addition, the case report forms will not be prepared for subjects who did not use the investigational device, such as subjects deemed ineligible to participate in the clinical trial.

## Quality assurance

　The coordinating investigator shall have an independent person from the department related to the clinical trial, including the department in charge of monitoring, conduct the audit to ensure that the clinical trial is conducted and that data are prepared, recorded, and reported in compliance with GCP, all applicable regulatory requirements, the clinical trial protocol, and the procedures for conducting the clinical trial. The audit shall be conducted by a person independent of the departments related to the clinical trial, including the department in charge of monitoring.

The IRB and regulatory authorities may also conduct investigations at any time during or after the clinical trial.

When an audit or investigation is conducted, the coordinating investigator, investigator, and the head of the medical institution shall agree that the Audit Manager and the investigator can directly inspect all the relevant documents, and that the investigator (or sub-investigator) and collaborators, etc. will cooperate in conducting an appropriate audit and investigation.

## Discontinuation of the clinical trial at the medical institution

　The coordinating investigator may temporarily suspend or prematurely discontinue the clinical trial at any time for reasons, including safety or ethical issues or serious non-compliance. If the coordinating investigator determines that such action is necessary, he/she will discuss the suspension or discontinuation of the clinical trial and the reasons for such action with the investigator and the director of the medical institution (if applicable). If possible, the coordinating investigator will report to the investigator or the head of the medical institution before suspending or discontinuing the clinical trial.

　When a clinical trial is suspended or discontinued for safety reasons, the coordinating investigator shall immediately report to all the investigators, the head of the medical institution (if applicable), and/or the medical institution. In addition, the self-initiating investigator will immediately report to the regulatory authorities the interruption or discontinuation of the clinical trial and the reasons for the interruption or discontinuation. If required by applicable regulations, the investigator or the head of the medical institution will immediately report to the IRB and explain the reason for the interruption or discontinuation.

## Record Keeping

　After completion of the clinical trial, the investigator or the head of the medical institution must keep all the clinical trial records in a safe place. These records must be easily retrievable in the event of an audit by the coordinating investigator or an investigation by the regulatory authorities.

　The coordinating investigator shall inform the investigator of the length of time the institution's records will be retained to ensure compliance with all applicable regulatory requirements. The minimum retention period shall be in accordance with the longest standard applicable to the medical institution in principle among the laws/regulations of each country, the coordinating investigator’s standard operating procedures, and/or the requirements of the medical institution.

　The investigator will notify the coordinating investigator of any agreement on the long-term retention of records at a site other than the site where the clinical trial is conducted, or transfer of ownership of records when the investigator moves out of the medical institution.

　The person in charge of the archiving of materials required to be kept at the site under GCP, such as 　subject medical records, laboratory data, IRB records, contracts, subject consent records, and investigational device management records related to this clinical trial, must be designated by the site and must retain them until the later of the following 1) or 2) below, and the person in charge of storage shall keep them until the later of the following dates. However, if the investigator requires a longer retention period, the period and method of retention shall be discussed with the coordinating investigator. When storing records, the storage manager of each record shall be designated and each record shall be stored. When there is no longer a need for retention, the head of the medical institution shall be notified in writing by the coordinating investigator.

1. The date on which five years have elapsed from the date of approval of manufacture and marketing for the investigational device concerned (the date on which three years have elapsed from the date of notification of the decision to discontinue the development or the decision not to attach the materials on clinical trial results to the application)
2. 3 years after the discontinuation or termination of the clinical trial

## Provision of clinical trial results and information to investigators

　If required by applicable regulatory requirements, the investigator shall approve the Clinical Study Report by signature. The investigator will be provided with statistical charts and relevant reports as appropriate and the opportunity to review the results of the overall clinical trial with the coordinating investigator or at a mutually agreed upon location.

　After completion of the Clinical Study Report, the coordinating investigator will disclose the results of the clinical trial through JAPIC and provide the investigator with a full summary of the clinical trial results. The investigator will inform the subjects of the summary of the trial results as appropriate.

## Clinical Trial Period

December 2018 to June 2022 (from the date of obtaining consent for the first subject to the end of the trial for the last subject)

# Clinical Trial Implementation System

As shown in the Appendix.

# References

1. Neuhauser HK, von Brevern M, Lezius F, Fedomannn M, Ziese T, Lempert T. Epidemiology of vestibular vertigo: A neurotologic survey of the general population. Neurology 2005; 65: 898-904.
2. Agrawal Y, Carey JP, Della Santina CC, Schubert MC, Minor LB. Disorders of balance and vetibular function in US adults: data from the National Health and Nutritional Examination Survey, 2001-2004. Arch Intern Med 2009; 169: 938-944.
3. Mulavara AP, Fiedler MJ, Kofman IS, Wood SJ, Serrador JM, Peters B, Cohen HS, Reschke MF, Bloomberg JJ. Improving balance function using vestibular stochastic resonance: optimizing stimulus characteristics. Exp Brain Res 2011; 210: 303-312
4. Flores A, Manilla S, Huidobro N, De La Torre-Valdovinos B, Kristeva R, Mendez-Balbuena I, Galindo F, Trevin˜o M, Manjarrez E. Stochastic resonance in the synaptic transmission between hair cells and vestibular primary afferents in development. Neuroscience 2016; 322: 416-429.
5. Iwasaki S, Yamamoto Y, Togo F, Kinoshita M, Yoshifuji Y, Fujimoto C, Yamasoba T. Noisy vestibular stimulation improves body balance in bilateral vestibulopathy. Neurology 2014; 82: 969-975
6. Fujimoto C, Yamamoto Y, Kamogashira T, et al. Noisy galvanic vestibular stimulation induces a sustained improvement in body balance in elderly adults. Sci Rep 2016; 6: 37575.
7. Document, The University of Tokyo Hospital
8. Clinical Study Report, The University of Tokyo Hospital
9. Shumway-Cook A, Gruber W, Baldwin M, Liao S. The effect of multidimensional exercises on balance, mobility, and fall risk in community-dwelling older adults. Phys Ther. 1997; 77: 46-57.
10. R.GoelaM.J.RosenbergbH.S.Cohenb, et.al. Calibrating balance perturbation using electrical stimulation of the vestibular system. J Neurosci Methods. 2018; 311:193-199
11. Nakamura J, Kita Y, Ikuno K, et. al. Influence of the stimulus parameters of galvanic vestibular stimulation on unilateral spatial neglect. Neuroreport. 2015; 26: 462-466.
12. Aw ST, Todd MJ, Halmagyi GM. Latency and initiation of the human vestibuloocular reflex to pulsed galvanic stimulation. J Neurophysiol. 2006; 96: 925-30.

# Appendix 1. Dynamic Gait Index

# Appendix 2. Modified Falls Efficacy Scale


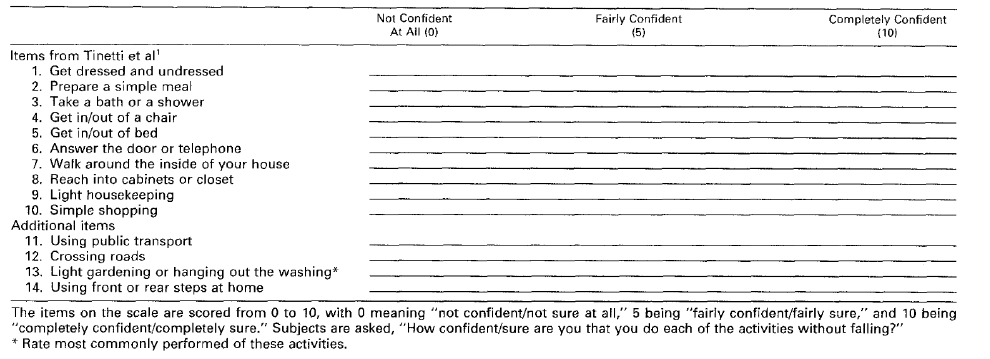


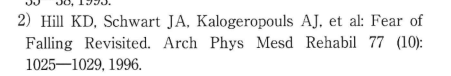


We use Japanese version in this trial.

# Appendix 3. Dizziness Handicap Inventory

P1. Does looking up increase your problem? o Yes o Sometimes o No

E2. Because of your problem, do you feel frustrated? o Yes o Sometimes o No

F3. Because of your problem, do you restrict your travel for business or recreation? o Yes o Sometimes o No

P4. Does walking down the aisle of a supermarket increase your problems? o Yes o Sometimes o No

F5. Because of your problem, do you have difficulty getting into or out of bed? o Yes o Sometimes o No

F6. Does your problem significantly restrict your participation in social activities, such as going out to dinner, going to the movies, dancing, or going to parties? o Yes o Sometimes o No

F7. Because of your problem, do you have difficulty reading? o Yes o Sometimes o No

P8. Does performing more ambitious activities such as sports, dancing, household chores (sweeping or putting dishes away) increase your problems? o Yes o Sometimes o No

E9. Because of your problem, are you afraid to leave your home without having without having someone accompany you? o Yes o Sometimes o No

E10. Because of your problem have you been embarrassed in front of others? o Yes o Sometimes o No

P11. Do quick movements of your head increase your problem? o Yes o Sometimes o No

F12. Because of your problem, do you avoid heights? o Yes o Sometimes o No

P13. Does turning over in bed increase your problem? o Yes o Sometimes o No

F14. Because of your problem, is it difficult for you to do strenuous homework or yard work? o Yes o Sometimes o No

E15. Because of your problem, are you afraid people may think you are intoxicated? o Yes o Sometimes o No

F16. Because of your problem, is it difficult for you to go for a walk by yourself? o Yes o Sometimes o No

P17. Does walking down a sidewalk increase your problem? o Yes o Sometimes o No

E18.Because of your problem, is it difficult for you to concentrate o Yes o Sometimes o No

F19. Because of your problem, is it difficult for you to walk around your house in the dark? o Yes o Sometimes o No

E20. Because of your problem, are you afraid to stay home alone? o Yes o Sometimes o No

E21. Because of your problem, do you feel handicapped? o Yes o Sometimes o No

E22. Has the problem placed stress on your relationships with members of your family or friends? o Yes o Sometimes o No

E23. Because of your problem, are you depressed? o Yes o Sometimes o No

F24. Does your problem interfere with your job or household responsibilities? o Yes o Sometimes o No

P25. Does bending over increase your problem? o Yes o Sometimes o No

(F), physical (P), and emotional (E). To each item, the following scores can be assigned: No=0 Sometimes=2 Yes=4

We use Japanese version in this trial.

Masuda K, Gotoh F, Fujii M, et al. Evaluation of the usefulness of the Dizziness Handicap Inventory (Japanese translation). *Equilibrium Res* 2004; **63**: 555-63.
