# Supplementary material for: Multicenter randomized double-blind placebo-controlled crossover study of the effect of prolonged noisy galvanic vestibular stimulation on posture or gait in vestibulopathy
Source: PLoS One. 2025 Jan 24;20(1):e0317822. doi: 10.1371/journal.pone.0317822 (PMC11760040; doi:10.1371/journal.pone.0317822)
Supplement: S5 File — (DOCX) [file pone.0317822.s005.docx]

## Supplementary Tables
